# A Quality Improvement Curriculum for Psychiatry Residents

**DOI:** 10.15766/mep_2374-8265.10870

**Published:** 2020-01-24

**Authors:** Claudia L. Reardon, Roderick Hafer, Frederick J. P. Langheim, Elliot R. Lee, Jennifer M. McDonald, Michael J. Peterson, John Stevenson, Art Walaszek

**Affiliations:** 1 Associate Professor, Department of Psychiatry, University of Wisconsin School of Medicine and Public Health; 2 Clinical Professor, Department of Psychiatry, University of Wisconsin School of Medicine and Public Health; 3 Clinical Adjunct Assistant Professor, SSM Health Dean Medical Group; 4 Clinical Adjunct Assistant Professor, Mendota Mental Health Institute; 5 Clinical Assistant Professor, William S. Middleton Memorial Veterans Hospital; 6 Associate Director for Research Services, University of Wisconsin Survey Center; 7 Professor, Department of Psychiatry, University of Wisconsin School of Medicine and Public Health

**Keywords:** Psychiatry, Quality Improvement, Patient Safety, Milestones

## Abstract

**Introduction:**

Quality improvement (QI) is an increasingly important aspect of health care and residency education. There is relatively little research describing QI curricula for residents in psychiatry. Although QI curricula have been published in *MedEdPORTAL*, the current resource represents the first such curriculum specific to psychiatry residents. This resource aims to present a QI curriculum for psychiatry residents.

**Methods:**

The University of Wisconsin psychiatry residency program implemented a QI curriculum for our PGY 3 psychiatry residents in 2010. The initial version of the curriculum has undergone marked changes over the ensuing years, reflecting feedback received from learners and faculty instructors, as well as ongoing review of the literature, to ascertain best practices in this area of medical education. Steps taken have included faculty training, development of evaluation forms, and implementation of elements to increase accountability for successful, sustainable project development.

**Results:**

During the 8 completed years of this curriculum, 77 PGY 3 psychiatry residents have completed it. The Quality Improvement Knowledge Application Tool adapted for psychiatry was completed by PGY 3 residents in advance of and upon completion of the curriculum for the first 2 years of the curriculum; results demonstrated a significant improvement in scores as a measurement of QI knowledge and skills. Thirty-one of 32 resident teams (97%) have implemented a QI project.

**Discussion:**

Our QI curriculum for PGY 3 psychiatry residents has been successful in equipping residents with QI knowledge and having them implement QI projects.

## Educational Objectives

By the end of this activity, learners will be able to:

1. Describe the principles of quality improvement (QI), including medical errors and patient safety, defining an aim, plan-do-study-act cycles, teamwork and communication, measuring patient and systems outcomes, and root cause analyses.
2. Implement a significant change in clinical inpatient or outpatient practice through the development of a QI project and measure the results of that change.

## Introduction

Quality improvement (QI) is an increasingly important aspect of health care.^1^ Pressures exist from numerous sources, including patients and public and private insurance entities, to ensure that the care physicians provide is of high quality and cost-effective.^1^ The Accreditation Council for Graduate Medical Education (ACGME) has requirements for residents to receive instruction and experience in QI.^2^ For example, ACGME common program requirements state that residents must “systematically analyze practice using QI methods, and implement changes with the goal of practice improvement” and “receive training and experience in QI processes.”^2^ Finally, to maintain board certification, residency graduates are increasingly required to participate in QI activities.^3^

Although QI curricula for residents have been published, there remains a dearth of research demonstrating that such curricula result in meaningful, sustainable QI project implementation.^4^ This is particularly true in certain specialties, including psychiatry. It could be that QI in psychiatry, including education in the topic, is relatively underdeveloped due to less obvious quality measures such as simple laboratory studies that indicate patient improvement. Research from other specialties suggests that curricula that combine both classroom and experiential components are the most effective.^5^ Accordingly, this resource aims to present a QI curriculum for psychiatry residents that includes both didactic (small-group seminars) and experiential (hands-on development of actual QI projects) elements. The curriculum strives to increase psychiatry resident knowledge in principles of QI and to develop psychiatry resident skill in researching and implementing QI projects. The current report is additionally novel in its inclusion of tangible curricular materials needed for full implementation. Although QI curricula have been published in *MedEdPORTAL*,^6–10^ this resource represents the first such curriculum specific to psychiatry residents. A specialty-specific curriculum such as this is important because residents will find it more applicable to their own practice if examples used throughout the curriculum refer directly to psychiatry-specific clinical contexts.

## Methods

With ACGME requirements to address QI within residency education newly released at the time, the University of Wisconsin psychiatry residency program began to develop a QI curriculum for our eight PGY 3 psychiatry residents in 2009. The initial (2010) version of the curriculum has undergone marked changes over the ensuing years, reflecting evaluations from learners and faculty instructors, as well as ongoing review of the literature, to ascertain best practices in this area of medical education. The final curriculum for learners involves attendance at 12.5 hours of didactic seminars and 1 half-day per week for 9 months to work on a QI project. The implementation process to get to this final curriculum involved many steps, as described next.

Several steps occurred prior to the start of the curriculum. We obtained University of Wisconsin Health Sciences Institutional Review Board exemption from formal review for this educational research project. We educated faculty members in QI so that knowledgeable mentors and instructors would be available. This included weekly Department Grand Rounds for each of the 4 weeks dedicated to QI topics, presented by local experts from within and outside the Department of Psychiatry. This month of QI Grand Rounds culminated in a 1-day education retreat on QI education within psychiatry. We next developed a set of QI didactic seminars to be delivered to PGY 3 psychiatry residents totaling 12.5 hours (Appendix A), as well as identifying faculty instructors to teach the seminars. Specific topics included Introduction to the QI Rotation (Appendix B), The Essential QI Toolbag (Appendix C), Patient Safety (Appendix D), Principles of Survey Design (Appendix E), QI in Action: A Successful Case of Improving Psychiatric Patient Care as a Resident, Root Cause Analysis, Continuing Board Certification and Performance in Practice Modules (Appendix F), Involving Stakeholders (Appendix G), and QI Journal Club (in which we reviewed an article from the psychiatry literature describing a QI project^11^ and utilized one of the *JAMA* Users’ Guides to the Medical Literature, “How to Use an Article About Quality Improvement,”^12^ to review it). These seminars were delivered during regular, protected didactic time, and all required a computer and projector. Some required or suggested readings or online modules in advance of the seminar. Instructors were psychiatry faculty and one of the directors of the University of Wisconsin Survey Center. Finally, we adapted a validated evaluation tool, the Quality Improvement Knowledge Application Tool (QIKAT),^13^ to be relevant for psychiatry, with the assistance of and testing by the original developer of the QIKAT (Appendix H). This tool assessed resident QI knowledge and skills. We also utilized our residency program's anonymous, standard rotation evaluation form, to be completed by participating residents, for this rotation.

After review of the first 4 curricular years demonstrated a need for additional resources and accountability in order for sustainably implemented, resident-led QI projects to be developed, we took several additional steps. We scheduled protected time for the experiential component of the curriculum in which residents had 1 half-day per week for 9 months to work in teams of two to three PGY 3 residents to research, implement, and study the results of a QI project. We paired each resident QI team with a QI faculty supervisor (a total of four QI supervisors per year). Supervisor meetings were scheduled for the residents, consisting of 30 minutes per week during the 9 months of protected QI time. We created and printed (on a standard printer) a QI workbook to be used by residents and their assigned QI faculty supervisors as they developed a QI project (Appendix I). This workbook included nine distinct worksheets (multiple copies of some were provided where residents would tend to need them for the duration of a single QI project). Guidance for use of these worksheets is provided in Appendix D. We scheduled midpoint (December) QI project presentations to be delivered by PGY 3 residents to the QI rotation director, program director, and other PGY 3 residents, as well as final (June) QI project presentations to be presented at Department Grand Rounds. Final QI project presentations consisted of a 30-minute oral presentation and an A3 poster presentation by each team of residents; clear guidelines for this presentation were developed and provided to residents (Appendix J). We provided an A3 poster template for use by residents (Appendix K). We also started to ask that resident QI projects bear some relationship to quality initiatives of any organization to which we were accountable, which could include our own health care system, Medicare or other payers, the Joint Commission, or other entities. Finally, we ascertained that successful completion of our QI curriculum would prospectively count as completion of an American Board of Psychiatry and Neurology (ABPN) Patient Safety Course, which became a new requirement for ABPN maintenance of certification during this time.^14^

The ACGME has developed specialty-specific milestones, representing key steps in the acquisition of foundational knowledge, skills, and attributes, culminating in competence for unsupervised practice at the completion of formal training.^15^ Milestones across specialties include items dedicated to expectations for competence in QI.^15^ With the release of ACGME psychiatry-specific milestones in 2013,^16^ we developed two ACGME milestones–based evaluation forms for our QI curriculum to further assess residents’ performance in this aspect of their training, with level 4 on our forms being the highest attainable level and representing the graduation-ready stage of accomplishment. The first such form represented the assessment by the assigned QI faculty supervisor of the residents’ participation in QI project development (Appendix L). The second such form represented the assessment by the QI curriculum director (the person who developed and oversaw this entire curriculum and participated in all elements of it) of the residents’ midpoint and final QI project presentations, their facilitation of a departmental morbidity and mortality (M&M) conference, their participation in the root cause analysis simulation, and their completion and presentation of a Performance in Practice module (Appendix M). The requirement for facilitation of an M&M conference was a new one with the release of the psychiatry milestones, which stated that residents should be developing content for and facilitating a patient safety presentation or conference focusing on systems-based errors in patient care.^16^

After 5 years of the curriculum, with the realization that new faculty hires might continually need to be educated in QI to support a culture of QI within the residency program, as well as with the desire to spread QI awareness and education throughout all 4 years of residency, the QI curriculum director undertook ongoing authoring of a QI Fact of the Week email to be sent to all psychiatry residents, residency core faculty, and QI supervisors on a recent, important, or relevant QI or patient safety issue every Friday (Appendix N).

## Results

During the 8 completed years of this curriculum, 77 PGY 3 psychiatry residents at the University of Wisconsin have completed it, and data are available from the experiences of those learners. There has been a total of six QI faculty supervisors (four during any 1 year) and a total of 10 QI didactic instructors (three to six during any 1 year). Faculty supervisors and didactic instructors have changed based on feedback from learners and availability.

QIKAT assessment tools were completed by PGY 3 residents (*N* = 16) in advance of and upon completion of the curriculum for the first 2 years. Results demonstrated a significant improvement in QIKAT average score from 4.8 to 8.1 (*p* = .0053) as a measurement of QI knowledge and skills.

Results from our residency program's anonymous, standard rotation evaluation form, as completed by participating residents, demonstrated that 74 of 77 residents (96.1%) completing the evaluation form felt that the curriculum had a positive impact on their learning and that they would change their clinical practice as a result of it. Representative qualitative feedback from the same evaluation form included the following:

• “I really enjoyed this rotation! You have an incredible amount of autonomy and can craft the project to your specifications (within reason for the time allotted). If things don't go according to plan, you have advisors to guide you, and there isn't so much pressure to ‘succeed’ that it becomes more stressful than educational. I think it is very useful to have experience with QI before entering practice, as it will certainly be part of our daily existence in the future” (Resident A).
• “The QI rotation was a wonderful exercise in coming [up] with practice improvement areas and creating a tangible plan to work on them while inculcating the core QI principles. It was also a lesson in teamwork and working with stakeholders and a project supervisor. [Faculty] put in a great effort on providing quality materials and didactics. [Faculty] were also very supportive of the various projects and presentations” (Resident B).
• “I'm so happy this program offers a longitudinal QI rotation. It's really the only way we could accomplish anything meaningful. This may sound odd, but one of the most helpful things about this rotation is being able to learn why some of things that drive you nuts about residency are the way they are, how they came to be that way, and how they could be changed. It helps to bolster a feeling of agency and involvement” (Resident C).
• “The didactics were spot on and made great use of multiple forms of learning. It was evident that much time and effort went into this and it was most appreciated. This is obviously an ever-growing part of our future practice and I'm honored to have such great training during residency and feel that this specific aspect of our program really sets it apart from others, and I'm proud to speak of this to both applicants and friends in other programs. I also appreciate the independence and guidance along the way. I feel that as [maintenance of licensure/maintenance of certification] guidelines involve, I will be more than ready to adapt to the changes” (Resident D).

We completed our ACGME milestones–based resident evaluation forms of QI performance for the most recent 3 years of the curriculum (*N* = 20). Results demonstrated that on the milestones-based assessment by the QI faculty supervisors, 67% of residents achieved the maximum possible level, level 4 (“substantially contributes to a supervised project to address a specific quality deficit in clinical practice and measures relevant outcomes”); the remaining residents were awarded credit at level 3 or 3.5. On the milestones-based assessment by the QI curriculum director, 75% of residents achieved the maximum possible level, level 4, on the main question (“discusses quality gaps and problems with psychiatric care delivery; outlines factors and causal chains contributing to quality gaps; lists details of multiple barriers encountered in QI project and strategies for getting stakeholder buy-in from those affected by the project; describes details of methods for implementation [including at least one discrete QI tool such as a plan-do-check-act cycle] and evaluation [including comparison of baseline and final data] of a clinical QI project”), and the remaining 25% were awarded credit at level 3.5 (indicating accomplishment in most but not all of those measures). Thirty-one of 32 resident teams (97%) have implemented a QI project, with implementation defined as incorporation into clinical practice. The Table depicts the most common categories of projects and examples within each category.

**Table.** Most Common Categories of Psychiatry Resident Quality Improvement Projects and Example Projects Within Each of the Categories

Project Theme	No. of Projects	Example Projects
Improvement of outpatient diagnosis or management of psychiatric patients	7	• Optimization of assessment for tobacco use disorder in psychiatric outpatients • Development of a system for completion of mental health rating scales in the waiting area prior to outpatient psychiatry appointments
Teamwork/communication	4	• Improvement in communication between outpatient front desk staff and psychiatry residents regarding patient phone calls • Improvement in assessment of language barriers for patients with limited English proficiency in outpatient psychiatry clinic
Consultation/liaison psychiatry initiatives	3	• Development of a new inpatient behavioral health consultation order set to optimize appropriateness of consultations to psychiatry versus health psychology versus addiction consult services • Optimization of inpatient decisional capacity consultation process
Improvement of inpatient diagnosis or management of psychiatric patients	3	• Optimization of appropriate nicotine replacement orders at time of psychiatric inpatient admission • Improvement in efficiency and timeliness of evidence-based sleep interventions at time of psychiatric inpatient admission

One hundred percent of resident teams participating in this curriculum have presented their QI projects at rotation midpoint and at final Grand Rounds time slots since the implementation of requirements for these presentations. Additionally, our residents’ QI projects have been presented in nine statewide, regional, and national poster symposia and two journals,^4,17^ and one has received grant dollars for national implementation of it within the Veterans Affairs hospital system.^18^

## Discussion

Our QI curriculum for PGY 3 psychiatry residents has been successful in its aims of equipping residents with knowledge of QI principles, having them implement QI projects in clinical practice, and having them present the results of their work. Success in these measures appears to have been a product of implementation of best practices extrapolated from the nonpsychiatric literature,^19^ as well as study of our curriculum and refinement of its elements. We have also addressed challenges encountered during the course of this curriculum. These challenges have included training of faculty in QI so that they could serve as teachers and mentors within the curriculum and contribute to a culture of QI within the residency program, promotion of steady progress in QI project development during an allotted QI project time such that residents did not wait until the end of the 9 months to work on a project, incentivization of meaningful completion of all curriculum activities, and encouragement of QI projects that not only were meaningful and relevant to resident work life but also would likely be of interest to health care system leaders. We felt the latter to be important so that projects could represent preparation for postresidency work life by helping residents understand the importance of stakeholder buy-in, especially if they wished to have future protected time from their employers for QI project work.

Based on consideration of all of these factors, several curricular elements appear to have aided in the success of this curriculum. Specifically, we provided initial faculty education in QI and continue to provide such education. We utilize a relatively small number of learners per didactic session (typically, nine) and per faculty project supervisor (two to three) to allow for significant participation by each learner in these sessions.

Timing and scheduling aspects associated with the rotation also appear to be important to its success. Residents receive protected time for project development, including incorporation of elements that lend structure and promote steady project completion (e.g., automatically scheduled meeting times with faculty project supervisors during protected time, use of a QI workbook to guide project development, and required midpoint and end-of-year project presentations). The time provided to residents (i.e., 9 months) for project development is longitudinal as opposed to a shorter block of time such as the common rotation length of 1 month.^19^

Residents are further incentivized to develop QI projects that are of high quality. Specifically, they are awarded ABPN Patient Safety Course credit only if all curricular activities are completed successfully. Residents are allowed to choose their own QI projects, but curriculum evaluation forms reward selection of projects that address quality initiatives of any of a number of entities to which they are accountable. Curriculum evaluation forms also reward implementation of projects in a manner designed to be sustainable even after the residents have completed their QI curriculum. Finally, residents utilize an A3 poster template that is widely recognized as a standard way of presenting QI project results such that projects can be presented in scholarly venues beyond our residency program. Without much additional work beyond that required for this curriculum, the residents have a ready-made, curriculum vitae–worthy scholarly product to present elsewhere.

The ultimate value of this curriculum may lie in preparation of our residents for psychiatric practice beyond residency. They will hopefully be prepared for participation in a health care system that demands quality outcomes and for meeting board certification requirements that may be important for participation in this system.

Limitations of this curriculum include that it requires a relatively large amount of resourcing for its implementation and sustainability, measured in terms of faculty and resident time. Thus, there may be some programs unable to implement it in its full form. Evaluation of the curriculum over time, including at time points when it was not as fully developed and refined, suggests that partial implementation of the curriculum would likely have some benefit. Elements described previously should be prioritized. These elements may be useful for residency accreditation bodies to consider as they develop any future specific recommendations for residency programs’ QI curricula.

Future directions may include study of this curriculum within other training programs, including ones representing a diversity of specialties, practice settings, and levels of resourcing. The QIKAT as developed specifically for psychiatry would not be relevant for all specialties, but other iterations of it exist, and new specialty-specific versions could be developed. Within our own program's implementation of this curriculum, we aspire to develop elements that particularly focus on quality initiatives that might address health care disparities given the new ACGME Program Requirements for Graduate Medical Education in Psychiatry, which state that “residents must receive training and experience in QI processes, including an understanding of health care disparities.”^20^ Ultimately, it would be ideal to know if and how this curriculum impacts future psychiatric practice, specifically, quality of patient care.

## Appendices

A. QI Didactic Seminars.doc
B. Introduction to the QI Rotation Slides.ppt
C. Essential QI Toolbag Slides.ppt
D. Patient Safety Slides.ppt
E. Principles of Survey Design Slides.pptx
F. CBC and PIP Modules Slides.pptx
G. Involving Stakeholders Slides.ppt
H. QIKAT for Psychiatry.doc
I. QI Workbook.doc
J. QI Final Presentation Guidelines.doc
K. A3 QI Poster Template 11x17.pptx
L. QI Supervisor Evaluation of Resident.docx
M. QI Director Evaluation of Resident.pdf
N. QI Facts of the Week Sample.docx All appendices are peer reviewed as integral parts of the Original Publication.
