# Supplementary material for: A Quality Improvement Curriculum for Psychiatry Residents
Source: MedEdPORTAL. 2020 Jan 24;16:10870. doi: 10.15766/mep_2374-8265.10870 (PMC7012317; doi:10.15766/mep_2374-8265.10870)
Supplement: Supplementary file 1 — A. QI Didactic Seminars.doc B. Introduction to the QI Rotation Slides.ppt C. Essential QI Toolbag Slides.ppt D. Patient Safety Slides.ppt E. Principles of Survey Design Slides.pptx F. CBC and PIP Modules Slides.pptx G. Involving Stakeholders Slides.ppt H. QIKAT for Psychiatry.doc I. QI Workbook.doc J. QI Final Presentation Guidelines.doc K. A3 QI Poster Template 11x17.pptx L. QI Supervisor Evaluation of Resident.docx M. QI Director Evaluation of Resident.pdf N. QI Facts of the Week Sample.docx [file mep-16-10870-s001.zip › E. Principles of Survey Design Slides.pptx]

## Slide 1
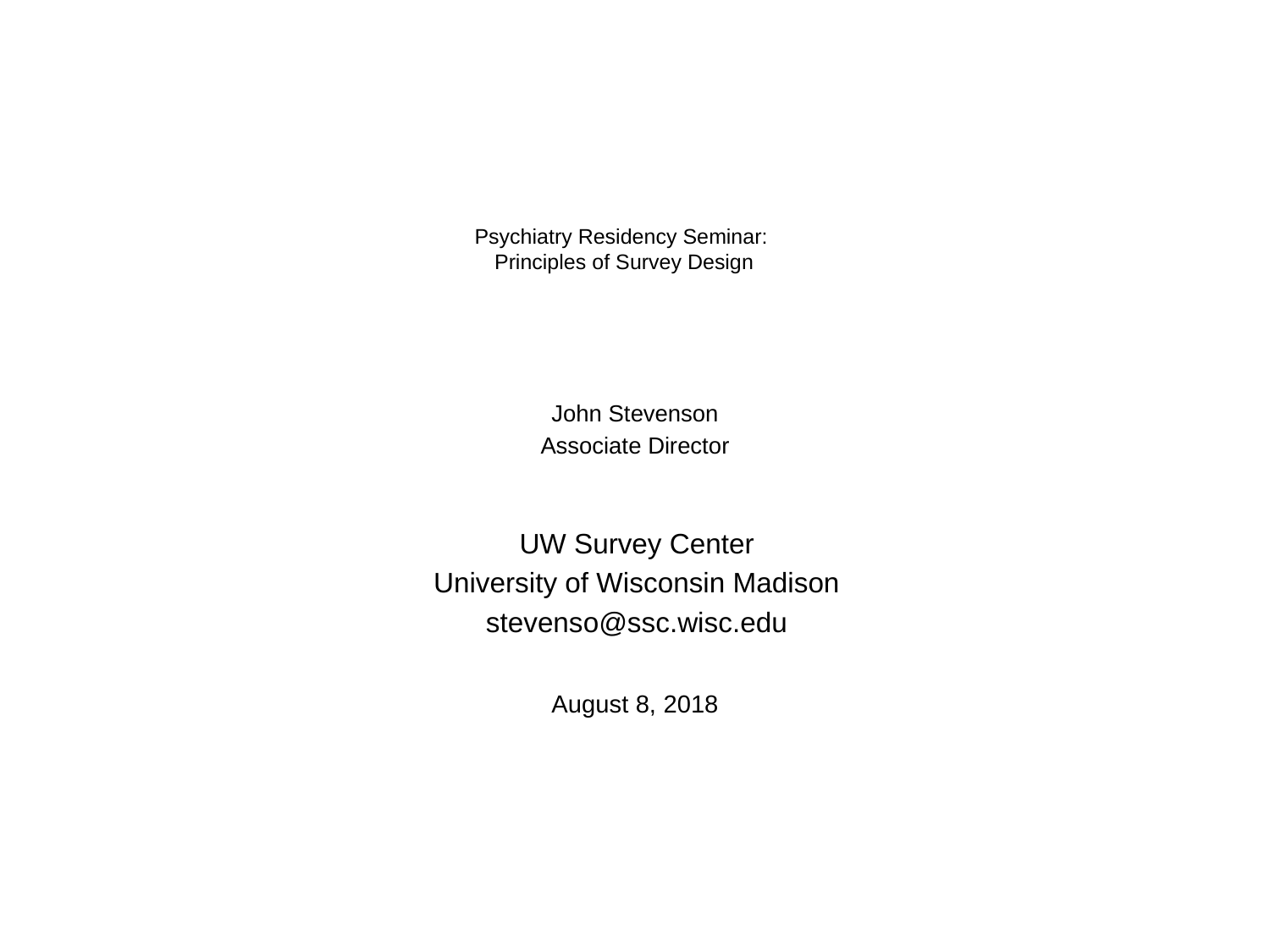

# Psychiatry Residency Seminar: Principles of Survey Design
John Stevenson
Associate Director
UW Survey Center
University of Wisconsin Madison
stevenso@ssc.wisc.edu
August 8, 2018

## Slide 2
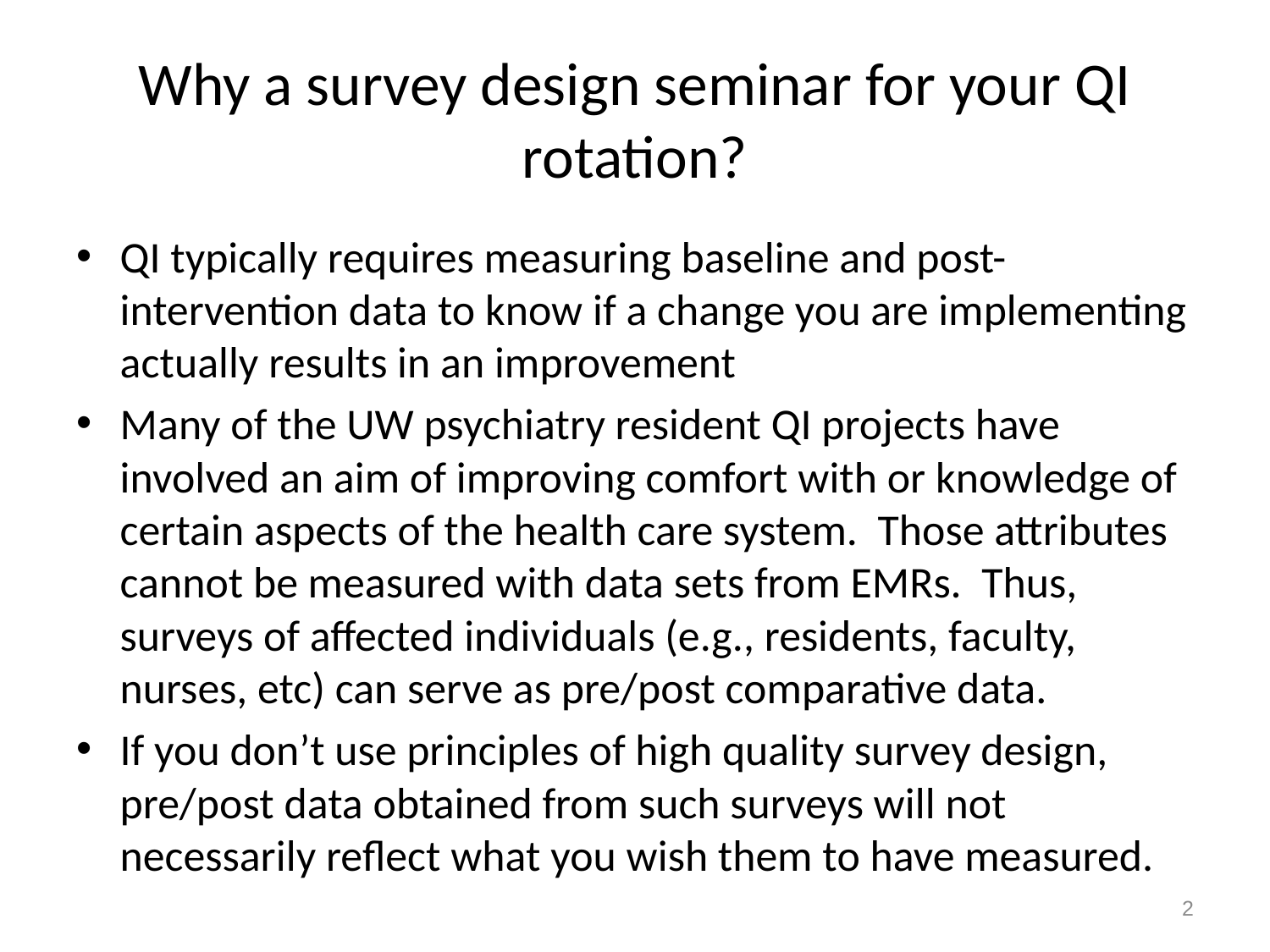

# Why a survey design seminar for your QI rotation?
QI typically requires measuring baseline and post-intervention data to know if a change you are implementing actually results in an improvement
Many of the UW psychiatry resident QI projects have involved an aim of improving comfort with or knowledge of certain aspects of the health care system. Those attributes cannot be measured with data sets from EMRs. Thus, surveys of affected individuals (e.g., residents, faculty, nurses, etc) can serve as pre/post comparative data.
If you don’t use principles of high quality survey design, pre/post data obtained from such surveys will not necessarily reflect what you wish them to have measured.
2

## Slide 3
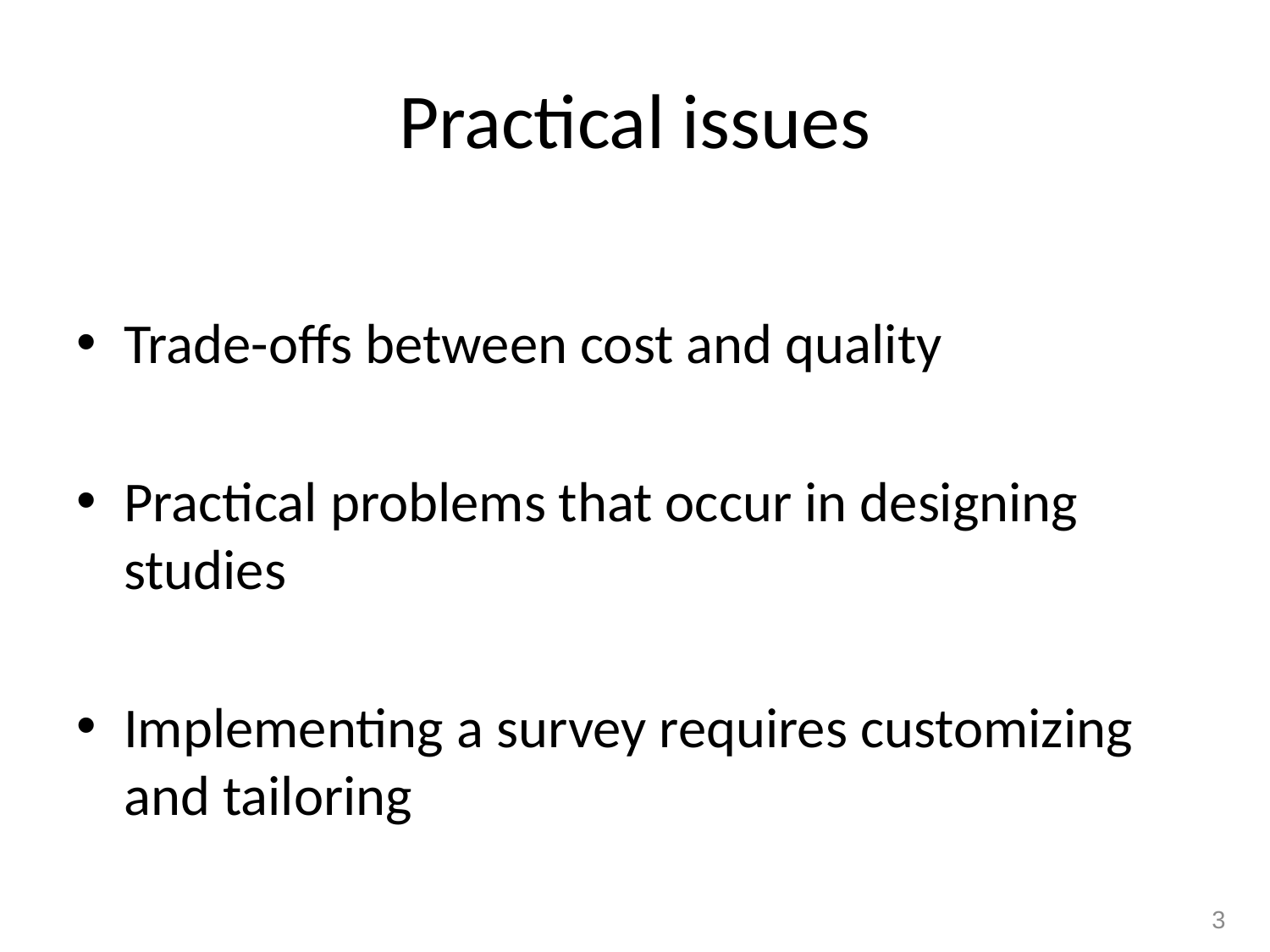

# Practical issues
Trade-offs between cost and quality
Practical problems that occur in designing studies
Implementing a survey requires customizing and tailoring
3

## Slide 4
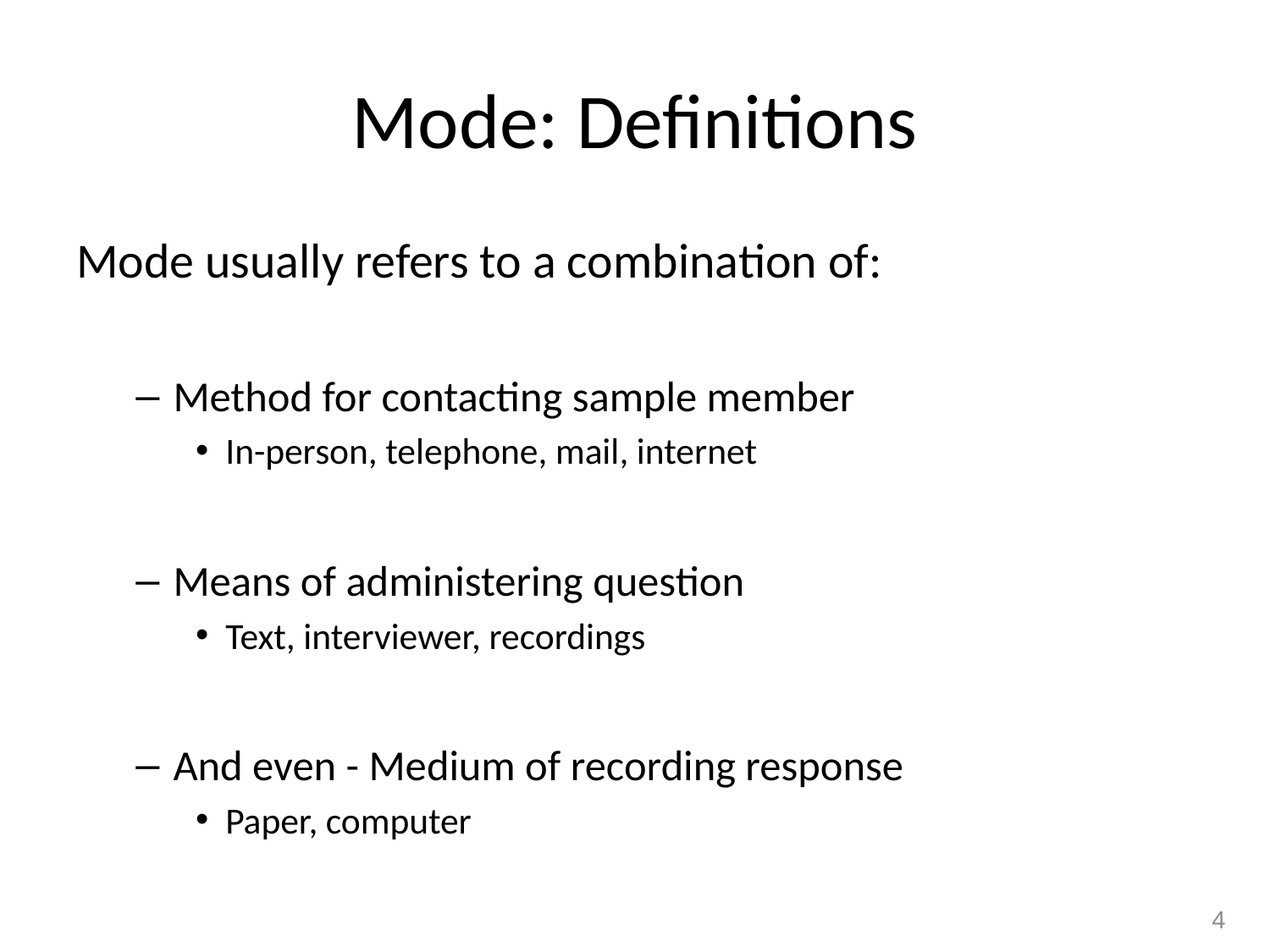

# Mode: Definitions
Mode usually refers to a combination of:
Method for contacting sample member
In-person, telephone, mail, internet
Means of administering question
Text, interviewer, recordings
And even - Medium of recording response
Paper, computer
4

## Slide 5
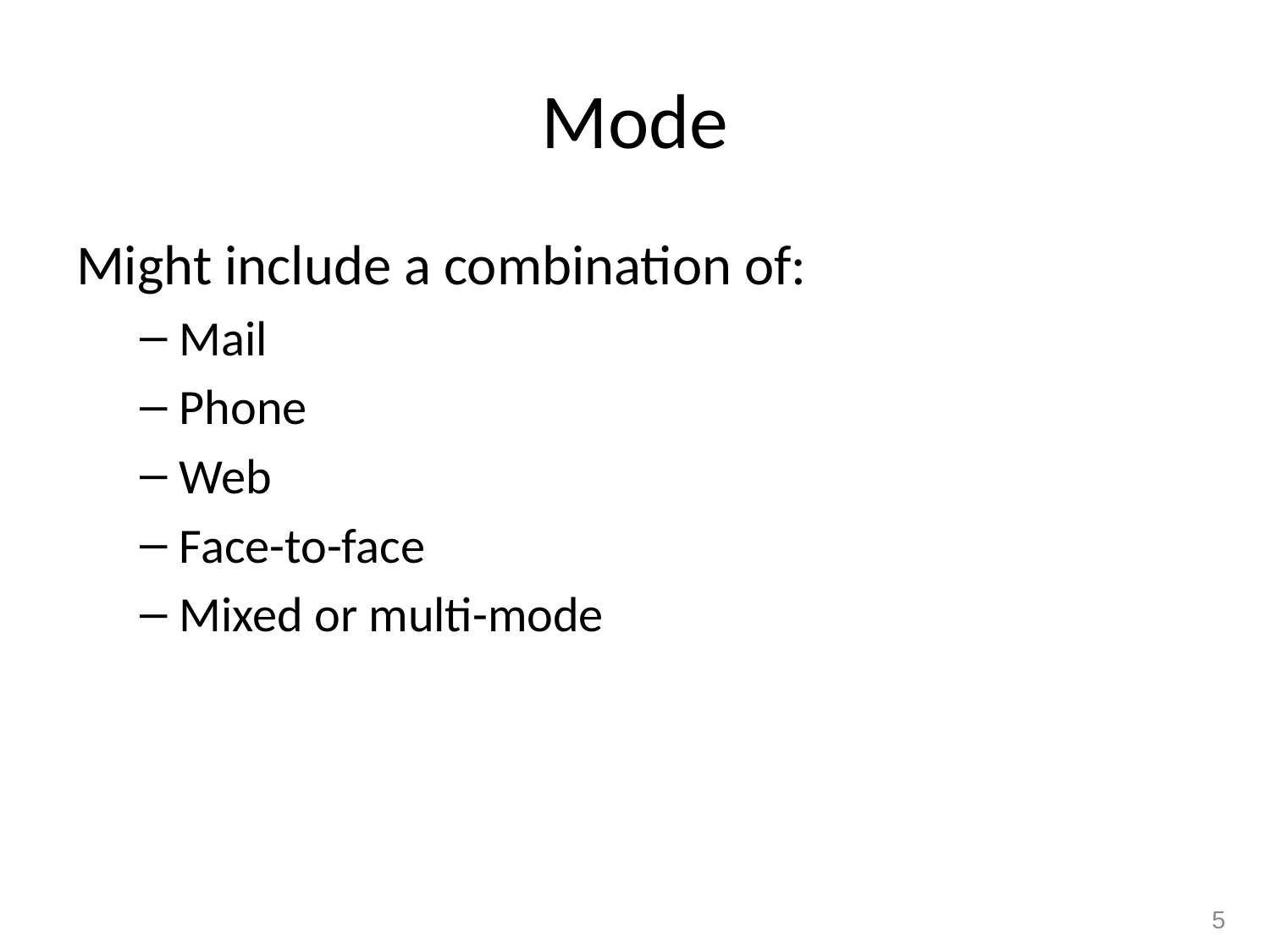

# Mode
Might include a combination of:
Mail
Phone
Web
Face-to-face
Mixed or multi-mode
5

## Slide 6
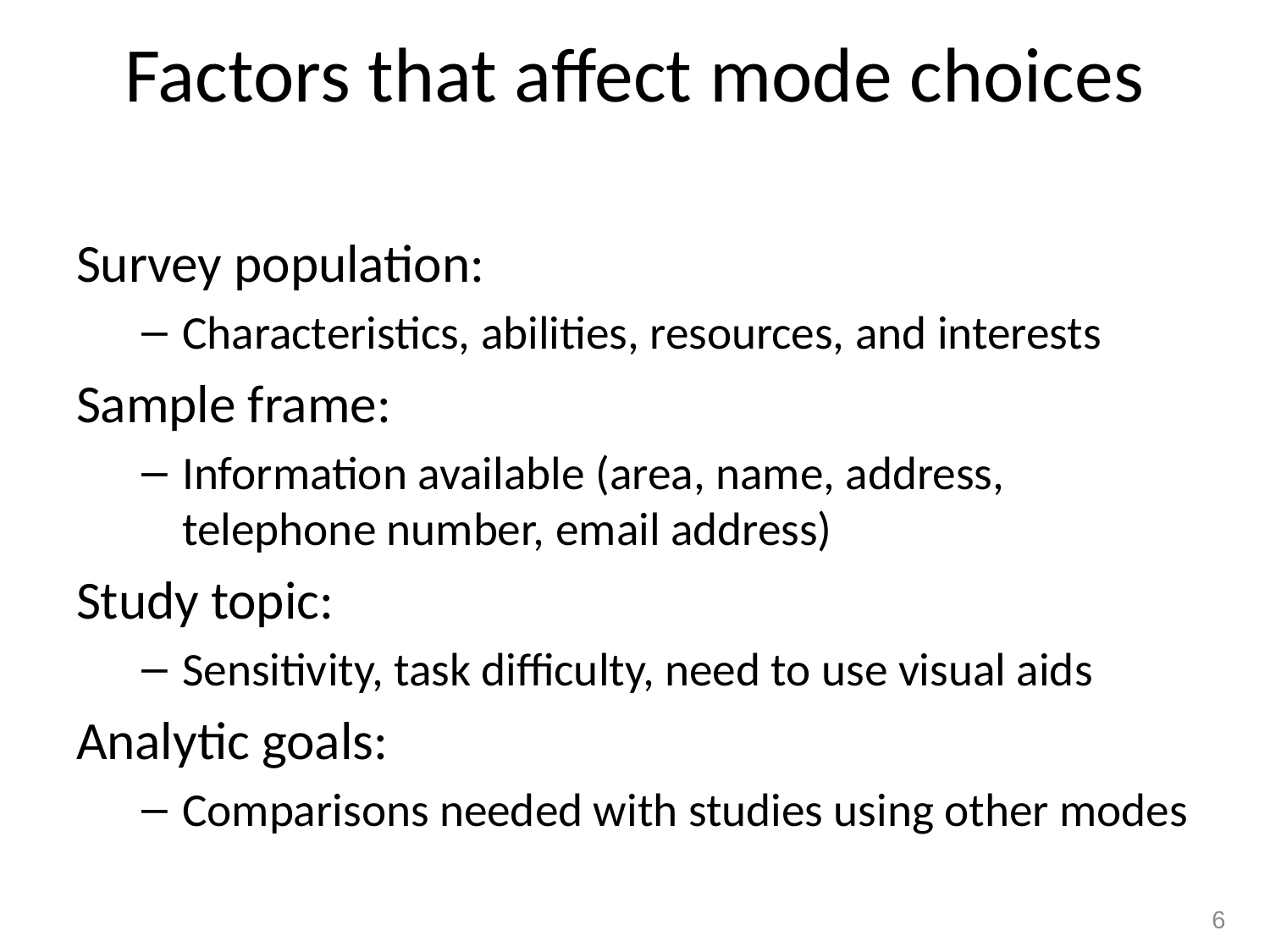

# Factors that affect mode choices
Survey population:
Characteristics, abilities, resources, and interests
Sample frame:
Information available (area, name, address, telephone number, email address)
Study topic:
Sensitivity, task difficulty, need to use visual aids
Analytic goals:
Comparisons needed with studies using other modes
6

## Slide 7
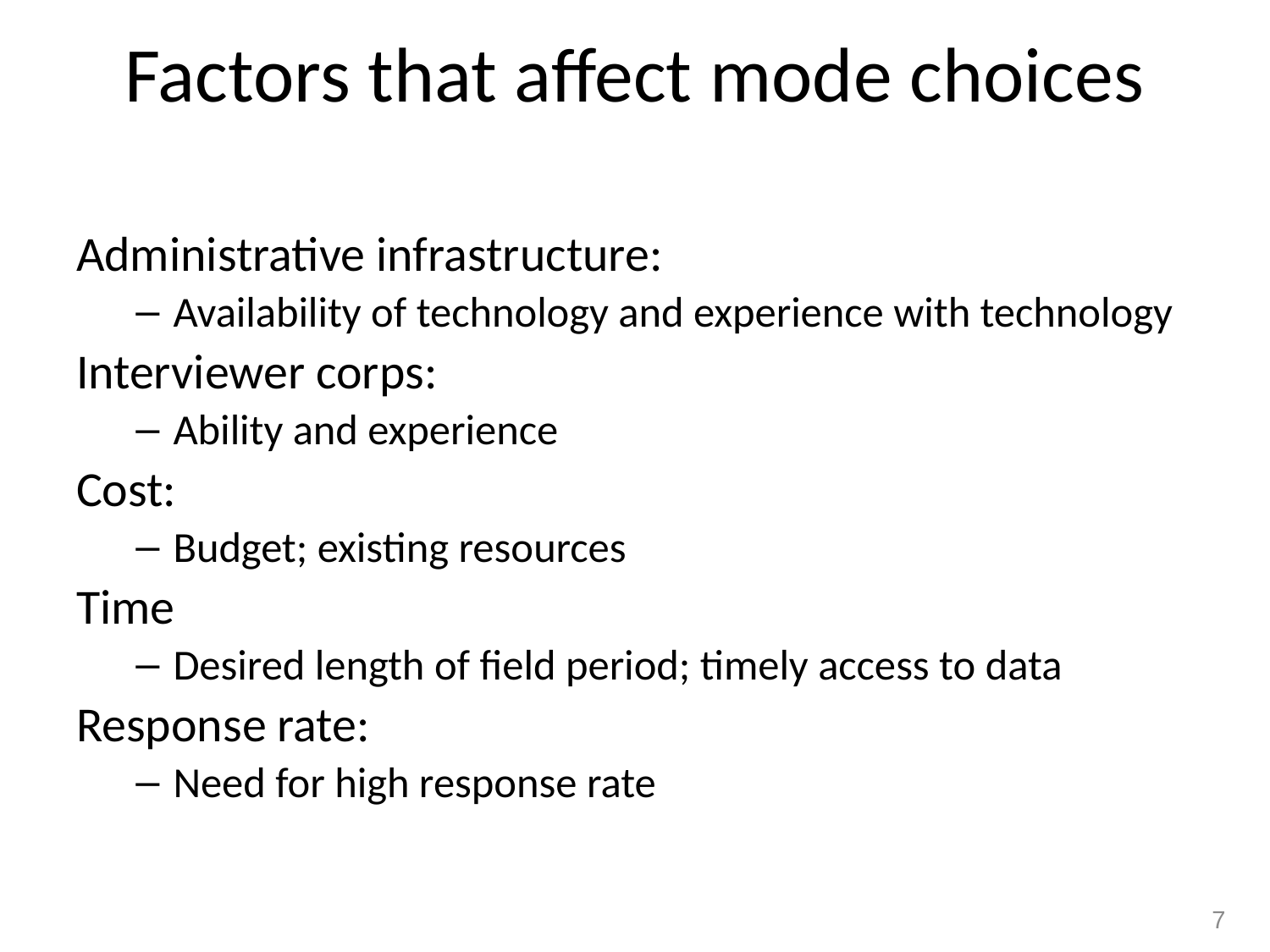

# Factors that affect mode choices
Administrative infrastructure:
Availability of technology and experience with technology
Interviewer corps:
Ability and experience
Cost:
Budget; existing resources
Time
Desired length of field period; timely access to data
Response rate:
Need for high response rate
7

## Slide 8
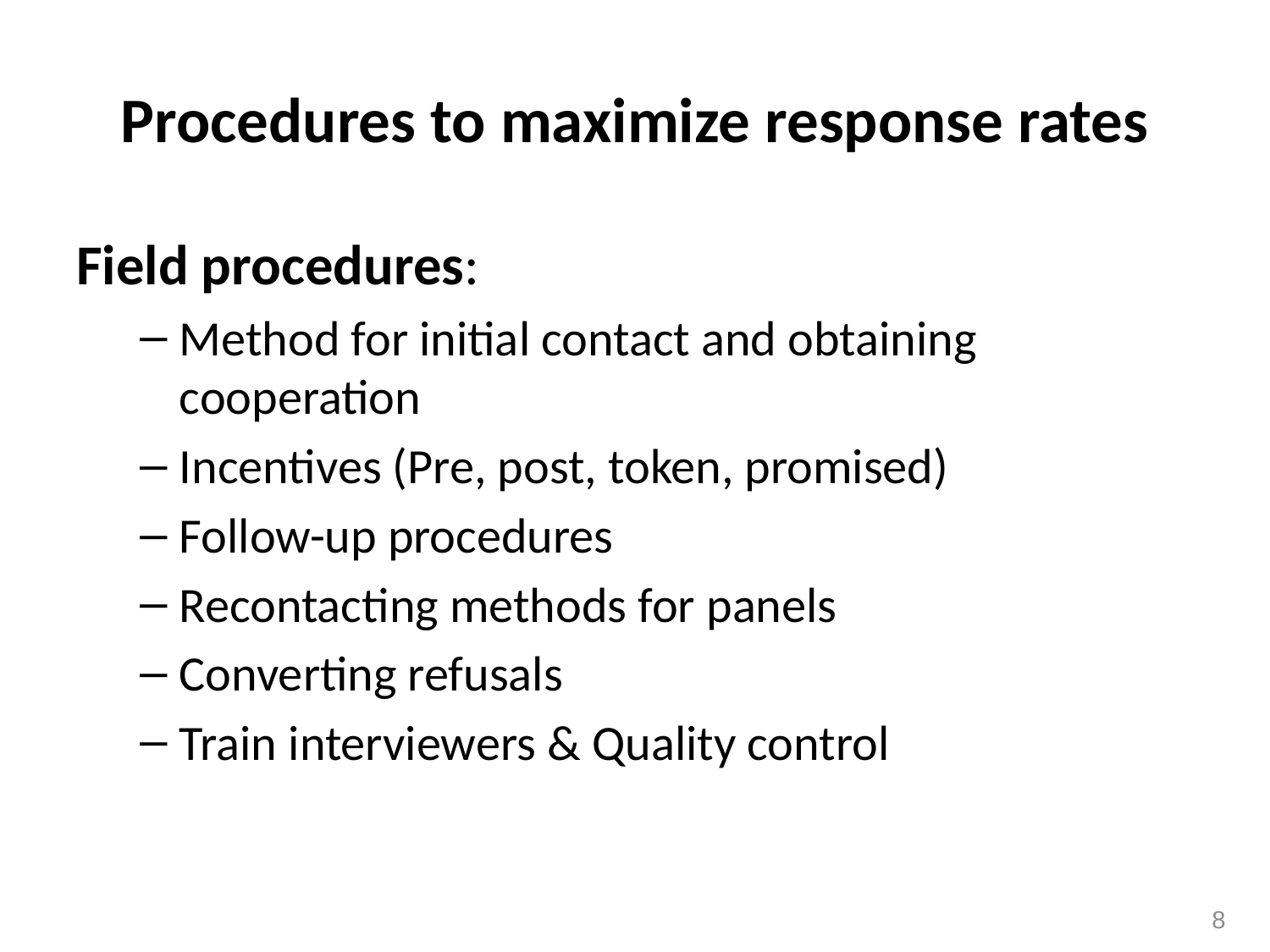

# Procedures to maximize response rates
Field procedures:
Method for initial contact and obtaining cooperation
Incentives (Pre, post, token, promised)
Follow-up procedures
Recontacting methods for panels
Converting refusals
Train interviewers & Quality control
8

## Slide 9
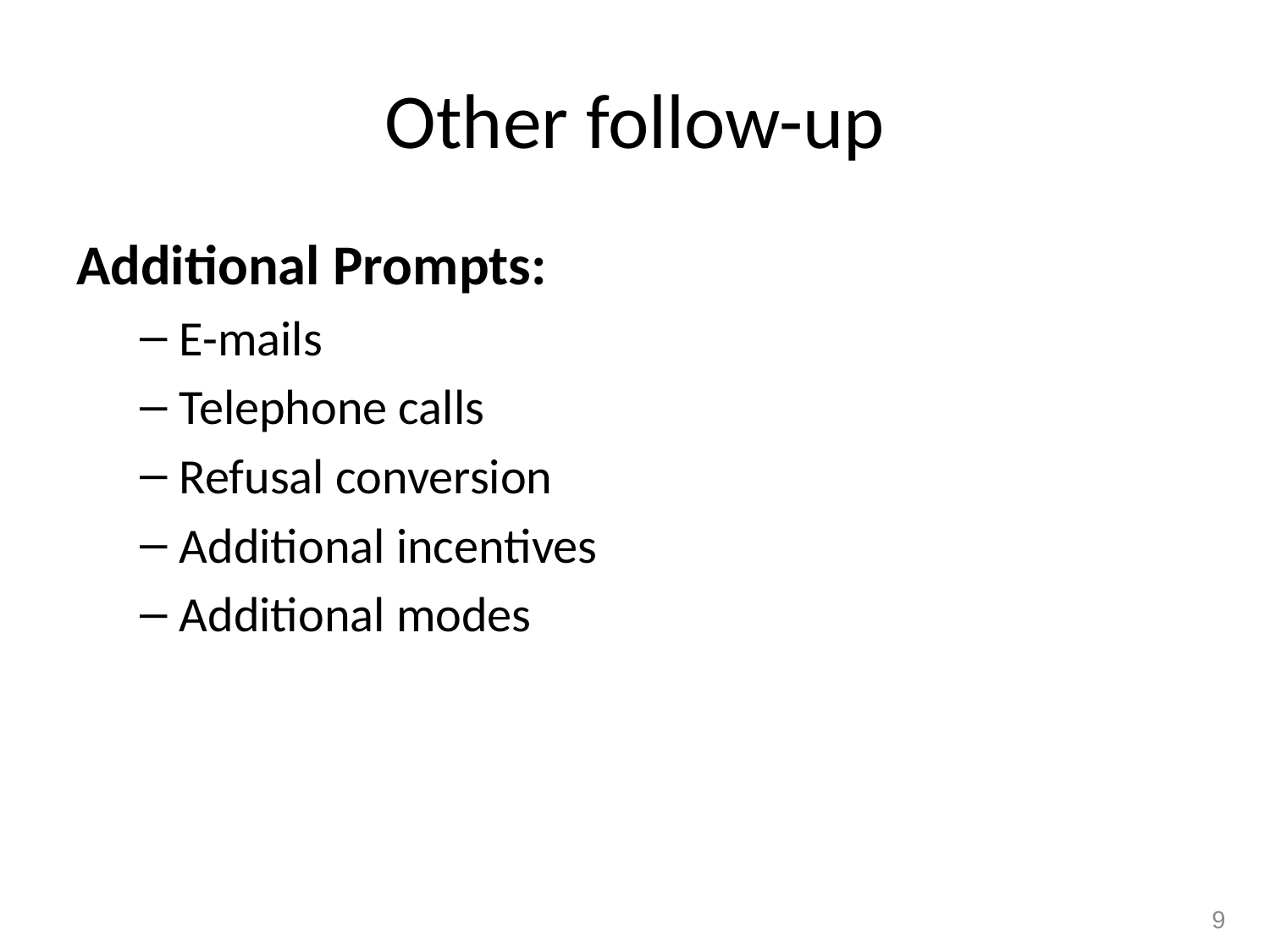

# Other follow-up
Additional Prompts:
E-mails
Telephone calls
Refusal conversion
Additional incentives
Additional modes
9

## Slide 10
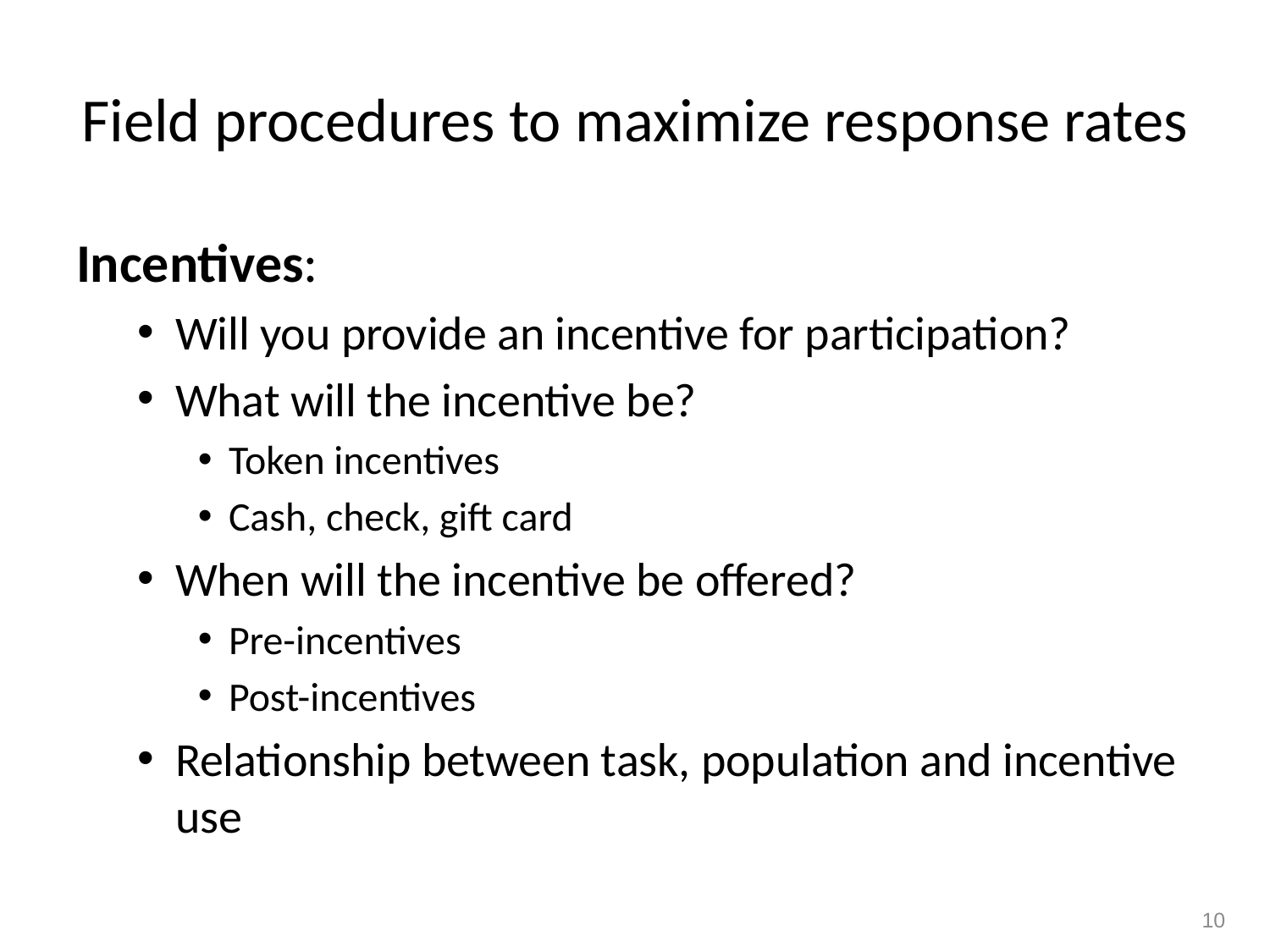

# Field procedures to maximize response rates
Incentives:
Will you provide an incentive for participation?
What will the incentive be?
Token incentives
Cash, check, gift card
When will the incentive be offered?
Pre-incentives
Post-incentives
Relationship between task, population and incentive use
10

## Slide 11
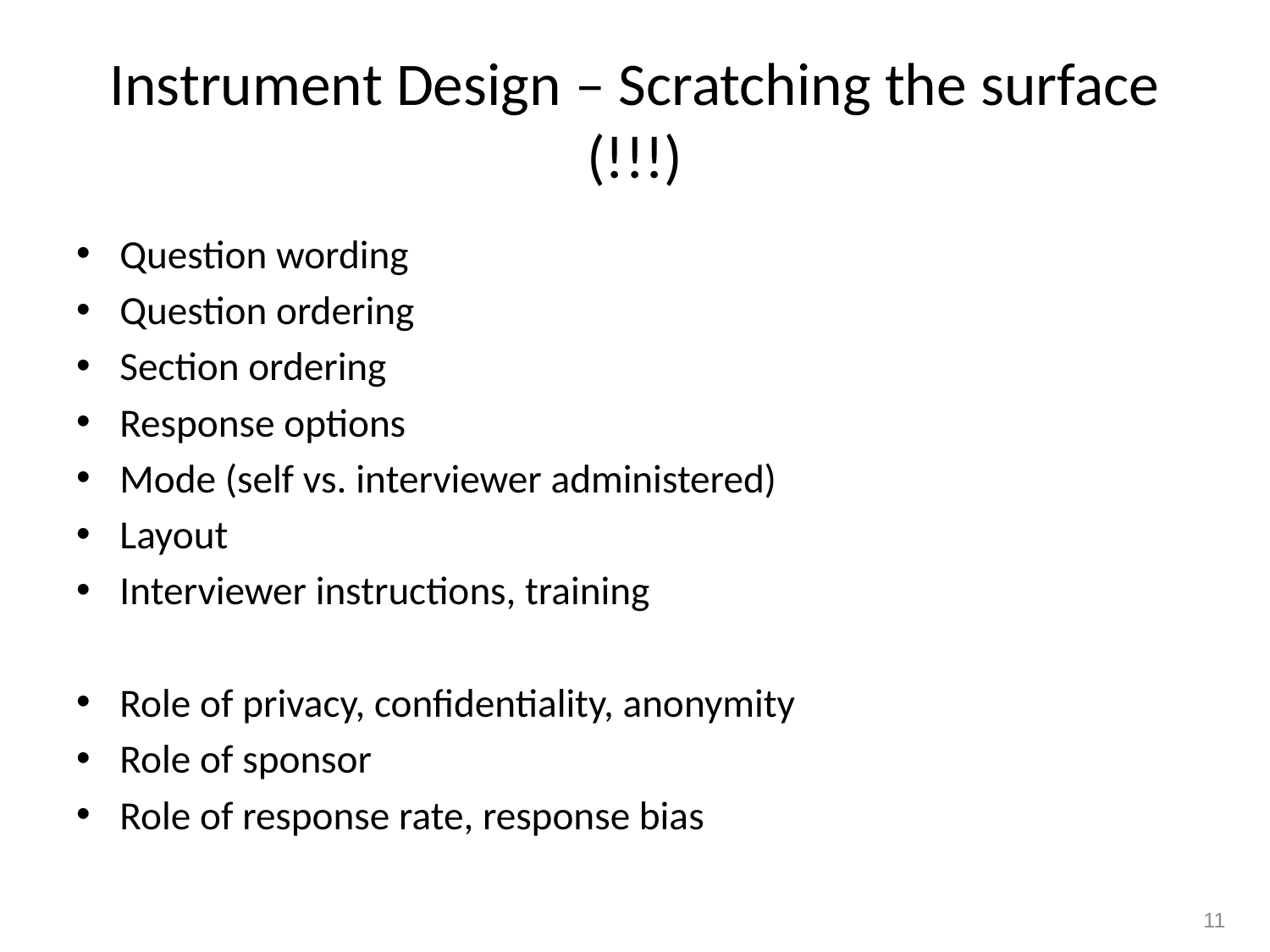

# Instrument Design – Scratching the surface (!!!)
Question wording
Question ordering
Section ordering
Response options
Mode (self vs. interviewer administered)
Layout
Interviewer instructions, training
Role of privacy, confidentiality, anonymity
Role of sponsor
Role of response rate, response bias
11

## Slide 12
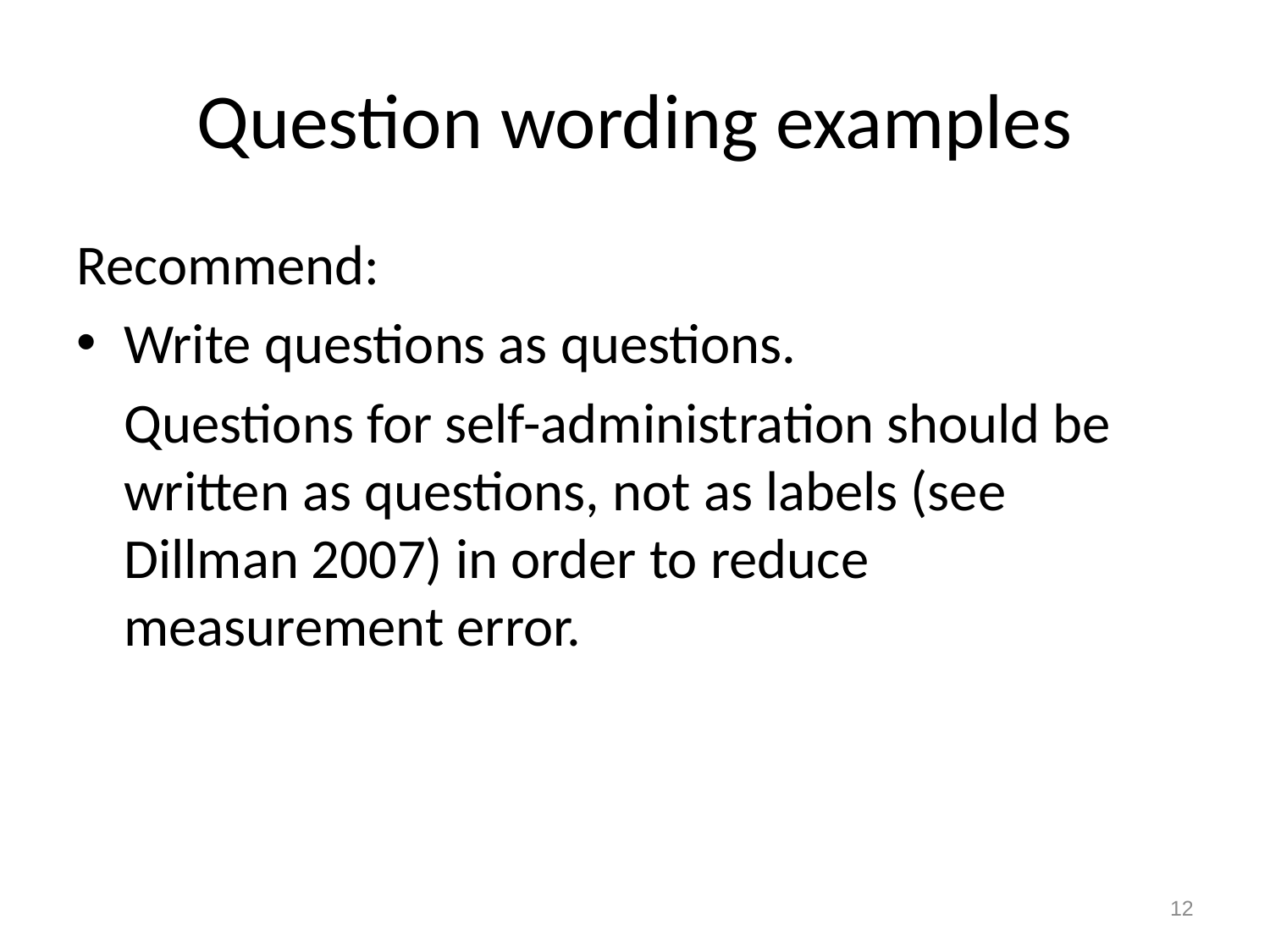

# Question wording examples
Recommend:
Write questions as questions.
	Questions for self-administration should be written as questions, not as labels (see Dillman 2007) in order to reduce measurement error.
12

## Slide 13
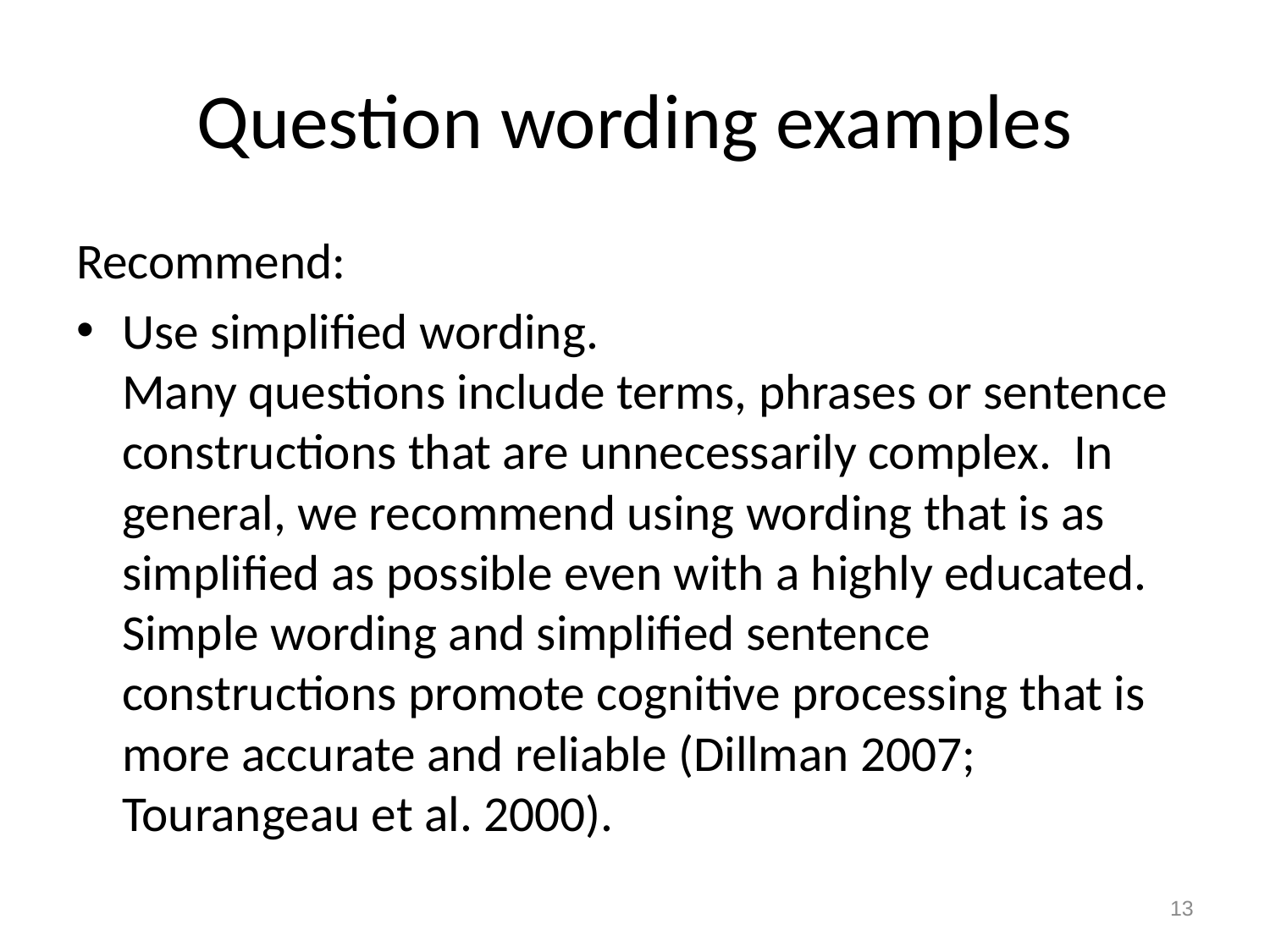

# Question wording examples
Recommend:
Use simplified wording.
Many questions include terms, phrases or sentence constructions that are unnecessarily complex. In general, we recommend using wording that is as simplified as possible even with a highly educated. Simple wording and simplified sentence constructions promote cognitive processing that is more accurate and reliable (Dillman 2007; Tourangeau et al. 2000).
13

## Slide 14
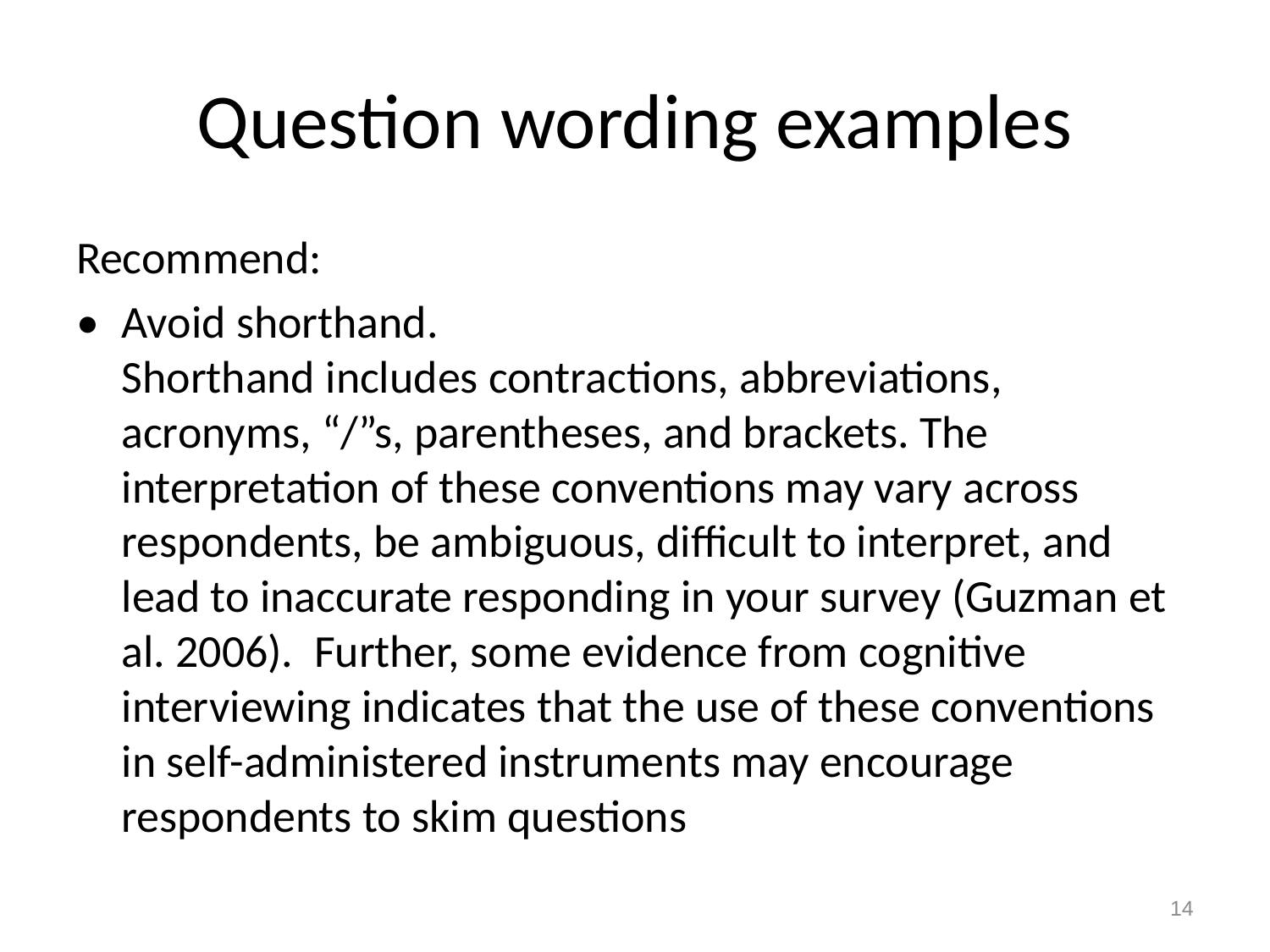

# Question wording examples
Recommend:
•	Avoid shorthand.
Shorthand includes contractions, abbreviations, acronyms, “/”s, parentheses, and brackets. The interpretation of these conventions may vary across respondents, be ambiguous, difficult to interpret, and lead to inaccurate responding in your survey (Guzman et al. 2006). Further, some evidence from cognitive interviewing indicates that the use of these conventions in self-administered instruments may encourage respondents to skim questions
14

## Slide 15
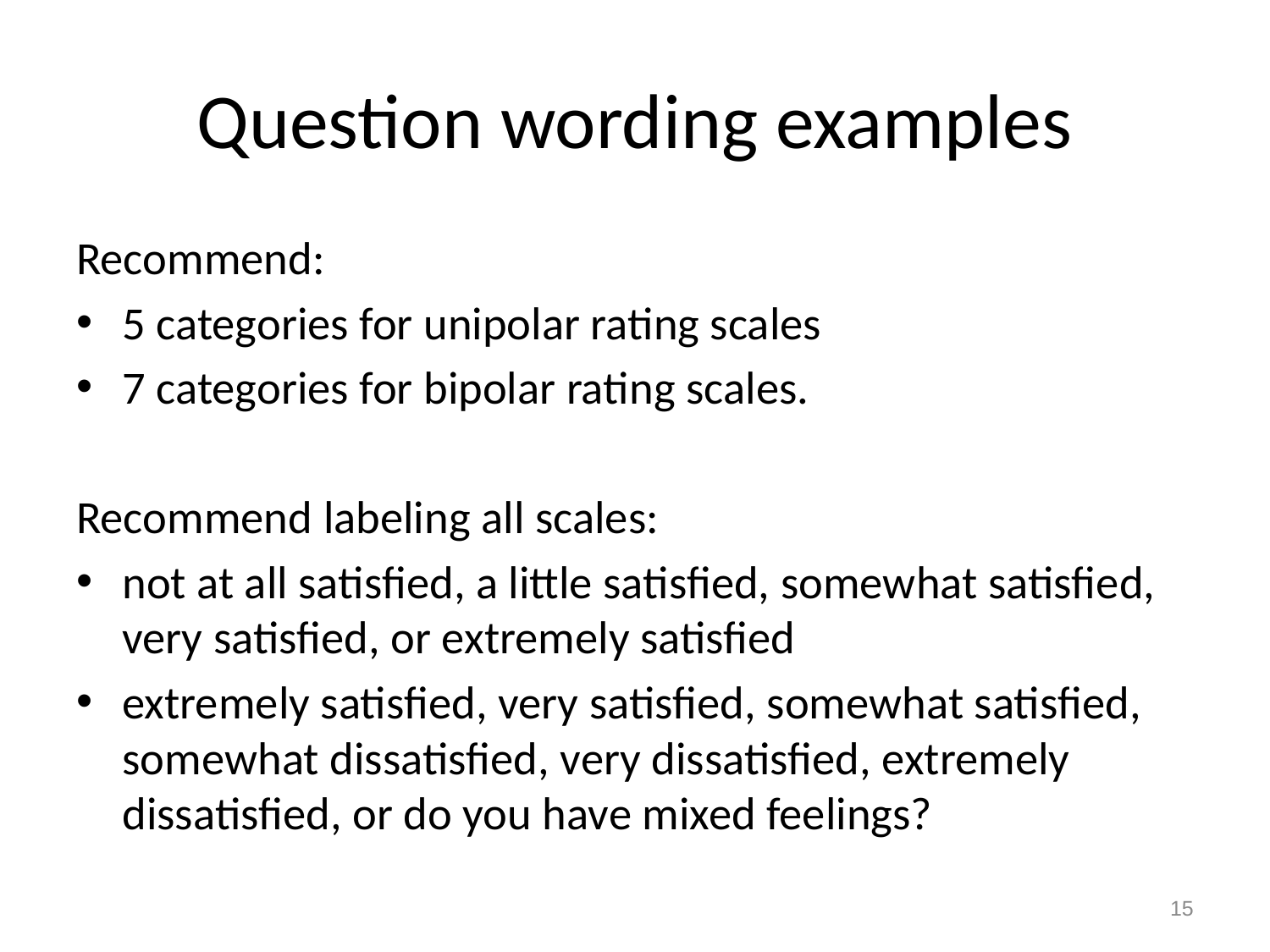

# Question wording examples
Recommend:
5 categories for unipolar rating scales
7 categories for bipolar rating scales.
Recommend labeling all scales:
not at all satisfied, a little satisfied, somewhat satisfied, very satisfied, or extremely satisfied
extremely satisfied, very satisfied, somewhat satisfied, somewhat dissatisfied, very dissatisfied, extremely dissatisfied, or do you have mixed feelings?
15

## Slide 16
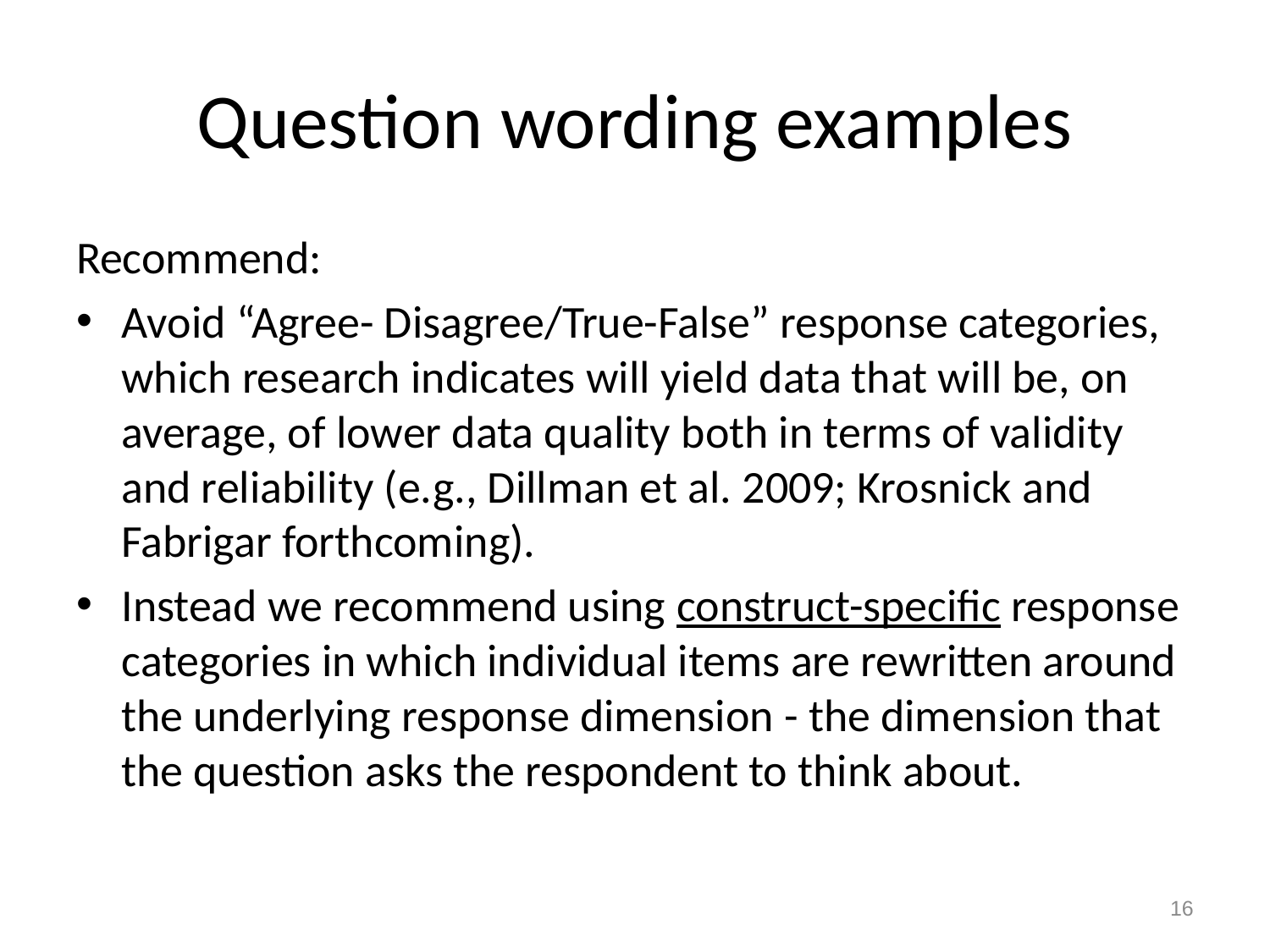

# Question wording examples
Recommend:
Avoid “Agree- Disagree/True-False” response categories, which research indicates will yield data that will be, on average, of lower data quality both in terms of validity and reliability (e.g., Dillman et al. 2009; Krosnick and Fabrigar forthcoming).
Instead we recommend using construct-specific response categories in which individual items are rewritten around the underlying response dimension - the dimension that the question asks the respondent to think about.
16

## Slide 17
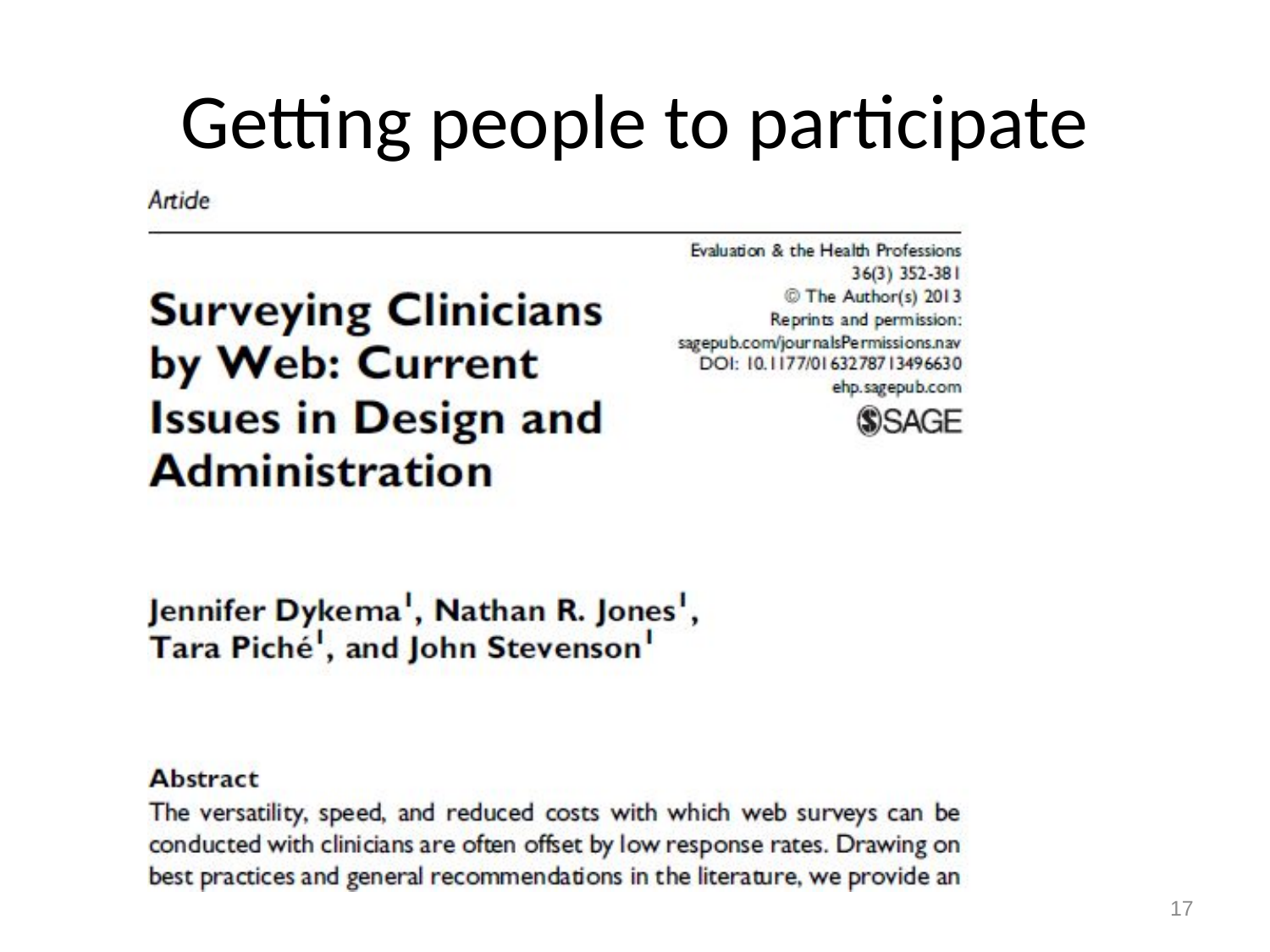

# Getting people to participate
17

## Slide 18
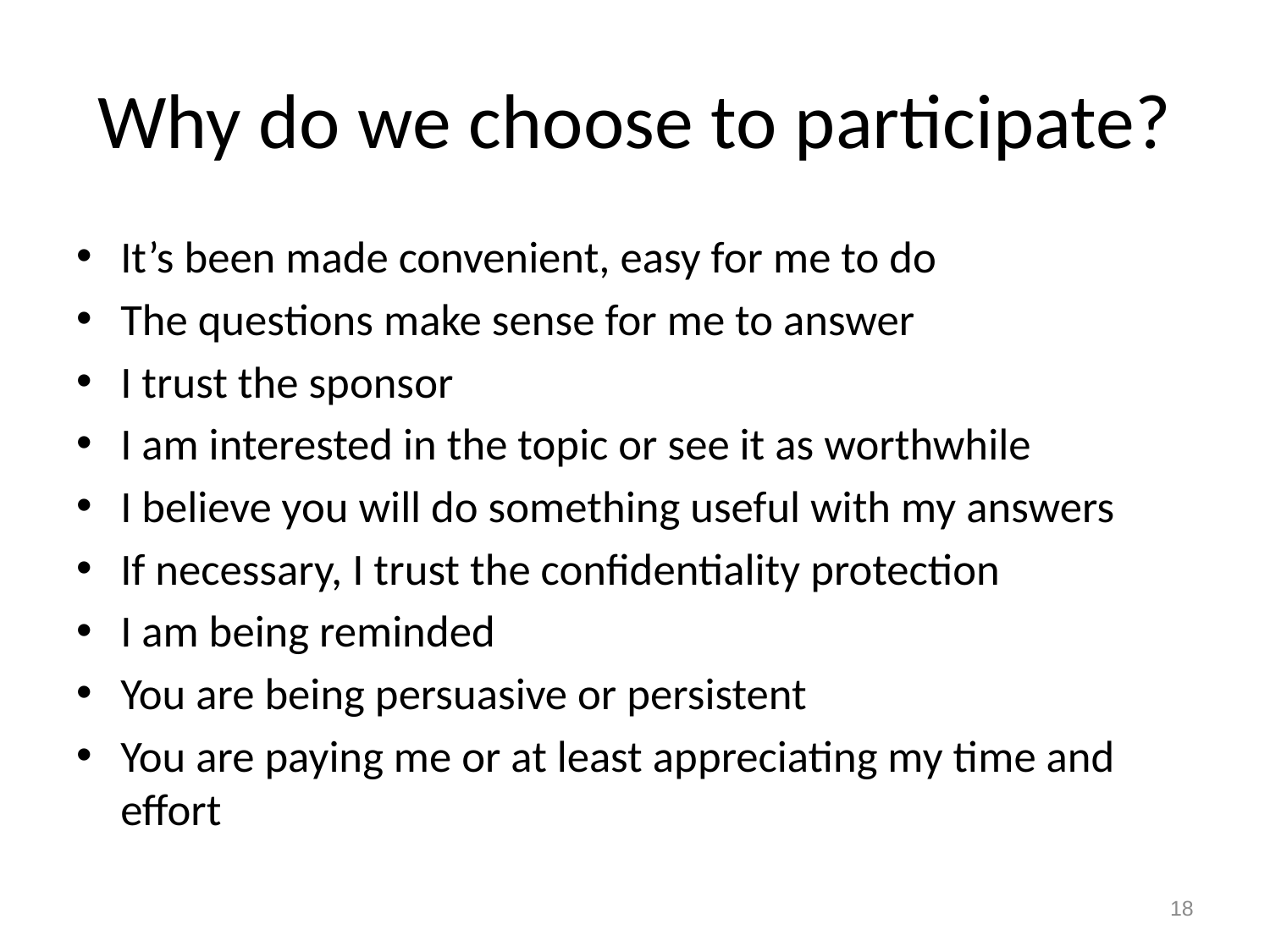

# Why do we choose to participate?
It’s been made convenient, easy for me to do
The questions make sense for me to answer
I trust the sponsor
I am interested in the topic or see it as worthwhile
I believe you will do something useful with my answers
If necessary, I trust the confidentiality protection
I am being reminded
You are being persuasive or persistent
You are paying me or at least appreciating my time and effort
18

## Slide 19
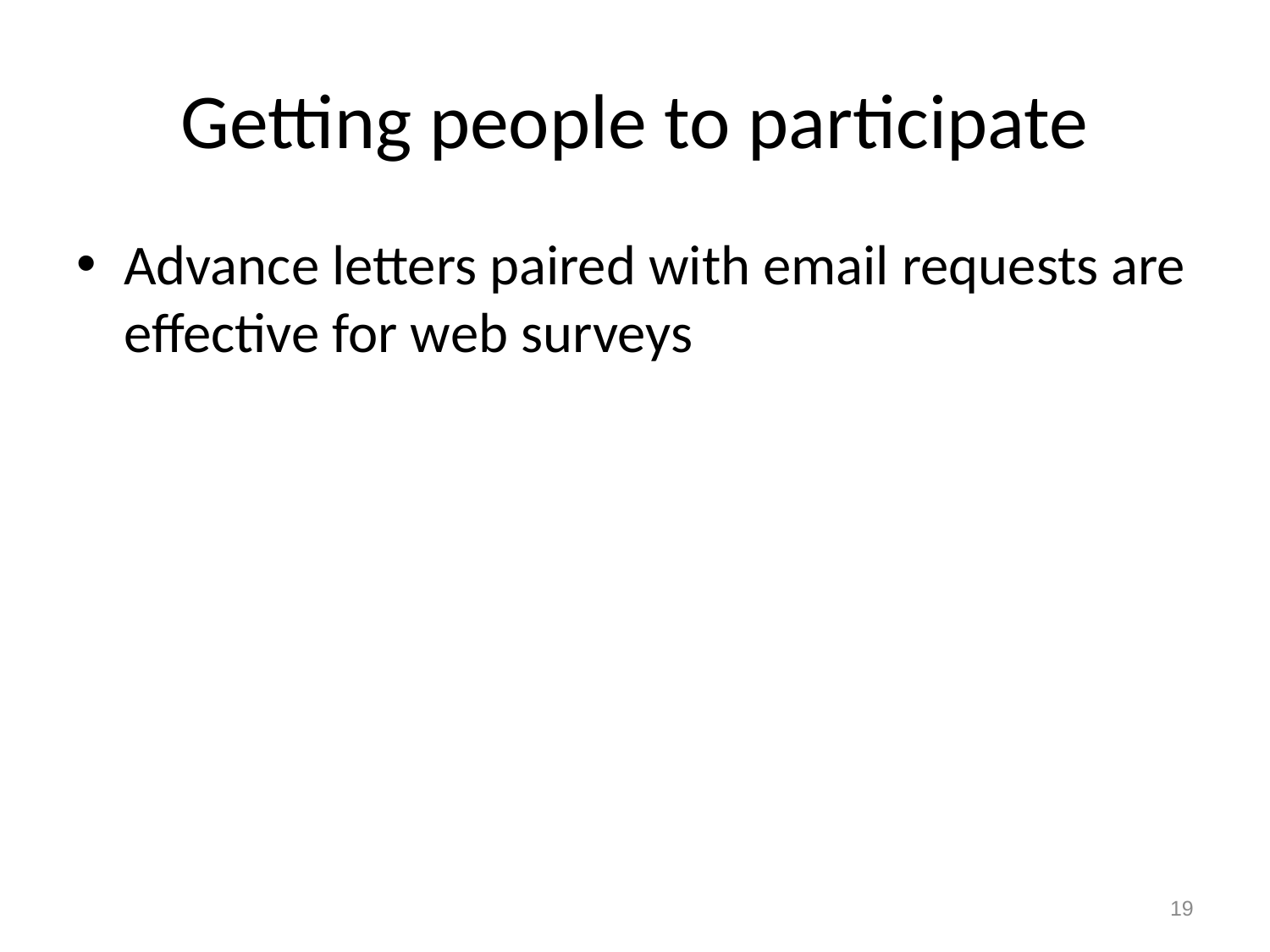

# Getting people to participate
Advance letters paired with email requests are effective for web surveys
19

## Slide 20
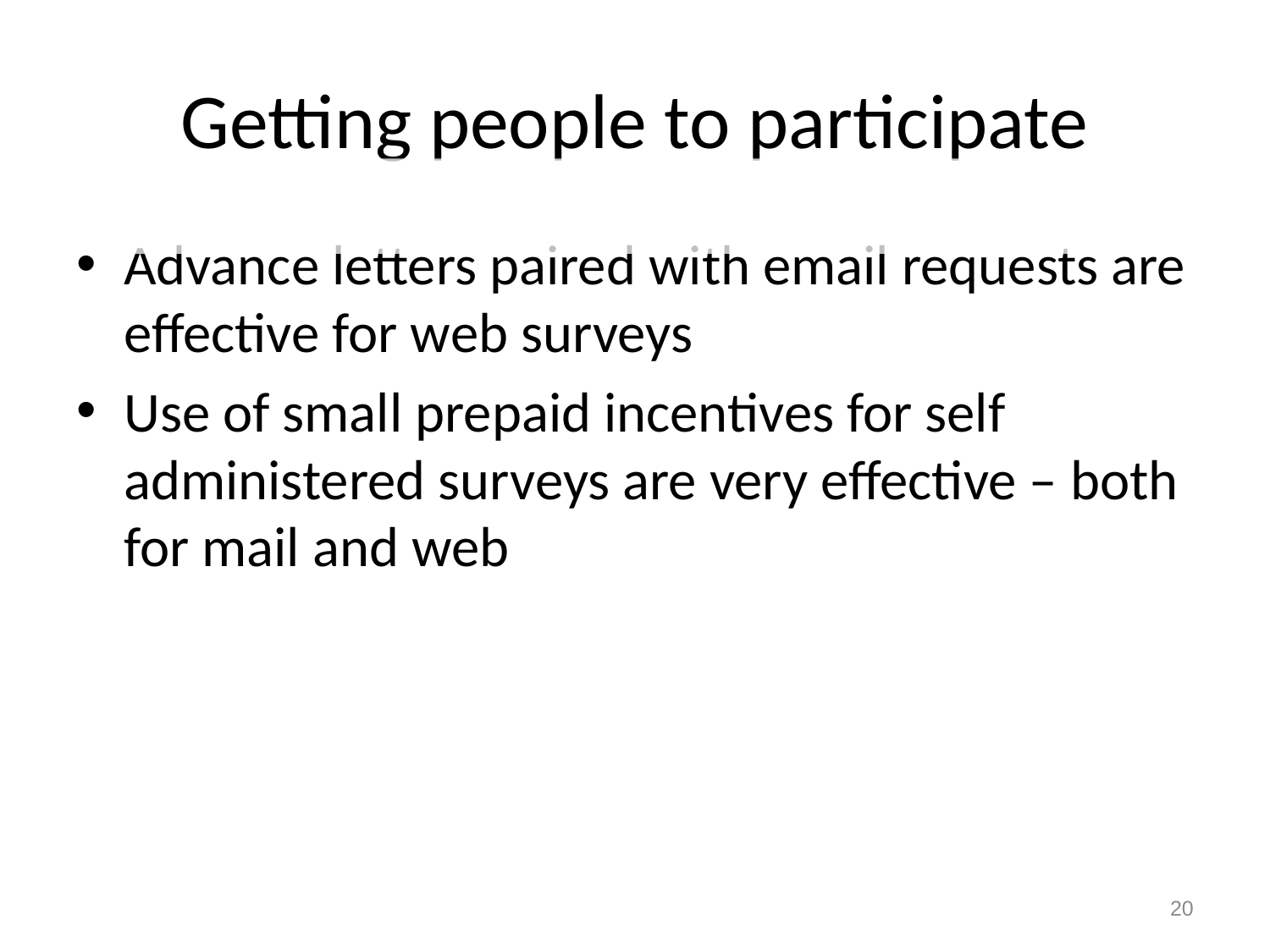

# Getting people to participate
Advance letters paired with email requests are effective for web surveys
Use of small prepaid incentives for self administered surveys are very effective – both for mail and web
20

## Slide 21
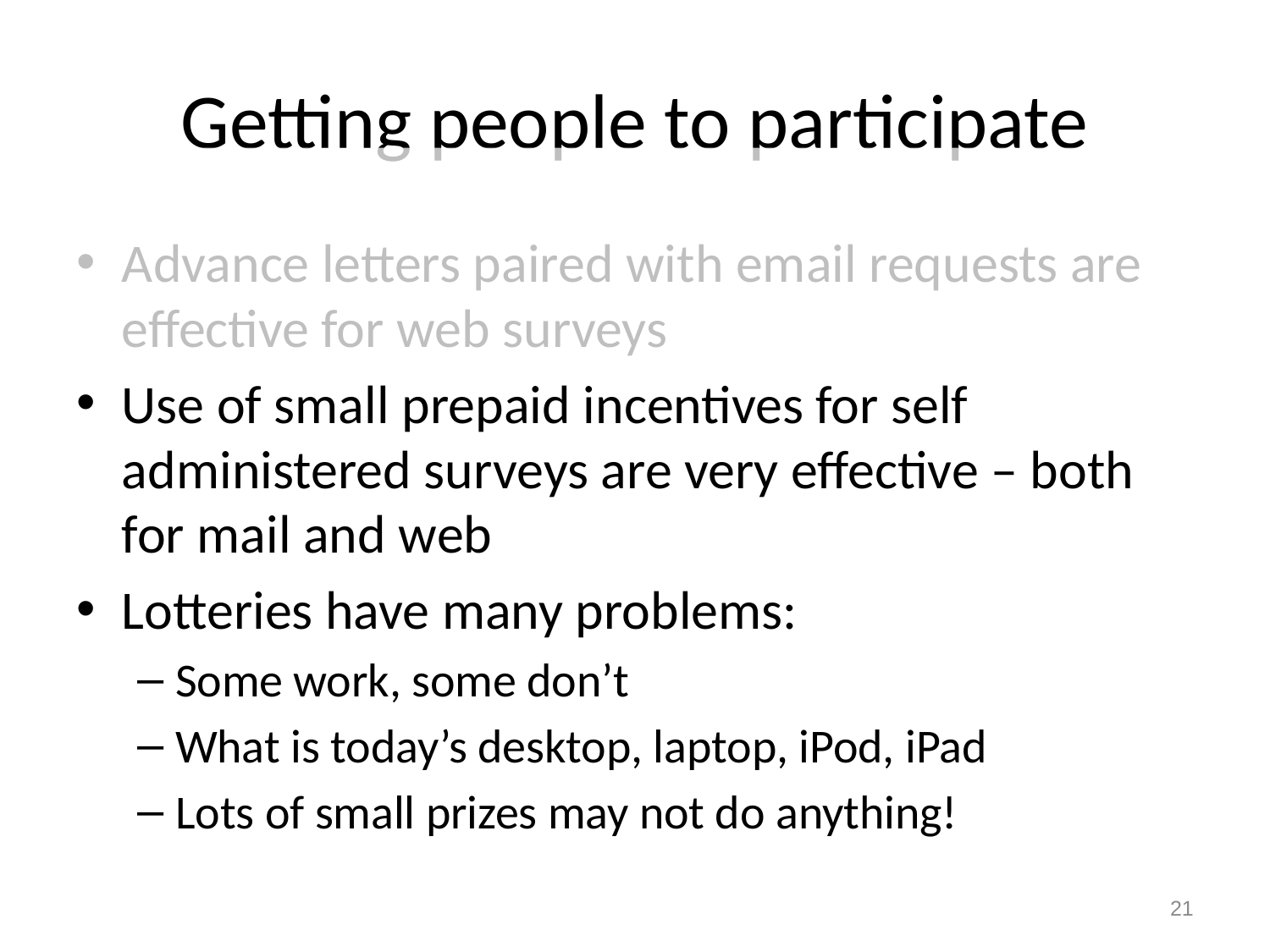

# Getting people to participate
Advance letters paired with email requests are effective for web surveys
Use of small prepaid incentives for self administered surveys are very effective – both for mail and web
Lotteries have many problems:
Some work, some don’t
What is today’s desktop, laptop, iPod, iPad
Lots of small prizes may not do anything!
21

## Slide 22
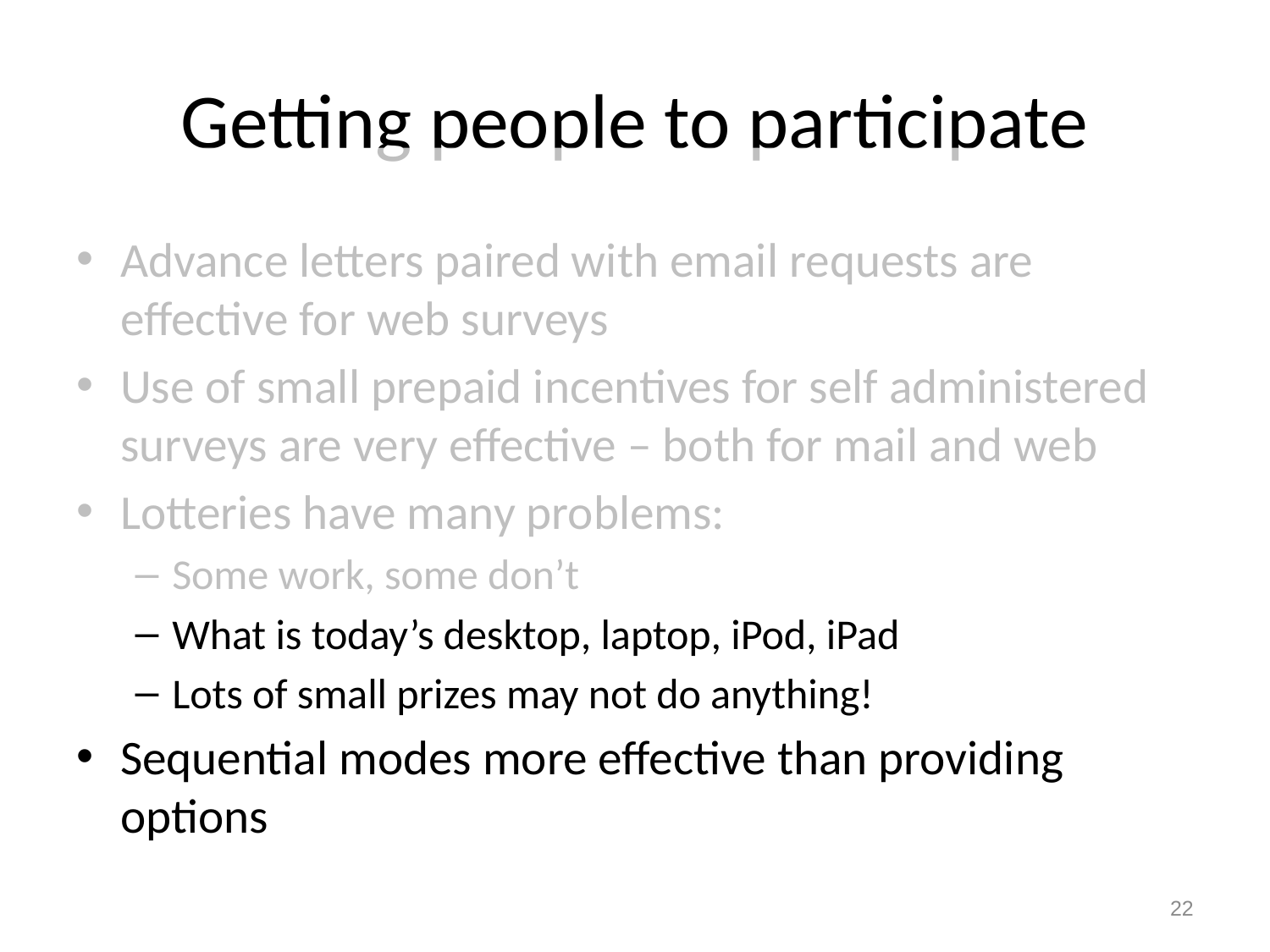

# Getting people to participate
Advance letters paired with email requests are effective for web surveys
Use of small prepaid incentives for self administered surveys are very effective – both for mail and web
Lotteries have many problems:
Some work, some don’t
What is today’s desktop, laptop, iPod, iPad
Lots of small prizes may not do anything!
Sequential modes more effective than providing options
22

## Slide 23
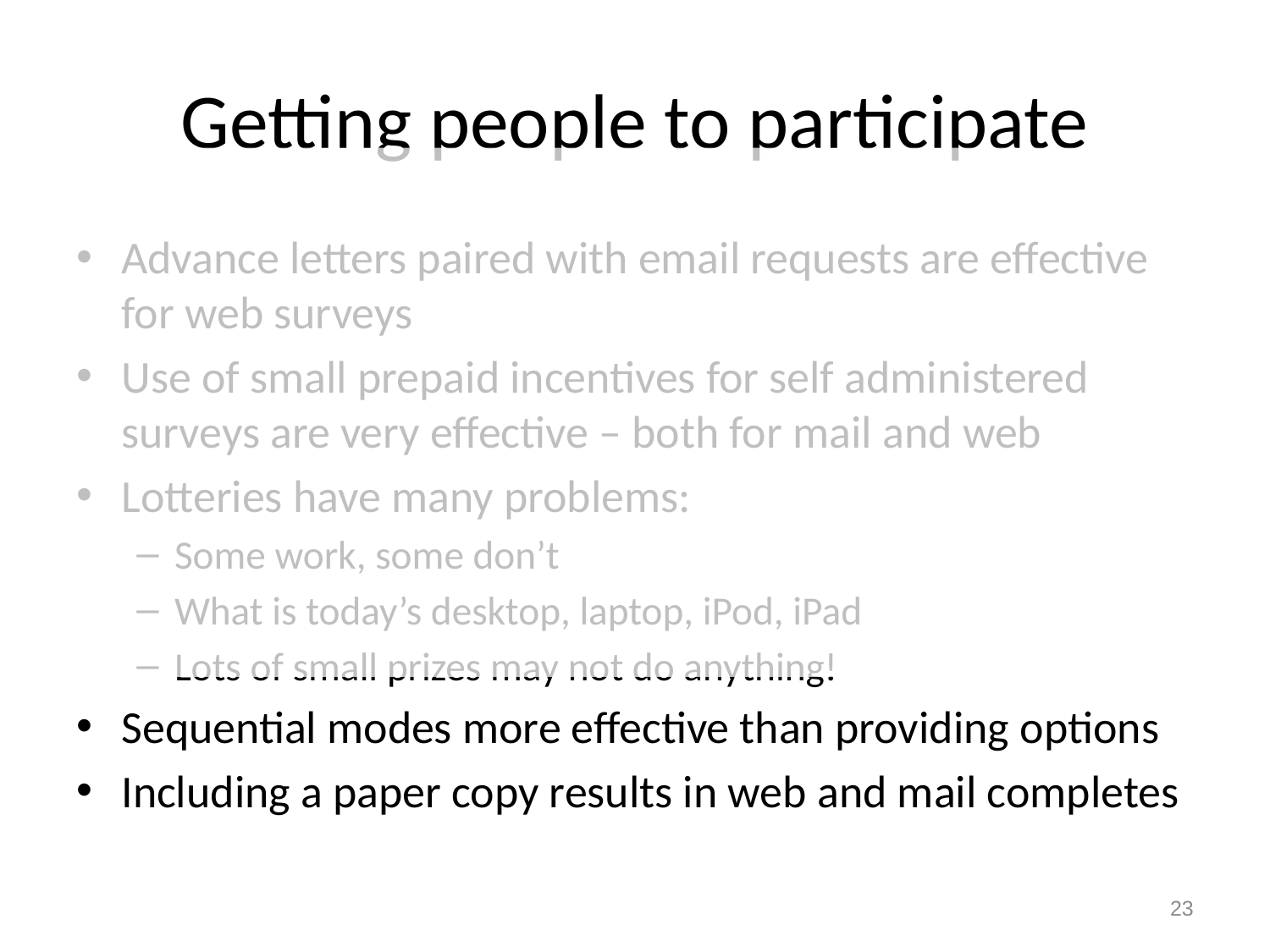

# Getting people to participate
Advance letters paired with email requests are effective for web surveys
Use of small prepaid incentives for self administered surveys are very effective – both for mail and web
Lotteries have many problems:
Some work, some don’t
What is today’s desktop, laptop, iPod, iPad
Lots of small prizes may not do anything!
Sequential modes more effective than providing options
Including a paper copy results in web and mail completes
23

## Slide 24
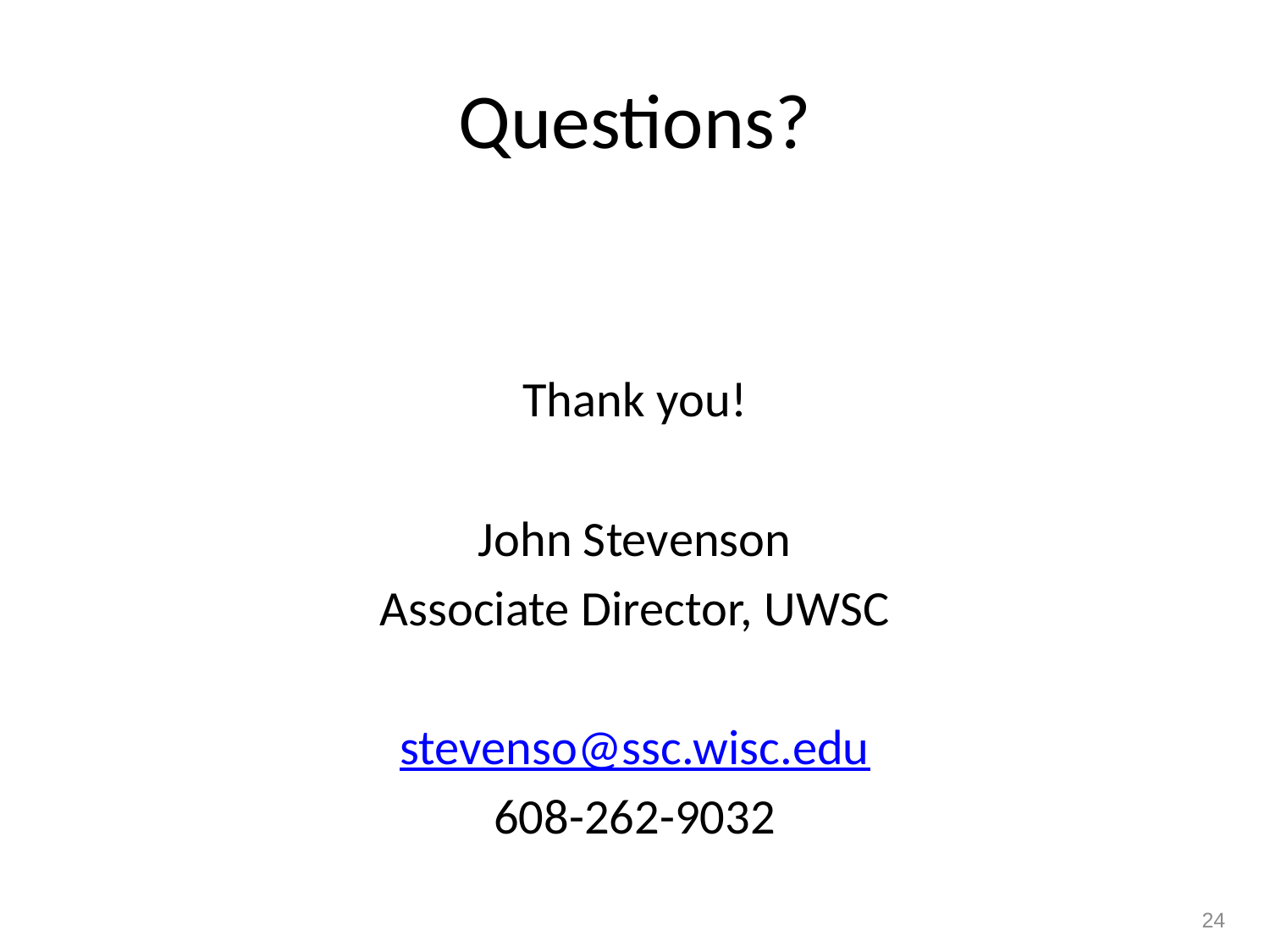

# Questions?
Thank you!
John Stevenson
Associate Director, UWSC
stevenso@ssc.wisc.edu
608-262-9032
24
