# Supplementary material for: A Quality Improvement Curriculum for Psychiatry Residents
Source: MedEdPORTAL. 2020 Jan 24;16:10870. doi: 10.15766/mep_2374-8265.10870 (PMC7012317; doi:10.15766/mep_2374-8265.10870)
Supplement: Supplementary file 1 — A. QI Didactic Seminars.doc B. Introduction to the QI Rotation Slides.ppt C. Essential QI Toolbag Slides.ppt D. Patient Safety Slides.ppt E. Principles of Survey Design Slides.pptx F. CBC and PIP Modules Slides.pptx G. Involving Stakeholders Slides.ppt H. QIKAT for Psychiatry.doc I. QI Workbook.doc J. QI Final Presentation Guidelines.doc K. A3 QI Poster Template 11x17.pptx L. QI Supervisor Evaluation of Resident.docx M. QI Director Evaluation of Resident.pdf N. QI Facts of the Week Sample.docx [file mep-16-10870-s001.zip › F. CBC and PIP Modules Slides.pptx]

## Slide 1
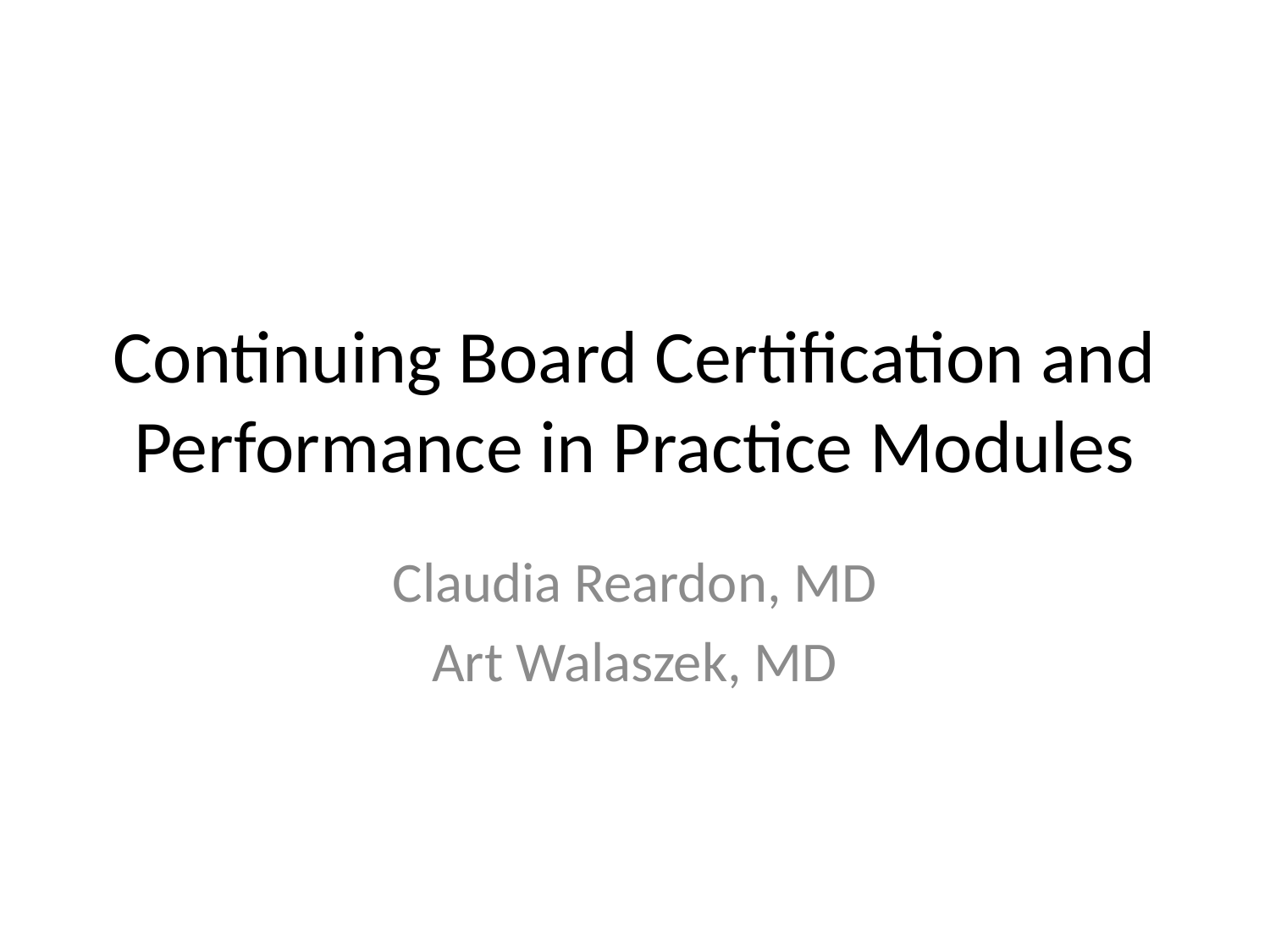

# Continuing Board Certification and Performance in Practice Modules
Claudia Reardon, MD
Art Walaszek, MD

## Slide 2
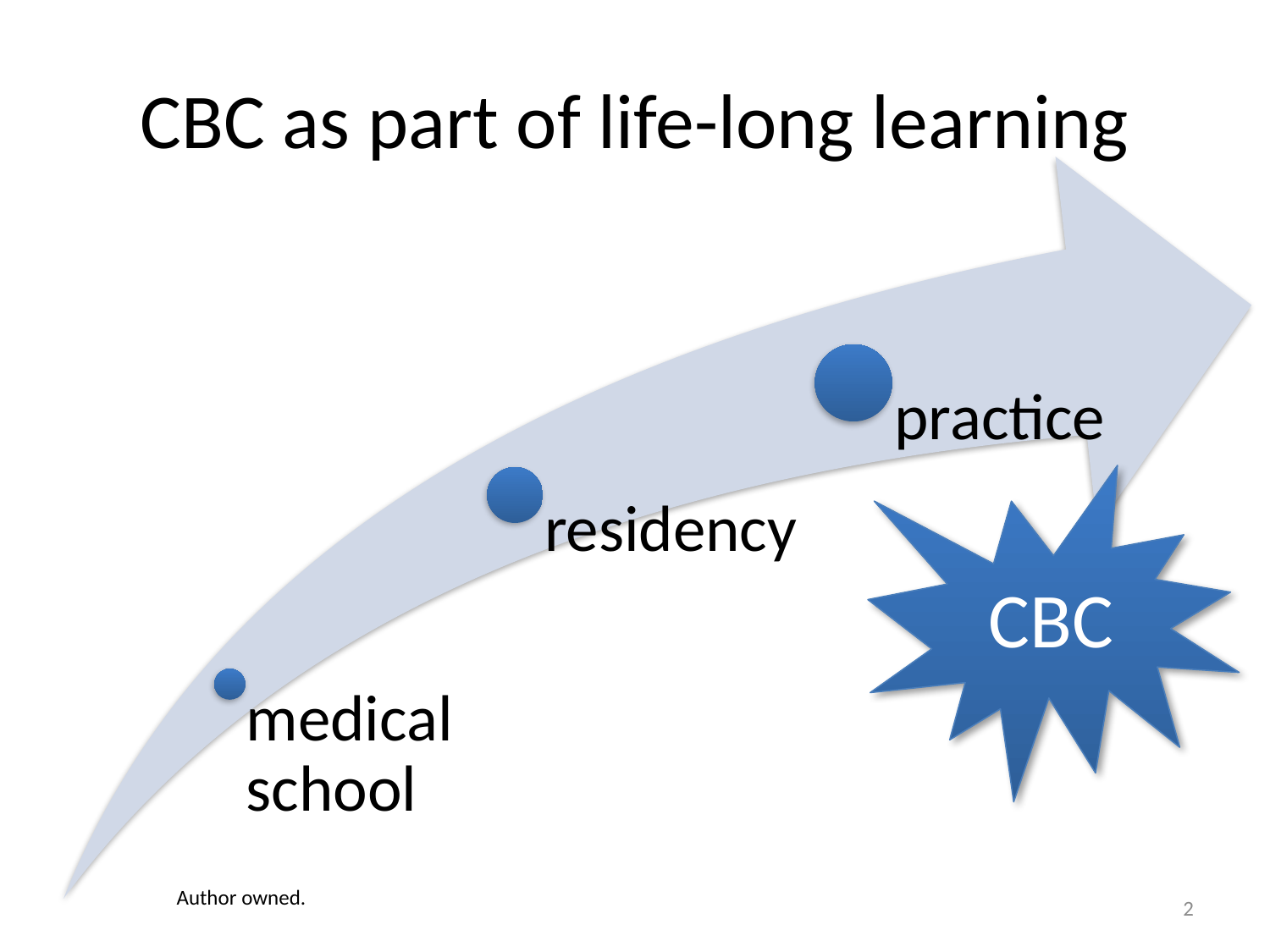

# CBC as part of life-long learning
CBC
Author owned.
2

## Slide 3
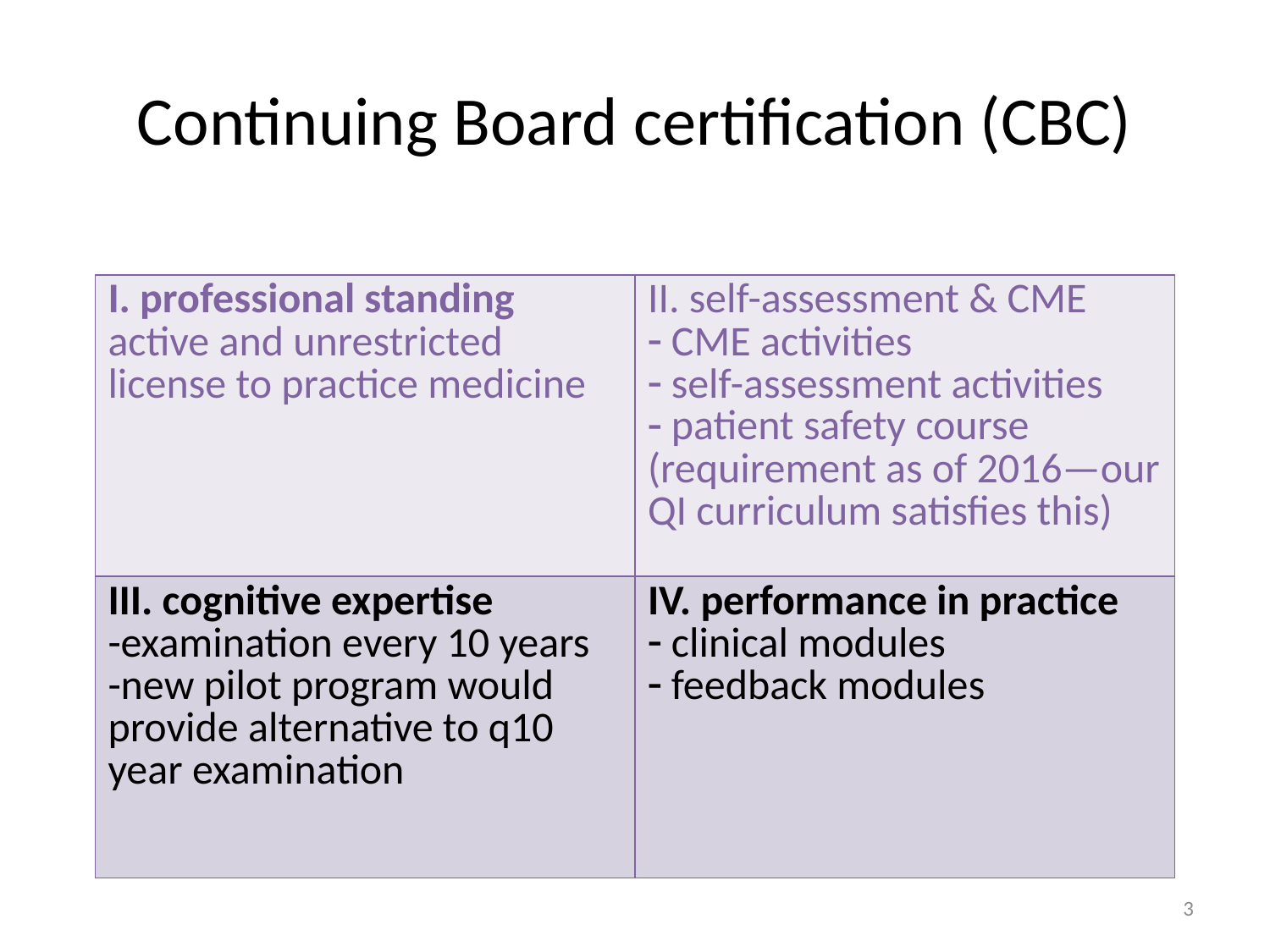

Continuing Board certification (CBC)
| I. professional standing active and unrestricted license to practice medicine | II. self-assessment & CME CME activities self-assessment activities patient safety course (requirement as of 2016—our QI curriculum satisfies this) |
| --- | --- |
| III. cognitive expertise -examination every 10 years -new pilot program would provide alternative to q10 year examination | IV. performance in practice clinical modules feedback modules |
3

## Slide 4
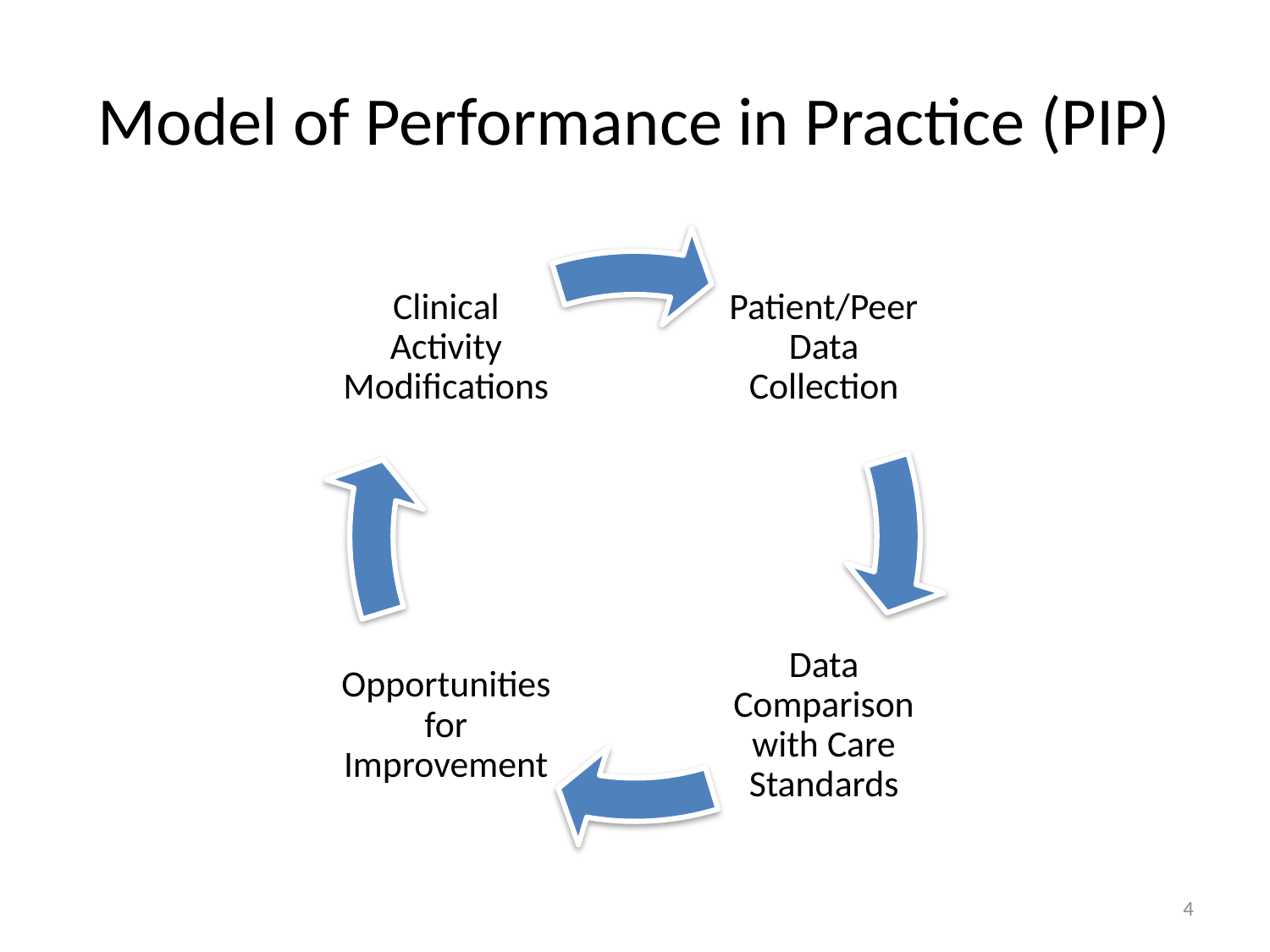

# Model of Performance in Practice (PIP)
4

## Slide 5
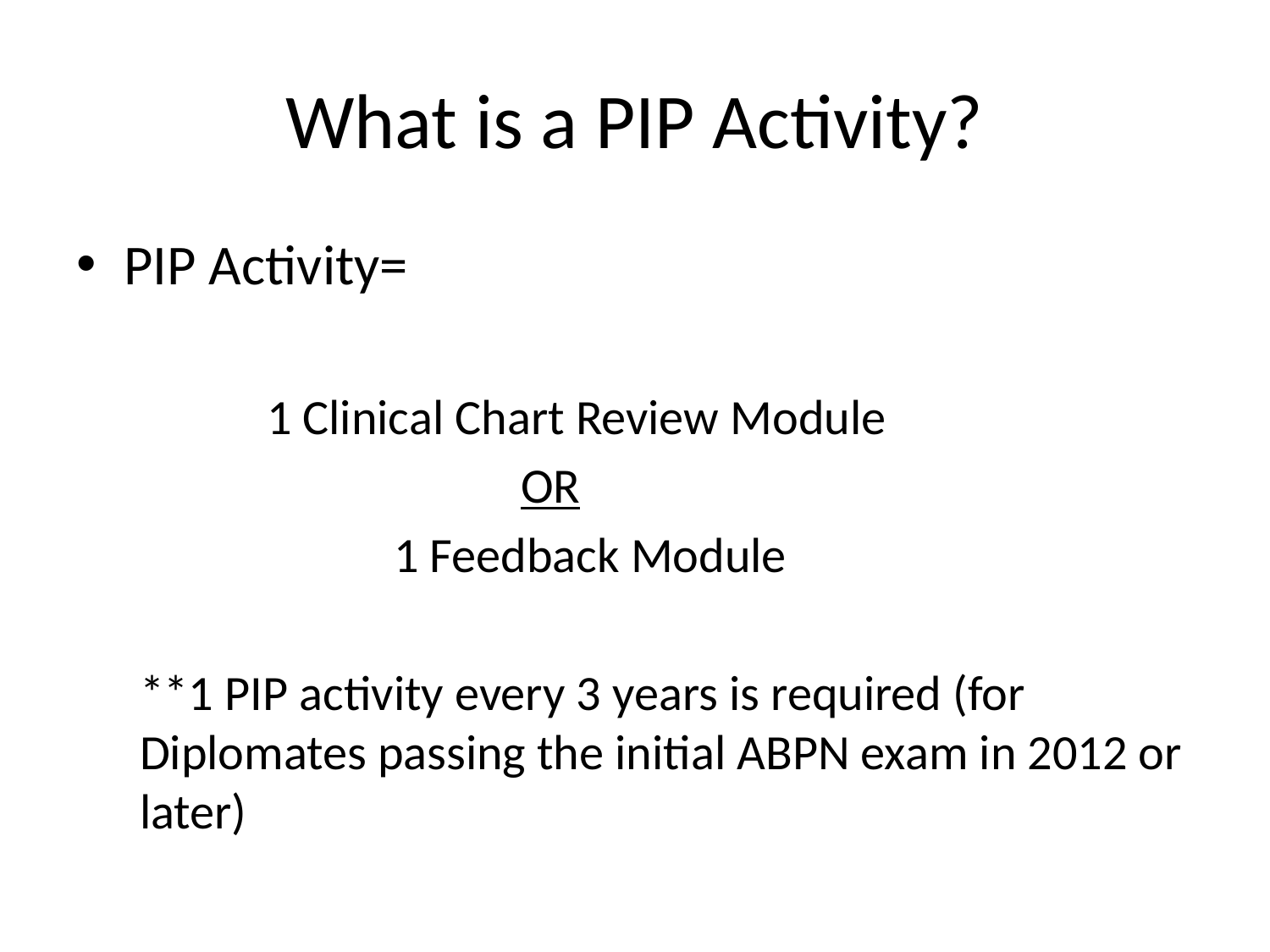

# What is a PIP Activity?
PIP Activity=
	1 Clinical Chart Review Module
			OR
		1 Feedback Module
**1 PIP activity every 3 years is required (for Diplomates passing the initial ABPN exam in 2012 or later)

## Slide 6
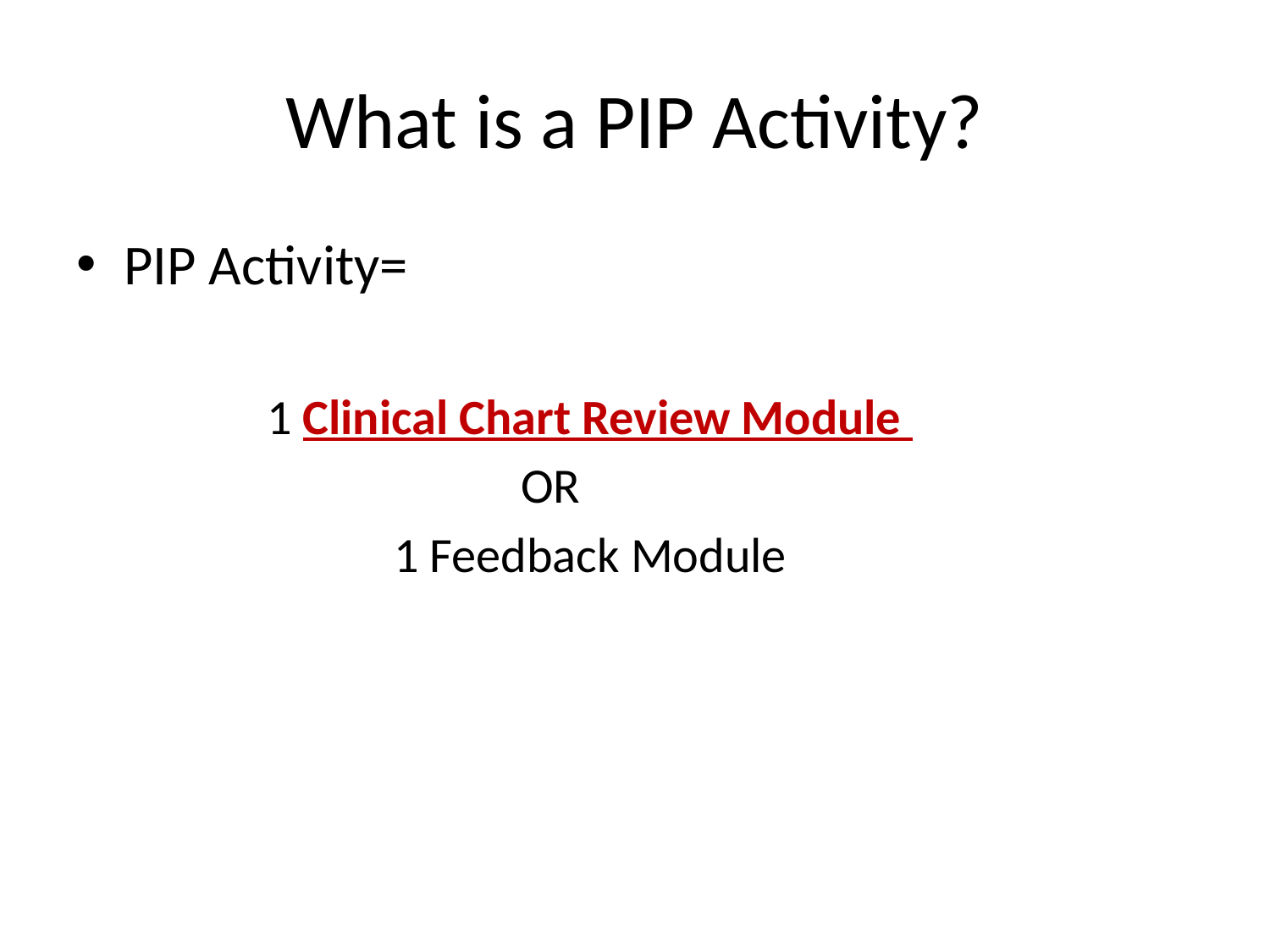

# What is a PIP Activity?
PIP Activity=
	1 Clinical Chart Review Module
			OR
		1 Feedback Module

## Slide 7
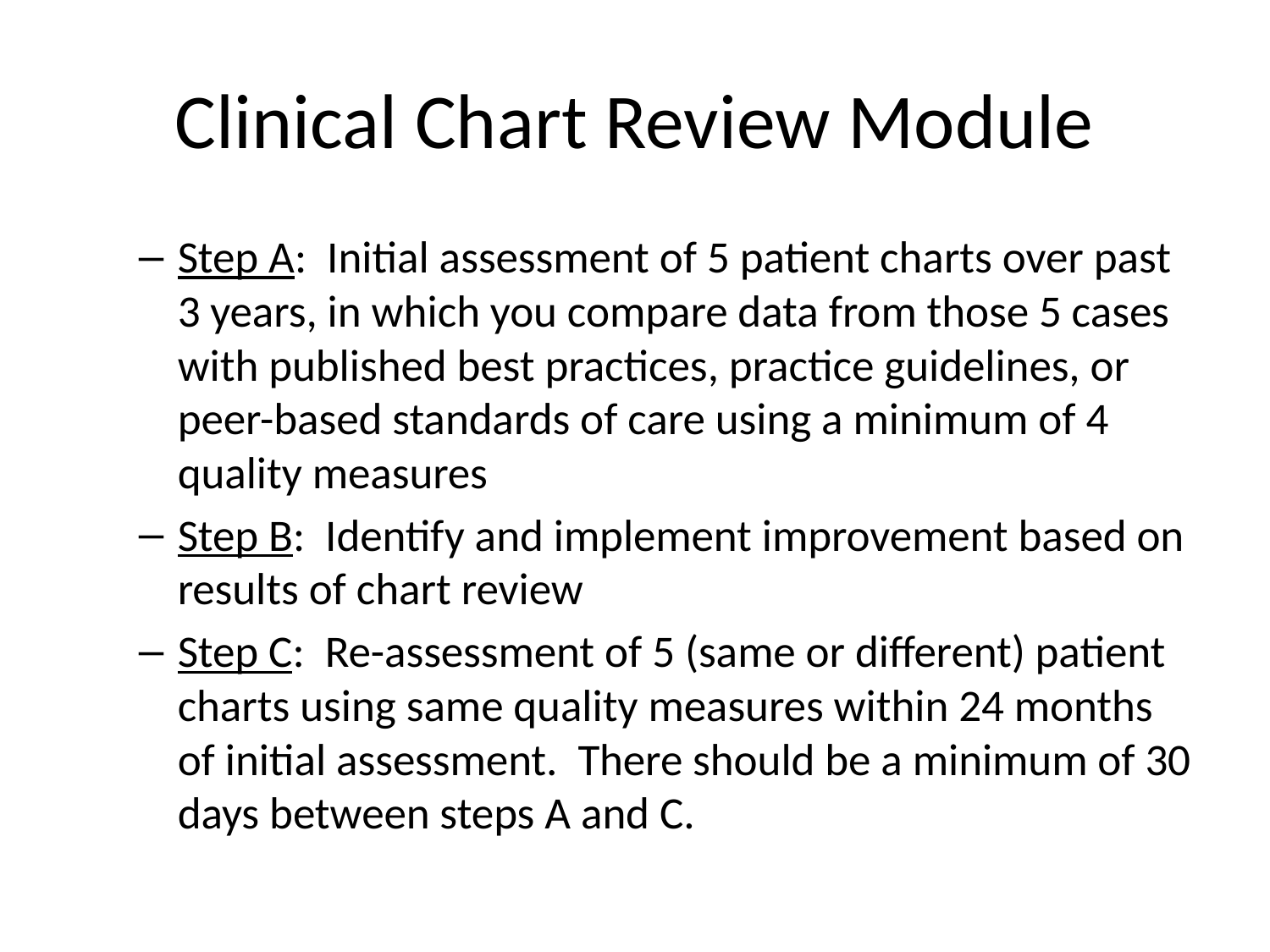

# Clinical Chart Review Module
Step A: Initial assessment of 5 patient charts over past 3 years, in which you compare data from those 5 cases with published best practices, practice guidelines, or peer-based standards of care using a minimum of 4 quality measures
Step B: Identify and implement improvement based on results of chart review
Step C: Re-assessment of 5 (same or different) patient charts using same quality measures within 24 months of initial assessment. There should be a minimum of 30 days between steps A and C.

## Slide 8
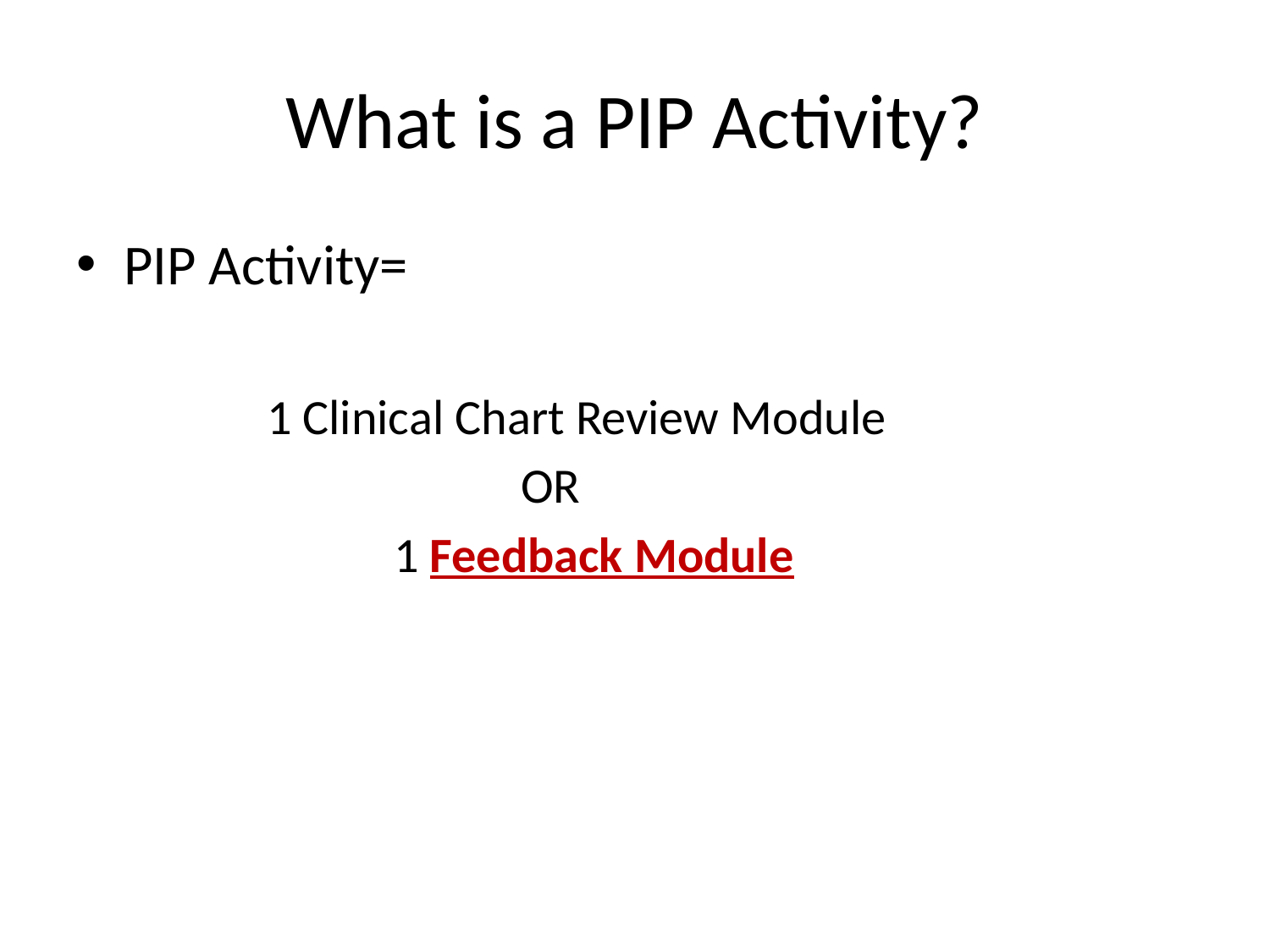

# What is a PIP Activity?
PIP Activity=
	1 Clinical Chart Review Module
			OR
		1 Feedback Module

## Slide 9
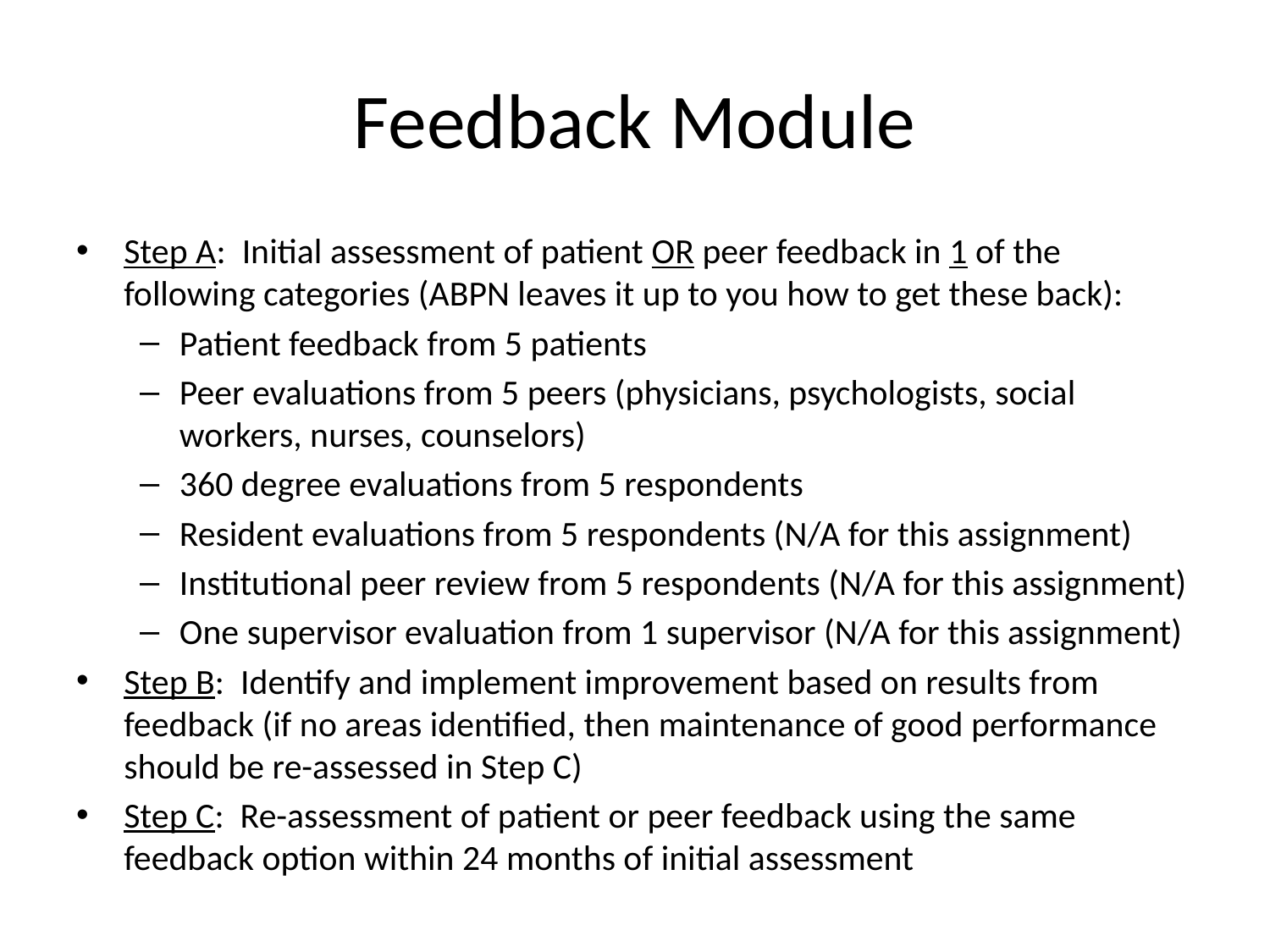

# Feedback Module
Step A: Initial assessment of patient OR peer feedback in 1 of the following categories (ABPN leaves it up to you how to get these back):
Patient feedback from 5 patients
Peer evaluations from 5 peers (physicians, psychologists, social workers, nurses, counselors)
360 degree evaluations from 5 respondents
Resident evaluations from 5 respondents (N/A for this assignment)
Institutional peer review from 5 respondents (N/A for this assignment)
One supervisor evaluation from 1 supervisor (N/A for this assignment)
Step B: Identify and implement improvement based on results from feedback (if no areas identified, then maintenance of good performance should be re-assessed in Step C)
Step C: Re-assessment of patient or peer feedback using the same feedback option within 24 months of initial assessment

## Slide 10
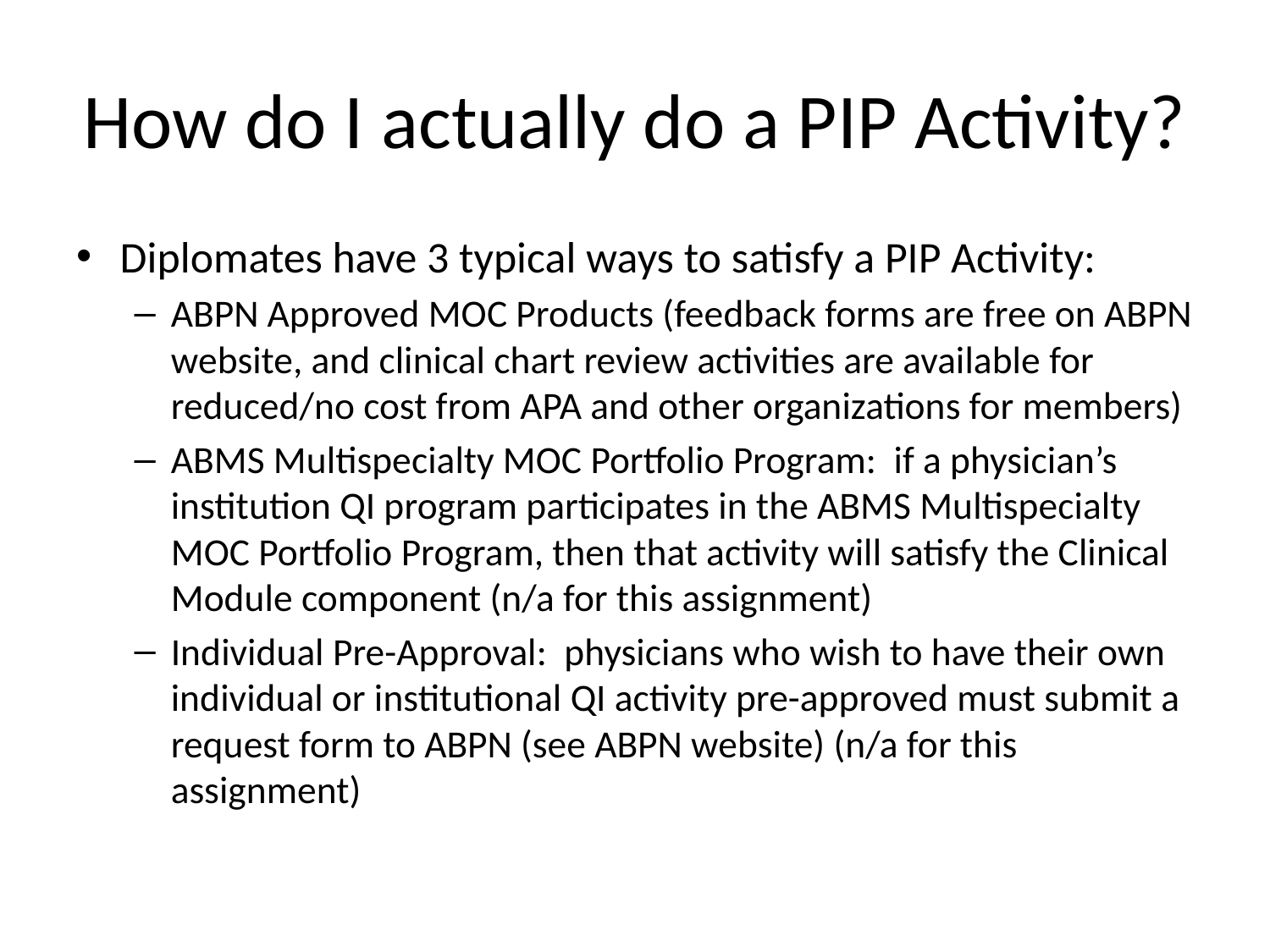

# How do I actually do a PIP Activity?
Diplomates have 3 typical ways to satisfy a PIP Activity:
ABPN Approved MOC Products (feedback forms are free on ABPN website, and clinical chart review activities are available for reduced/no cost from APA and other organizations for members)
ABMS Multispecialty MOC Portfolio Program: if a physician’s institution QI program participates in the ABMS Multispecialty MOC Portfolio Program, then that activity will satisfy the Clinical Module component (n/a for this assignment)
Individual Pre-Approval: physicians who wish to have their own individual or institutional QI activity pre-approved must submit a request form to ABPN (see ABPN website) (n/a for this assignment)

## Slide 11
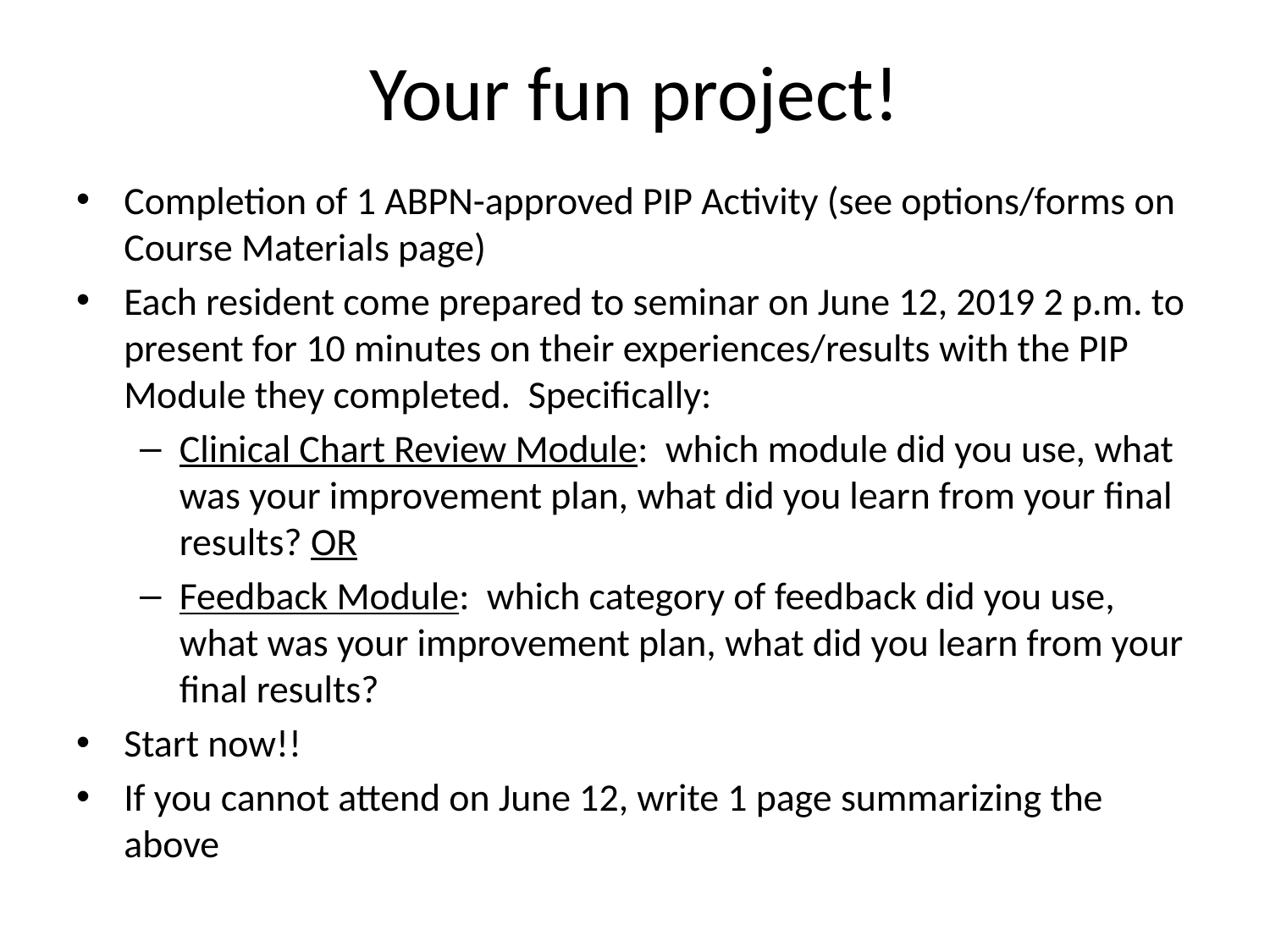

# Your fun project!
Completion of 1 ABPN-approved PIP Activity (see options/forms on Course Materials page)
Each resident come prepared to seminar on June 12, 2019 2 p.m. to present for 10 minutes on their experiences/results with the PIP Module they completed. Specifically:
Clinical Chart Review Module: which module did you use, what was your improvement plan, what did you learn from your final results? OR
Feedback Module: which category of feedback did you use, what was your improvement plan, what did you learn from your final results?
Start now!!
If you cannot attend on June 12, write 1 page summarizing the above
