# Supplementary material for: A Quality Improvement Curriculum for Psychiatry Residents
Source: MedEdPORTAL. 2020 Jan 24;16:10870. doi: 10.15766/mep_2374-8265.10870 (PMC7012317; doi:10.15766/mep_2374-8265.10870)
Supplement: Supplementary file 1 — A. QI Didactic Seminars.doc B. Introduction to the QI Rotation Slides.ppt C. Essential QI Toolbag Slides.ppt D. Patient Safety Slides.ppt E. Principles of Survey Design Slides.pptx F. CBC and PIP Modules Slides.pptx G. Involving Stakeholders Slides.ppt H. QIKAT for Psychiatry.doc I. QI Workbook.doc J. QI Final Presentation Guidelines.doc K. A3 QI Poster Template 11x17.pptx L. QI Supervisor Evaluation of Resident.docx M. QI Director Evaluation of Resident.pdf N. QI Facts of the Week Sample.docx [file mep-16-10870-s001.zip › K. A3 QI Poster Template 11x17.pptx]

## Slide 1
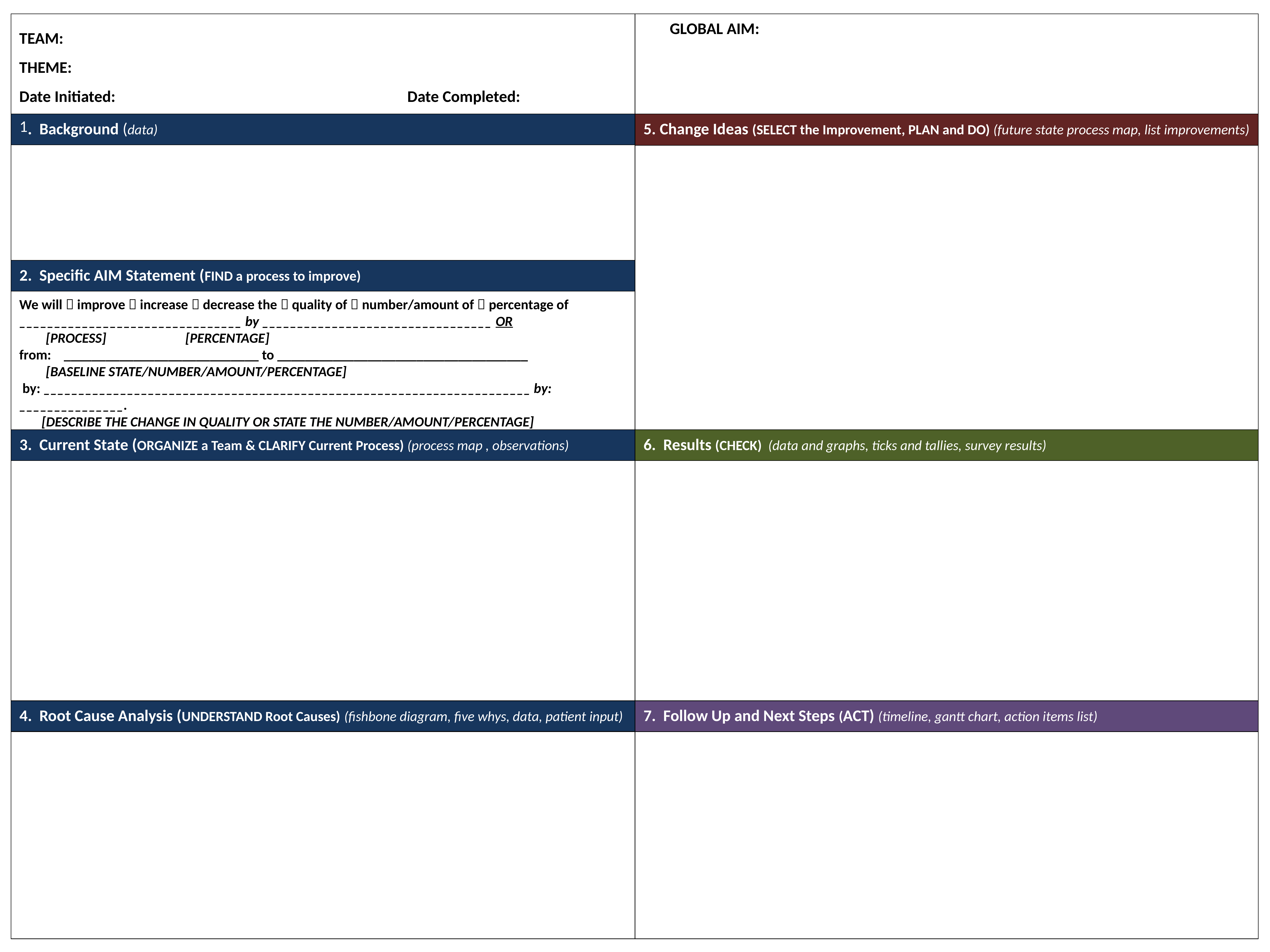

TEAM:
THEME:
Date Initiated: Date Completed:
	GLOBAL AIM:
. Background (data)
5. Change Ideas (SELECT the Improvement, PLAN and DO) (future state process map, list improvements)
2. Specific AIM Statement (FIND a process to improve)
We will  improve  increase  decrease the  quality of  number/amount of  percentage of ________________________________ by _________________________________ OR
	[PROCESS]	 [PERCENTAGE]
from: ____________________________ to ____________________________________
	[BASELINE STATE/NUMBER/AMOUNT/PERCENTAGE]
 by: ______________________________________________________________________ by: _______________.
 [DESCRIBE THE CHANGE IN QUALITY OR STATE THE NUMBER/AMOUNT/PERCENTAGE]
6. Results (CHECK) (data and graphs, ticks and tallies, survey results)
3. Current State (ORGANIZE a Team & CLARIFY Current Process) (process map , observations)
4. Root Cause Analysis (UNDERSTAND Root Causes) (fishbone diagram, five whys, data, patient input)
7. Follow Up and Next Steps (ACT) (timeline, gantt chart, action items list)
