# Supplementary material for: A Quality Improvement Curriculum for Psychiatry Residents
Source: MedEdPORTAL. 2020 Jan 24;16:10870. doi: 10.15766/mep_2374-8265.10870 (PMC7012317; doi:10.15766/mep_2374-8265.10870)
Supplement: Supplementary file 1 — A. QI Didactic Seminars.doc B. Introduction to the QI Rotation Slides.ppt C. Essential QI Toolbag Slides.ppt D. Patient Safety Slides.ppt E. Principles of Survey Design Slides.pptx F. CBC and PIP Modules Slides.pptx G. Involving Stakeholders Slides.ppt H. QIKAT for Psychiatry.doc I. QI Workbook.doc J. QI Final Presentation Guidelines.doc K. A3 QI Poster Template 11x17.pptx L. QI Supervisor Evaluation of Resident.docx M. QI Director Evaluation of Resident.pdf N. QI Facts of the Week Sample.docx [file mep-16-10870-s001.zip › L. QI Supervisor Evaluation of Resident.docx]

**QI FACULTY SUPERVISOR EVALUATION OF RESIDENT**

| **Practice-Based Learning and Improvement** | | | | | |  |
| --- | --- | --- | --- | --- | --- | --- |
| **Specific quality improvement project** |   **0** |   **1** |   **2** |   **3** |   **4** | **Not observed** |
|  | Cannot identify potential gaps in quality of care and system-level inefficiencies when brainstorming QI project ideas  Does not appreciate that others (clinicians, administrators, support staff, etc) may be impacted by a QI project  Does not contribute at all to a QI project | Recognizes potential gaps in quality of care and system-level inefficiencies and comes up with a QI project idea related to that  Recognizes that others (clinicians, administrators, support staff, etc) may be impacted by a QI project  Contributes peripherally to a QI project but frequently does not show up to planned meetings or QI supervision or follow through on assigned tasks  PBLI2-1.1/A | Recognizes potential gaps in quality of care and system-level inefficiencies, and then narrows those problems down to a specific and achievable AIM for a quality improvement project (AIM=a goal for improvement in a system of care that defines ‘how good’, ‘by when’, and ‘for whom’)  Recognizes that others (clinicians, administrators, support staff, etc) may be impacted by a QI project and informs those parties about the QI project  Contributes to a QI project but sometimes does not show up to planned meetings or QI supervision or sometimes does not follow through on assigned tasks  PBLI2-2.1/A | Not only recognizes that others may be impacted by a QI project, but also actually involves those appropriate stakeholders in DESIGN of the QI project  Contributes to a QI project though does not demonstrate substantial ownership and accountability for the project or measure the relevant outcomes  PBLI2-3.1/A | Substantially contributes to a supervised project to address a specific quality deficit in clinical practice and measures relevant outcomes  PBLI2-4.1/A |  |
| **Additional comments:** | | | | | |  |
