# Supplementary material for: A Quality Improvement Curriculum for Psychiatry Residents
Source: MedEdPORTAL. 2020 Jan 24;16:10870. doi: 10.15766/mep_2374-8265.10870 (PMC7012317; doi:10.15766/mep_2374-8265.10870)
Supplement: Supplementary file 1 — A. QI Didactic Seminars.doc B. Introduction to the QI Rotation Slides.ppt C. Essential QI Toolbag Slides.ppt D. Patient Safety Slides.ppt E. Principles of Survey Design Slides.pptx F. CBC and PIP Modules Slides.pptx G. Involving Stakeholders Slides.ppt H. QIKAT for Psychiatry.doc I. QI Workbook.doc J. QI Final Presentation Guidelines.doc K. A3 QI Poster Template 11x17.pptx L. QI Supervisor Evaluation of Resident.docx M. QI Director Evaluation of Resident.pdf N. QI Facts of the Week Sample.docx [file mep-16-10870-s001.zip › M. QI Director Evaluation of Resident.pdf]

QI ROTATION DIRECTOR EVALUATION OF QI PROJECT PRESENTATION AND QI/PATIENT SAFETY PARTICIPATION

Practice-Based Learning and Improvement						
**Quality improvement didactic knowledge (as evidenced during final QI project presentation)**	☐ **0**	☐ **1**	☐ **2**	☐ **3**	☐ **4**	☐ **Not observed**
	Does not discuss quality gaps and problems with psychiatric care delivery	Discusses quality gaps and problems with psychiatric care delivery<br><br>PBLI2-1.1/B	Outlines factors and causal chains contributing to quality gaps<br><br>PBLI2-2.2/B	Lists details of multiple barriers encountered in QI project AND strategies for getting stakeholder buy-in from those affected by the project<br><br>PBLI2-3.2/B	Everything at lower levels, and also describes details of methods for implementation (including at least 1 discrete QI tool such as a PDCA cycle) and evaluation (including comparison of baseline and final data) of a clinical QI project<br><br>PBLI2-4.2/B	
	As described, project has NO relationship to a quality initiative of WisPIC, UWHC, VA, APA, JCAHO, or other entity to which we are accountable.		As described, project has SOME relationship to a quality initiative of WisPIC, UWHC, VA, APA, JCAHO, or other entity to which we are accountable.		As described, project has DIRECT AND COMPELLING relationship to a quality initiative of WisPIC, UWHC, VA, APA, JCAHO, or other entity to which we are accountable	
**Development as a teacher**	Fails to participate in the required	-	-	-	Gives formal QI project presentations	

	formal QI project presentations				as part of midpoint project presentations, to UW Psychiatry QI Committee, and to Department of Psychiatry. The latter is presented in Grand Rounds format and includes an A3 poster.<br><br>PBLI3-4.1/A	
**Sustainability of QI project (as evidenced during final QI project presentation)**	No mention made of sustainability of the QI project	-	Discussed the issue of sustainability of the QI project	Presented some evidence that shows the resident team attempted to make their QI intervention sustainable even after they complete their QI rotation	Presented strong evidence that the QI intervention is extremely likely to be sustainable even after the resident team completes their QI rotation	
**Additional comments:**						

Systems-Based Practice						
**Regulatory and educational activities related to patient safety**	☐ **0**	☐ **1**	☐ **2**	☐ **3**	☐ **4**	☐ **Not observed**
		Has not demonstrated any problems following institutional safety policies<br><br>SBP1-1.3/C	Activity participates in conferences focusing on systems-based errors in patient care<br><br>SBP1-2.3/C	-	Activity participates in conferences focusing on systems-based errors in patient care AND also develops content for and/or helps facilitate a morbidity and mortality conference<br><br>SBP1-4.2/C	
**Medical errors and improvement activities (as evidenced by participation in Patient Safety and Root Cause Analysis seminars, or if seminars missed, by seeking out and completing makeup activities from QI rotation director)**		Can differentiate among medical errors, near misses, and sentinel events<br><br>Can recognize failure in teamwork and communication as leading cause of preventable patient harm<br><br>SBP1-1.1/A	Everything at level 1, and also can describe the common system causes for errors<br><br>SBP1-2.1/A	Everything at levels 1 and 2, and also can describe systems and procedures that promote patient safety<br><br>SBP1-3.1/A	Everything at levels 1-3, and also participates in a root-cause analysis activity (part of QI seminar)<br><br>SBP1-4.1/A	

Comments:	

Professionalism						
**Professional behavior and participation in professional community**	**0**	**1**	**2**	**3**	**4**	**Not observed**
	Does not complete a Performance in Practice module	Partially completes a Performance in Practice module		-	Prepares for obtaining and maintaining board certification by completing a Performance in Practice module and presenting results to colleagues<br><br>PROF2-4.3/B	
**Comments:**						
