# Supplementary material for: A Quality Improvement Curriculum for Psychiatry Residents
Source: MedEdPORTAL. 2020 Jan 24;16:10870. doi: 10.15766/mep_2374-8265.10870 (PMC7012317; doi:10.15766/mep_2374-8265.10870)
Supplement: Supplementary file 1 — A. QI Didactic Seminars.doc B. Introduction to the QI Rotation Slides.ppt C. Essential QI Toolbag Slides.ppt D. Patient Safety Slides.ppt E. Principles of Survey Design Slides.pptx F. CBC and PIP Modules Slides.pptx G. Involving Stakeholders Slides.ppt H. QIKAT for Psychiatry.doc I. QI Workbook.doc J. QI Final Presentation Guidelines.doc K. A3 QI Poster Template 11x17.pptx L. QI Supervisor Evaluation of Resident.docx M. QI Director Evaluation of Resident.pdf N. QI Facts of the Week Sample.docx [file mep-16-10870-s001.zip › N. QI Facts of the Week Sample.docx]

**Sample QI Facts of the Week**

**Note that these are provided as examples of the kinds of Facts that might be authored for residents and faculty in a Department of Psychiatry. The nature of these Facts is that they often provide very recent quality improvement information that is often geography-specific. Thus, there is no substitute for faculty members at one’s own institution writing their own updated Facts on a regular basis. Additionally, weblinks change with time and may no longer be active (and thus, as per MedEd Portal policy, all weblinks are noted as “weblink optional reading”).*

Dear Residents,

**This week’s *Fact*:  QI as a great opportunity for scholarly work**

Many physicians, including residents, feel that they don’t have the background or inclination to publish or present work they do.  I am here to say that it isn’t all that hard, and QI projects (which all our residents need to complete anyways!) are a great opportunity to do just that.  Whether it is a PGY3 9-month-long, detailed QI project, or a PGY1 QI project that you’re fitting into all your "free time" (that’s you, Erin Cheers!), these are great opportunities for getting a taste of what it is like to disseminate your work.  The fact is that if you are doing all the work of a QI project anyways, you might as well think about sharing the experience with others.  In general, as a specialty, psychiatry has been way behind the curve in QI work, and so any little thing we can contribute potentially represents a meaningful contribution to the field.

Here are some examples of where/how you can disseminate your QI projects:

1)  A poster presentation of your project at a national meeting dedicated to psychiatric education.  Examples include the American Association of Directors of Psychiatric Residency Education (AADPRT) or the Association for Academic Psychiatry (AAP).  In fact, two of our residency alums presented a QI poster at the 2013 meeting of AAP, and they won overall first prize (!!!) out of all poster presenters at the meeting—seriously.

2)  A poster presentation of your project at the forthcoming April 2, 2016 inaugural Wisconsin Medical Society Quality Improvement and Research Forum.   And by the way, huge kudos to our own residents for being one of the Medical Society members who advocated for this forum, and who specifically pointed out to the Society that it would be a great venue for residents across all specialities in our state to present their QI projects!

3)  A poster presentation of your project at UWHC’s Quality and Safety Week.  This year, Quality Week is next week (October 26-29).  Stop over to the HSLC Atrium any time on any of those days to see QI project posters on display (and start to get ideas for your own QI posters in this venue in future years!).

4)  An actual journal article of your project.  Residency alum Fred Langheim, MD, PhD published his residency QI project [here](http://www.ncbi.nlm.nih.gov/pmc/articles/PMC4039094/) (weblink optional reading).  Another of our alums, Elliot Lee, MD, PhD, published his residency QI project [here](http://www.fedprac.com/avaho-home/single-article/using-life-stories-to-connect-veterans-and-providers/abc67a2c40b6030a9e45dfdb20b04923.html) (weblink optional reading).  The journal [Academic Psychiatry](http://www.springer.com/medicine/psychiatry/journal/40596) (weblink optional reading) would be a great venue for this type of paper, but there are many possibilities.

Dear Residents,

**This week’s *Fact*:  Performance Measures**

Many of our colleagues in non-psychiatric medical specialties are very used to being measured on their doctorly performance, and even receiving compensation in accordance with that.  In general, psychiatry has not yet been as measurement-based as other specialties, probably in part because outcomes measures are not as readily available and are not regarded as being as ‘objective’ as they are in some fields.  For example, while primary care docs have hemoglobin A1cs to indicate how their diabetic patients are doing, people have been less comfortable saying that PHQ9s should be used as a simple measure to say how good we are at treating depressed patients.

That said, more and more performance measures are being developed for psychiatry.  In the APA’s Psychiatric News email from just two days ago, there was an article about the APA’s and American Academy of Neurology’s newly-drafted performance measures for dementia.  The article notes that “these measures may influence future quality improvement accountability programs and psychiatric clinician reimbursement”.

You can find this specific draft performance measure, and access to an online comment form to weigh in on the draft, [here](http://tools.aan.com/practice/measures/index.cfm?event=comment:home&project_id=9) (weblink optional reading).  The comment period is open through December 1.  One example of the performance measures being proposed in this new document is the percentage of patients with dementia whose caregivers were provided with education on dementia disease management and health behavior changes AND were referred to additional resources for support in the last 12 months.  The footnote says that “additional resources” include national organizations such as the Alzheimer’s Association, and also local resources, such as community, senior center and religion-based support groups. Art surely does this with every single one of his geriatric patient-family units, but how many of us can say that we do so consistently?

The good news is that physicians are generally being given the opportunity to weigh in on what measures matter and seem reasonable.  Perhaps it is of some comfort as well to know that, at least early on when these measures are first developed, it seems that they are ‘process measures’ (i.e., are we actually checking appropriate labs, making appropriate referrals, documenting the right words in the chart) rather than the typically more anxiogenic ‘outcomes measures’ (i.e., what those HbA1c values and PHQ9 scores actually are).  Once you get into the territory of actual outcomes measures (honestly, the outcomes that really matter the most), that is when people (understandably) start fretting that it is going to lead to cherry-picking of healthier patients, etc.

For better or worse, this is where the field is moving.  I don’t mean to create anxiety!

Dear Residents,

**This week’s *Fact*:  More about financial incentives for quality**

Last week’s *Fact* involved discussion of performance measures, and I mentioned how such measures are increasingly tied to reimbursement.  That (together with a discussion I had with the PGY3s on Wednesday) had me reflecting more on the issue of financial incentives for quality.  I’m preparing for the next AMA meeting in Atlanta in one week as well, and we are always guaranteed to discuss/debate issues involving incentives for reimbursement at that meeting (one of lots and lots of themes that come up every time we meet).  So, here’s some informal food for thought on several of the major issues surrounding financial incentives for quality that I’ve heard discussed there and other places:

1)  If patients in a clinic or hospital have high quality outcomes, who gets the $$?  Doctor?  Team?  Maybe even some for the patient?

2)  Where is the $$ coming from?  Health care dollars are a zero sum game for the most part.  If there are winners, then there are losers.

3)  Financial incentives could cause conflict in the doctor-patient relationship.  For example, if it is a strong preference of a given patient to not have a given test (e.g., fasting labs because of their atypical antipsychotic prescription), but that patient not having those labs means the doctor gets less money, then there could be some animosity toward the patient, even if we try to fight against it.

4)  Doctors are good at finding possible flaws in data.  Quality outcomes data for their patients are no exception.  These data are often viewed with suspicion.  Part of that has to do with the difficulty adjusting for the baseline severity of different patient populations.

I’m always interested in hearing your thoughts on any of these *Facts*, so don’t hesitate!

Dear Residents,

**This week’s *Fact*:  Root Cause Analyses**

Greetings from the AMA House of Delegates in (neither very sunny nor warm) Atlanta!

We had a fun (okay…maybe not the right word given the nature of material discussed) QI seminar with the PGY3s last week on the topic of Root Cause Analysis.  Many of you have been exposed to this concept at least a little bit, i.e., through some residency exercises we did last year to try to get at the factors contributing to some inefficient processes that were happening in our day-to-day hospital and clinical work.  To remind folks, root cause analysis is a retrospective process for identifying causal factors that underlie events.  It focuses on SYSTEMS, PROCESSES, AND VARIATIONS IN PERFORMANCE.

**How is a root cause analysis done?**

Here is one common approach:  First, assemble your root cause analysis team (controversial if you should involve anyone actually involved in the case—lots of bias and maybe some (understandable) defensiveness!  UWHC now does tend to involve someone actually involved, feeling they have important perspective and insight).  Then, write down a very detailed timeline of events leading up to the undesirable outcome.  Then, use what you leaned from that timeline to map out the possible contributing factors on a cause-effect (i.e., “fishbone”) diagram.  Specifically, brainstorm contributing factors by thinking of all the different problems that might have arisen in any of a number of big categories (e.g., environment, organization, people, tasks, tools & technology, etc).  Finally, determine which are the most likely big contributors, and change the system to fix those things!

**When are these things actually done in real life?**

Well, honestly, at this point, they are most often done when mandated by external entities.  As I’ve alluded to in past *Facts*, health care wasn’t necessarily jumping on the QI/patient safety band wagon all by itself, so we needed help- a big push by regulatory agencies, in particular, the Joint Commission on Accreditation of Healthcare organizations.  They now require that organizations collect data about errors and show they are responding to the error data collected.  In the 1990s, they mandated review of sentinel events (i.e., events which were unexpected and involved death or serious injury or great risk thereof.  This definition includes medication errors, inpatient suicide or sexual assault, child abduction, etc).  JCAHO mandates that the Root cause analysis process is used to analyze these types of events.  And that is indeed what UWHC does.

See [here](http://www.physicianspractice.com/sites/default/files/figures_diagrams/PT%20Wasser%20Fig%202.jpg) (weblink optional reading) for an example of a fish bone diagram from another psychiatric institution, done after an elderly man with bipolar disorder and a complicated medical history was admitted to inpatient psychiatry in the context of an acute manic episode.  He was transferred to medicine for hypotension and rapid a-fib.  After several days on the medical floor, he was transferred back to the inpatient psych unit, only to quickly develop significant hypotension, resulting in a rapid response needing to be called.

Dear Residents,

**This week’s *Fact*:  Alert Fatigue**

A topic that has come up in the PGY3 QI seminars a few times this year has been that of ‘alert fatigue’.  What the heck is alert fatigue, you ask?  It occurs when clinicians become desensitized to safety alerts, and as a result ignore or fail to respond appropriately to such warnings.  This phenomenon occurs because of the sheer number of alerts we receive, and is compounded by the fact that the many alerts generated by electronic medical records (particularly when entering orders) and other health care technologies (e.g., IV pumps, even telemetry) are clinically inconsequential.  Thus, in most cases, clinicians should essentially ignore them, i.e., not change their care based on the warning.  Just yesterday, my EMR warned me that the ibuprofen listed on a patient’s drug list would dangerously interact with the SSRI I was trying to prescribe for her.  Sure, it’s true that a bleeding problem could arise, but in almost all cases, my healthy college kids aren’t going to bleed out from using some ibuprofen with their sertraline.  Does that kind of frequent annoying warning make me pay less attention to more serious warnings I might receive (i.e., a warning about prescribing geodon to someone already on methadone…you can look up why that’s problematic, and why I was happy to receive that alert when prescribing for a patient back in residency)?  Perhaps.  Okay, be honest—yes, yes it does.

So what can be done?  A [2015 Agency for Healthcare Research and Quality (AHRQ) ‘primer’](https://psnet.ahrq.gov/primers/primer/28) (weblink optional reading) on the topic of alert fatigue delineates some suggestions for how these systems can be better designed (not that we’re the ones designing the systems, in most cases…).  An interesting [point](https://psnet.ahrq.gov/resources/resource/23685) (weblink optional reading) made involves the legal consequences of an institution REMOVING alerts from a system once they’ve been added—it would look bad if someone had a negative outcome that seemingly could have been prevented had the alert been retained.

Dear Residents,

**This week’s *Fact*:  apologies and patient safety**

As you know, and as previously discussed in *Facts*, errors happen in health care (duh).  People handle these things differently, and talk to their patients (if at all) about them differently.  Have you heard anything about the fairly recent passage of “physician apology” legislation in Wisconsin?  Basically, prior to passage of this law, if a physician said the words “I’m sorry” to a patient, after a negative outcome happened, that could be used in the court of law as an admission of ‘guilt’ on the part of the physician.  Even if the physician was simply being nice (and human) by saying “I’m sorry x, y, or z happened” and didn’t mean to say they had erroneously caused that thing to happen.  Even if they had no fault in the manner.  Thus, at least in some specialties (I don’t think this is as often discussed in psychiatry as in, say, surgery), physicians were taught not to say those very human words to patients.  The funny (not so funny, actually) thing is that patients are actually LESS likely to sue if doctors say “I’m sorry”, but doctors in Wisconsin (and other states with this kind of legal precedent/law) simply couldn’t risk it by saying those words.  Perverse, isn’t it?

Well, Wisconsin changed its ways with 2014 passage of [“physician apology” legislation](http://www.oncologynurseadvisor.com/web-exclusives/expert-commentary-apology-bill-shields-health-care-providers-apologies-from-malpractice-suits-in-wisconsin/article/346790/) (weblink optional reading).  Health care providers, including doctors, nurses, and others, can now apologize or express condolences without it being used against them in a court of law.  Understandably, [trial attorneys](http://www.nbc15.com/morningshow/headlines/Assembly-to-vote-on-Im-sorry-bill-245955191.html) (weblink optional reading) generally aren’t pleased.

Why mention this now, when it passed in 2014?  For one, I wasn’t writing these *Facts* in 2014 and am now ‘catching up’ on the opportunity to write about these interesting little tidbits!  Secondly, one of the blogs I often read happened to just recently feature a [posting](http://www.mothersinmedicine.com/2015/12/i-screwed-up-and-im-sorry-and-damn-it.html) (weblink optional reading) on the topic of saying ‘I’m sorry’, which reminded me it would be a good patient safety/QI topic to cover in this venue.

Dear Residents,

**This week’s *Fact*:  To Err is Human, 15 Years Later**

Fifteen years ago, the Institute of Medicine released a landmark report (one it’s good to know about!) called “To Err is Human”, which drew big-time attention to problems with patient safety in our country.  It revealed that 44,000-98,000 people die each year in U.S. hospitals due to medical errors (and of course there’s lots more morbidity that results from errors than that number indicates).  Fifteen years out, the National Patient Safety Foundation has released a [report](http://www.npsf.org/?page=freefromharm) (weblink optional reading) providing an update on where things stand since that landmark paper.  Basically, things have improved with the patient safety movement, but not enough.  Many interventions have been effective, but many not so.  The health care system still operates with a low degree of reliability (i.e., no where near the safety levels of the airline industry—everyone loves to compare health care to airline safety!).

The new report makes recommendations in 8 categories to further improve patient safety.  The common theme of these 8 areas?  They are based on the establishment of a total systems approach and a culture of safety (systems and culture….isn’t that ultimately what QI is all about?):

1)  Ensure that leaders establish and sustain a *safety culture* (And by the way….as physicians, you all are by definition leaders, even if you don’t yet consider yourself so.  The important point in this item is that *culture*, defined as the attitudes, beliefs, perceptions, and values that employees share in relation to safety, is key.)

2)  Create centralized and coordinated oversight of patient safety (read:  a national governing body in this area is needed, at least according to this analysis)

3)  Create a common set of safety metrics that reflect meaningful outcomes (so…ONE common set, not a different set for every national organization, state organization, private insurance company, public insurance entity, hospital system, JCAHO, etc….)

4)  Increase funding for research in patient safety and implementation science (Dollars are hard to come by for this line of work!  Not impossible, though, as I know of a single [UWSMPH faculty member](https://www.medicine.wisc.edu/people-search/people/staff/328/Wetterneck_Tosha) (weblink optional reading) who does garner multi-million dollar grants to study this stuff...)

5)  Address safety across the entire care continuum (seems that inpatient settings get the bulk of the patient safety attention currently)

6)  Support the health care workforce (We psychiatrists know this—wellness is necessary to provide safe care.  In a talk I gave to the med students just last week, I shared data showing that lower wellness among med students is shown to contribute to substance abuse, on-the-job cynicism, and decreased compassion….not a recipe for safe patient care.)

7)  Partner with patients and families for the safest care (Patients really are the ultimate stakeholder, eh?  Our Department’s [PFAC](http://www.uwhealth.org/patient-family-advisor/patient-and-family-advisory-councils-frequently-asked-questions/41437) (weblink optional reading) does great stuff in this regard.)

8)  Ensure that technology is safe and optimized to improve patient safety (lots of unintended consequences can occur with ‘new technology’….remember that *Fact* about alarm fatigue?)

Dear Residents,

**This week’s *Fact*:  Barriers to quality**

I recently was looking through the UW med students’ QI materials, and really appreciated how their main QI faculty person presents to them the four big  categories of factors that represent barriers to quality in health care.  So, this week I am sharing with you how these barriers are framed for our med students (and now you know you can shoot the breeze about QI with the students when you’re supervising/teaching/hanging out with them, since they’re now being educated in this stuff too).  Here’s what they are told about the big 4 barriers:

1)  Clinician knowledge, attitudes, and skills:

Stages of acceptance clinicians may experience when shown quality data are:

Stage 1:  “The data are wrong.”

Stage 2:  “The data are right, but it’s not a problem.”

Stage 3:  “The data are right; it is a problem, but it is not my problem.”

Stage 4:  “The data are right; it is a problem; it is my problem and I’m going to do something about it."

Clinicians are also reportedly really good at ‘normalizing deviance’, meaning they become accustomed to problems with quality and safety occurring and not acting because:

-REALLY bad outcomes are few and far between

-It’s just the way it is here

-Change is difficult (all of you who’ve done QI projects appreciate this!!)

-Bad things are likely to happen to really sick people (My honest personal opinion is that there seems to be less ‘acceptance’ of this in the world of psychiatry—the really bad outcomes, no matter how depressed, psychotic, etc our patients are, never really seem to feel okay or expected.)

2)  Patients’ actions and beliefs (i.e., patients do things, and bring variables with them, that we can’t control):

-Patients may or may not follow recommendations (such as taking prescribed meds, following up with us as recommended, or avoiding unhealthy lifestyles or behaviors)

-Patients ask for tests, meds, etc that are not in their best interest

-Probably not all of their problems are remediable with health care resources we currently have available

-How do we measure and account for the above factors??

3)  Organizational culture:

Problematic aspects here especially include:

-the hierarchical nature (doctors above others, resulting in suboptimal communication, lack of teamwork, etc—honestly, the problems in our field here seem less too, or at least different, than in other fields—harken back to your experiences with hierarchies in operating rooms, for example...)

-shame and blame when bad outcomes happen

-perception that high quality 90% of the time is good enough

4)  Complexity of healthcare:

Factors here include:

-disagreement on what is best practice in a given field of medicine (lots of nuance and gray areas)

-always new meds, new technologies, new procedures, and new research findings to assimilate

Dear Residents,

**This week’s *Fact*:  Does peer comparison work?**

It’s generally accepted that physicians are competitive people, for better or worse.  As such, many QI folk believe that giving data to physicians that show where they fall in relation to their peers on any number of QI measures serves as sufficient motivation for behavioral change.  I recall Dr. Diamond sharing with residents his experience on this matter from back when he was working on a state Medicaid initiative on the issue of statewide psychotropic prescribing.  An intervention he undertook was simply sending letters to the Wisconsin psychiatrists guilty of the most occurrences of antipsychotic polypharmacy (defined as 3 or more antipsychotics per patient) and antipsychotic prescriptions to kids <5 years old.  The letters didn’t threaten any action on the part of the state, but simply served as a ‘hey, we noticed this about your prescribing practices; thought you might want to know’ kind of message.  The result was that those prescribers did in fact change their ways, despite there being no teeth behind the ‘FYI’ notices.

Thus, it was with interest that I read a recent article that suggested that this type of intervention doesn’t necessarily change behaviors.  When Medicare sent letters to “overprescribers” of schedule II meds, it didn’t seem to change their ways.  You can read the details [here](http://www.reuters.com/article/us-health-opioid-letters-idUSKCN0W92FD) (weblink optional reading) if interested.  Maybe psychiatrists are more people-pleasing and/or competitive (again, for better or worse) than primary care docs who represented the majority of those receiving letters in this current study?  Maybe it’s something about Medicaid in Ron’s QI project versus Medicare in the current study?  Maybe Wisconsin docs as in Ron’s project are more people-pleasing and/or competitive than docs from other places as in the current study? Maybe there’s something different about controlled substances in the current study compared to antipsychotics in Dr. D’s initiative (e.g., lots more pressure from patients to prescribe Adderall than to prescribe combinations of antipsychotics)?  Maybe Medicare just needs to consult with Dr. Diamond on his approach...

Dear Residents,

**This week’s *Fact*:  Computerized provider order entry and patient safety**

I’ve brought up the issue of computerized provider order entry (CPOE) and patient safety before (alert fatigue for the millionth time, anyone??).  That said, I really liked an [article](https://psnet.ahrq.gov/perspectives/perspective/191) (weblink optional reading) just posted in February on the Agency for Healthcare Research and Quality (AHRQ) website on this topic.  This AHRQ Annual Perspective piece summarizes novel findings and research directions in CPOE over the course of 2015.  I found especially notable their citing of a 2015 FDA (reputable org I think!) white paper on the safety of CPOE systems, with the FDA uncovering significant usability problems with every system they tested.  Such universal problems included:

-poor interoperability among electronic health record systems (imagine the convenience if UWHC/WisPIC Epic and CPRS could connect with each other??)

-lack of efficient two-way communication between physician offices and pharmacies within EHRs (In this day and age we still rely on pharmacies to FAX us refill requests—one step up from carrier pigeons I suppose!  This piece also reminds me of a rather recent bill proposal in Wisconsin that would allow pharmacists to administer injectable meds to patients; while some specialties have expressed concern about this, psychiatrists and primary care docs around here seem to love the concept when it comes to injectable psych meds, since psychiatrists' offices are oft not equipped to administer these meds.  Thus, as it often stands now, psychiatrists send the orders for these injectable meds to pharmacies, patients go and pick them up and may or may not store them as recommended until taking them to their PCPs’ offices for administration, and then the PCP office administers them, hoping they can trust the patient as to what is in the syringe.  I know we generally don’t do it like this within our own WisPIC clinic since we have our wonderful nurses to help with injections, but it happens other places.  Anyways….the one concern psychiatry expressed is that they would want some confirmation from pharmacists as to when they are administering these things to our patients, but this involves extra phone calls and documentation on our parts, when in fact interoperability of EMRs between docs and pharmacists would be a great solution!)

-inconsistent medication naming within and across systems

-vulnerability to wrong-patient errors when multiple records were open (so easy to make this mistake when busy!!)

-a lack of standardized alerts, an abundance of irrelevant alerts (yes, we have established this in prior *Facts*!), and a lack of reasons documented for alert overrides among clinical decision support functions

-medication reconciliation modules that lacked standard terms and did not easily accommodate team-based reconciliation workflows

So, this isn’t to say I don’t appreciate that we have EMRs now—oh yes, I really do.  But, things could surely be better.

It was great seeing you for the Wednesday QI project updates from our PGY3s!  Great job, 3s!  And great feedback on the projects, 1s, 2s, and 4s!

Dear Residents,

**This week’s *Fact*:  Choosing Wisely campaign**

For the 3s and 4s among you, the words “[Choosing Wisely campaign](http://www.choosingwisely.org/)” (weblink optional reading) may sound familiar.  Every year, I ask the PGY3s to try to align their QI projects with already-identified QI initiatives/priorities.  These usually involve QI initiatives that have been identified at the particular sites at which the residents are doing their projects, but I also note that they could involve other entities to which we are held accountable or national initiatives, specifically the “Choosing Wisely” campaign initiatives.

What is Choosing Wisely, you ask?  The goal of the campaign, initially developed by the American Board of Internal Medicine Foundation (the philanthropic arm of the entity that board certifies internal medicine docs), is to reduce waste in the health care system and avoid risks associated with unnecessary treatment. It calls upon leading medical specialty societies and other organizations to identify tests or procedures commonly used in their field whose necessity should be questioned and discussed with patients.  The effort has garnered the participation of over 70 medical specialty societies (including ours—the APA) who have published more than 400 recommendations of overused tests and treatments that clinicians and patients should discuss. The campaign and society recommendations have been included in nearly 300 journal articles and more than 10,000 media articles since the program launched in 2012.  So, we should all know about it, I think.

Here’s what psychiatry has said are [our field’s specific situations to try to avoid](http://www.choosingwisely.org/apa-releases-list-of-common-uses-of-psychiatric-medications-to-question/) (weblink optional reading):

1. Do not prescribe antipsychotic medications to patients for any indication without appropriate initial evaluation and appropriate ongoing monitoring.
2. Do not routinely prescribe two or more antipsychotic medications concurrently.
3. Do not use antipsychotics as the first choice to treat behavioral and psychological symptoms of dementia.
4. Do not routinely prescribe antipsychotic medications as a first-line intervention for insomnia in adults.
5. Do not routinely prescribe antipsychotic medications as a first-line intervention for children and adolescents for any diagnosis other than psychotic disorders.

I was reminded to include this as a topic for a *Fact* when I saw [this recent article](http://journals.lww.com/academicmedicine/Abstract/publishahead/A_Multidisciplinary_Housestaff_Led_Initiative_to.98539.aspx) (weblink optional reading) published in Academic Medicine.  It details Vanderbilt’s “Choosing Wisely Steering Committee” (a committee led by housestaff) and their QI initiative to reduce unnecessary daily BMP and CBC testing on their inpatient gen med and gen surg patients.  

Dear Residents,

**This week’s *Fact*:  5 Whys**

If you attended the Senior Case Conference 2 weeks ago, you heard mention of the "5 Whys" as a specific QI tool that can be used in developing QI projects.  If you haven’t gone through the PGY3 QI curriculum yet, you may be eager for more info on this fun tool!:)  So, here you go...

Specifically, this is a tool useful for uncovering root causes for a given quality or safety problem that you are noticing, and thus helps you to select a way to improve that problem.  And by the way, recall that the November 14th *Fact* addressed fish bone diagrams as another way to glean root causes of problematic happenings in health care (I’m SURE you remember that *Fact*, right?:).  The theory with the tool I’m highlighting today—the 5 Whys-- is that asking the question “Why does that happen?” 5 times will reveal the root causes that contribute to the issue being addressed.

So, for example, a QI project might involve trying to figure out why there is great variability in the degree of urgency of phone calls received by the on-call residents overnight (i.e., why do some patients call in only when a true emergency such as newly dangerous suicidality or psychosis is happening, but others call in for 15 minutes of insomnia?).  You’d run through a series of “Whys” to try to figure out some of the contributing factors that might be fix-able.  Importantly, 5 is just an approximate number of times to ask “why”.  As a general rule, stop asking “why” when you reach the point that the answer to your next “why” is something that cannot be controlled or be acted on within your group (e.g., regulatory requirement, etc).

Incidentally, the conversations that are undertaken with use of this tool might sound very familiar to those of you who’ve been around young children.  For example:

*“Mama, why do I have to eat this broccoli?"*

*“Because it’ll make you grow big and strong.”*

*“Why”*

*“Because it has lots of good vitamins and minerals in it.”*

*“Why?”*

*“Because it’s a bright green vegetable.”*

*“Why?”*

[You pause to do a Google search to find the answer to that one.]  *“Because it contains chlorophyll to help it use sunlight to grow big and strong, just like you’ll grow big and strong by eating it.”*

*“Why?”*

And so it goes.  Such a conversation with a little one, of course, never actually ends with merely 5 Whys and probably actually never gets to a root cause of anything, unlike the surely-very-fruitful 5 Whys exercises that you can undertake as part of QI work.

Dear Residents,

**This week’s *Fact*:  Reconciling patient autonomy and quality improvement**

Two days ago, as part of the PGY3 QI seminar, the third years heard from Dr. Elliot Lee about his infamous [“My Life, My Story”](http://www.med.wisc.edu/news-events/my-life-my-story-serves-veterans-and-their-providers/48791) (weblink optional reading) QI project (see *Fact*from 2 weeks ago).  He noted that at the time he was working on it, the idea of ‘patient-centered care’ was hot in some institutions (i.e., the VA) that were giving out grants for patient-centered initiatives.  Indeed, My Life, My Story seems as patient-centered as they come.

Anyways, Elliot's recounting his project reminded me of an article (written by a med student, no less!) I had recently read in Academic Medicine.  Titled [“Getting (along) with the guidelines: reconciling patient autonomy and quality improvement through shared decision making”](http://journals.lww.com/academicmedicine/Fulltext/2016/07000/Getting__Along__With_the_Guidelines___Reconciling.16.aspx) (weblink optional reading), the author astutely notes that “as health systems answer the growing call for accountability in the form of quality indices, while responding to increased scrutiny on practice variation in the form of pay for performance, a rift is widening between the system and individual patients.”   In other words, while it is efficient and appropriate to make systems chances and not just look for individual bad apple doctors to blame for poor outcomes, it is also important to look at individual docs interacting with individual patients.  How do we continue to assign value to the latter in this era of QI?

The med student-author points out that there are 3 areas inadequately considered by QI-based programs that pay docs largely based on physician adherence to QI guidelines:

1) diversity of patient values and preferences

2)  time and financial burden of therapy in the context of multi morbidity (in other words, worse outcomes and higher costs to provide care, on average, are to be expected with more complicated patients, but not all QI payment formulas take that into consideration)

3) narrow focus on quantitative measures that distract clinicians from providing optimal care

Well-stated, student-doctor-author.  So, I challenge you to consider QI projects that take a systems approach (and thus are likely to be helpful on large scales and are more likely to get buy-in from administrators), but that also are patient-centered and allow for patient autonomy.

Dear Residents,

**This week’s *Fact*:  Lean vs. FOCUS-PDCA**

I’ve been known to point out in QI seminars that I give to residents that it is not as important *WHICH* quality improvement method one chooses, as it is that they choose a method and stick with it.  Those of you who have already completed or started our PGY3 QI curriculum know that the method we use here (and that all of UW Hospital uses) is FOCUS-PDCA.  This involves doing several Plan-Do-Check-Act cycles to find which intervention, or set of interventions, results in the desired improvement to a health care process.

I would be remiss if I didn’t say at least a few words about a ‘competing’ QI approach called Lean. Lean is the drive to devise nimble tasks, processes, and enterprises that maximize value and minimize waste in all its forms.  Most famously, the Toyota Production System has used this approach since the end of World War II, and indeed, Lean has its roots more in industry than health care.  Toyota developed standardized work for each job in the shop, and began to experiment with physical configurations and job pacing that minimized queues and time between operations, signaling systems that allowed upstream operations to respond quickly to downstream conditions, and worker training that emphasized awareness and individual responsibility for quality and problem solving.  The Toyota system has received increasing attention from health care in recent years.  For example, [ThedaCare](https://www.thedacare.org/about-us/ThedaCares-Improvement-Journey.aspx) (weblink optional reading) right here in Wisconsin is widely cited as a health care leader in use of the Toyota-inspired Lean approach.

While most people do feel it important to pick one QI approach and stick with it, we can incorporate elements of Lean into any QI work we do.  I think I use it on a daily basis to try to weed out inefficiencies in my (both professional and personal) life.  For example, if I think about all the steps needed to print out a stimulant script, walk to the printer down the hall to get it, and get it to my waiting patient back in my office, the Lean approach informs how I can improve the process by having me take the patient down the hall with me at the end of the visit, since that hallway is on their way out of the clinic anyways, and that way I get one extra minute of friendly, human time with my patient.  Less efficient and more wasteful would be me walking up and down the hall by myself, doing nothing of use in the process, to get the script.  It may not seem like a big deal, but if you add up, say, an average of 2 stimulant scripts per day x 5 days per week, we could be talking 500 extra minutes of time with patients per year.  I think patients notice these extra minutes.

Do you have journal articles you’ve read in the past that just stick in your mind, for whatever reason?  For me, when it comes to Lean, there is one article that always comes to mind.  It’s an [article](http://www.jgme.org/doi/abs/10.4300/JGME-D-10-00116.1) (weblink optional reading) about optimizing resident rounding on inpatient units.  The authors looked at ways to minimize ‘non value-added time’ during rounds.  The general principle definitely is food for thought, as we are all so busy on every clinical service.  Where can we rid of waste while still providing (or even improving) quality?

Dear Residents,

**This week’s *Fact*:  Measurement-based care**

Measurement-based care (MBC) has become more of a thing in psychiatry.  It is defined as the systematic administration of symptom rating scales and use of the results to drive clinical decision making at the level of the individual patient.  I recall [one of the presenters](http://www.mayoclinic.org/biographies/katzelnick-david-j-m-d/bio-20055365) (weblink optional reading) at the 2009 UW Department of Psychiatry Annual Education Retreat (the theme of which was QI, as a kick-off to our residency program starting a QI curriculum) pointing out the importance of MBC in psychiatry.  He stated “Primary care docs don’t just say “Well, from what you are subjectively telling me, it sounds like your blood pressure is under better control, so I won’t bother measuring it today.”  No.  Rather, they actually measure blood pressure at every visit to get an objective, validated read of it.  He noted that we should do the same in psychiatry, and that the way this is done is via MBC in the form of validated rating scales such as PHQ-9s and GAD-7s.

A new [literature review](http://ps.psychiatryonline.org/doi/abs/10.1176/appi.ps.201500439) (weblink optional reading) has substantiated the use of MBC in psychiatry.  The review noted that measurement-based mental health care significantly improves outcomes, provided that symptom severity data are collected frequently and the results are provided to the clinician shortly before or during the clinical encounter.  These findings were robust and consistent across patient groups, providers, and settings.  According to the study authors, "The time is long overdue for the field of mental health to embrace MBC and live up to the medical testing and treat-to-target principles applied by other medical specialties.  The cost of routinely administering symptom severity scales is minimal, yet the benefits of MBC accrue to all the stakeholders involved, including patients, providers, purchasers, and payers.”

Additionally, MBC is at the heart of the collaborative care model, in which y’all will (or already have) participated at Access Community Health Center and the VA Integrated Care clinic.

Several of our residents’ QI projects over recent years have focused on the use of rating scales, and trying to standardize their implementation in various inpatient and outpatient settings.  This recent study justifies many of our residents having focused their QI efforts on this area.

Dear Residents,

**This week’s *Fact*:  The six aims for improvement, and the one that’s been neglected**

Have you heard of the 2001 Institute of Medicine (IOM) report entitled “[Crossing the Quality Chasm:  A New Health System for the 21st Century](https://www.nationalacademies.org/hmd/~/media/Files/Report%20Files/2001/Crossing-the-Quality-Chasm/Quality%20Chasm%202001%20%20report%20brief.pdf)” (weblink optional reading)?  It is worth mention because of its notoriety within the world of QI and patient safety.  In this report, the IOM described six aims for improvement, specifically calling for health care to be STEEEP:

S=safe

T=timely

E=effective

E=efficient

E=equitable

P=patient-centered

[Folks who do this stuff for a living](http://www.ihi.org/communities/blogs/_layouts/ihi/community/blog/itemview.aspx?List=7d1126ec-8f63-4a3b-9926-c44ea3036813&ID=290&utm_campaign=tw&utm_source=hs_email&utm_medium=email&utm_content=32920825&_hsenc=p2ANqtz-_4x2yvDHJJSWR7NLueE1cf_-enKlCUfxcY_SBOnFLes_1W9MIR5-VrqaohztgT1DF0AmrB_ItmUlrJewTKjnORBM9t4Q&_hsmi=32920016) (weblink optional reading) aptly point out that meaningful progress has been made in all six areas EXCEPT the “equitable” one.  It’s being called “the forgotten aim”.

What can be done?  It might seem an insurmountable challenge to think about developing QI projects that focus on the kinds of upstream determinants of health, institutionalized racism, etc that contribute to inequity in health care.  But that doesn’t mean that awareness of this problem, and attention to any small changes that we can make to try to address it, aren’t worthy of our attention.  As people at the Institute for Healthcare Improvement note:  “…the more we learn about how race, gender, ethnicity, sexual orientation, age, mental health, disability, geographic location, and other factors contribute to health inequities, the more our determination to make a difference grows.  We call on all of you to bring your unique skills, knowledge, passion, and good ideas to those who need them most.”  You can read more, and download a full report on this topic, [here](http://www.ihi.org/resources/Pages/IHIWhitePapers/Achieving-Health-Equity.aspx) (weblink optional reading).

Dear Residents,

**This week’s *Fact*:  Patient shadowing as a patient-centered method for improvement**

I’ve talked about patient-centered care here before.  Today’s *Fact* features this topic again.  Specifically, we’re going to talk here about remembering that patients are stakeholders in most any QI projects, and their input and experiences should thus be taken into consideration.  It seems obvious, but it doesn’t always end up being that way.  We’re often good at including other stakeholders, i.e., clinic managers, nurses, residents, and attendings, who can certainly make or break our projects.  But how do we include the patient perspective?

A [recent article](http://qualitysafety.bmj.com/content/early/2016/05/10/bmjqs-2016-005308.full) (weblink optional reading) had me thinking more about this.  It specifically discusses the use of patient shadowing as a way to get input and perspective from this important group of stakeholders. The article discusses some important and interesting considerations if you are going to undertake shadowing.  For example, should a shadower intervene or offer any sort of medical advice, or are they merely a silent blank slate?  Also, when should a patient be shadowed? Determining the starting and ending points of a patient’s care journey may not be straightforward.  For anyone considering this, I would also recommend checking with our Health Sciences IRB to be sure that IRB approval isn’t needed for this type of activity, as it does directly involve patients, and of course with on-site clinic managers.

The article includes “ten practice principles for shadowing patients”.  I find these practical and useful, so do check them out if you have any inkling of incorporating this sort of QI tool in your current or future QI projects.  Example principles include:

1)  Collect data extensively.  Observe how the patient is treated by care providers, what information he/she receives, what organizational/contextual elements affected the care process, what the patient appreciates, and what he/she perceives as problematic.

2)  Establish criteria for involving patients:  to what extent will they participate in designing and conducting shadowing, analyzing the data, and suggesting improvement opportunities?

3)  Engage clinicians.  Explain the aims of patient shadowing to all the care providers involved in the care pathway, negotiate access with them, and invite them to indicate areas for improvement (i.e., make sure providers aren’t made to feel defensive or like you’re breathing over their shoulder to see what they are doing wrong!).

4)  Patients should be invited to indicate whether the shadowing has captured all the important aspects of their care experience.  If not, the shadowing exercise may be extended/modified.

Maybe it’s just me, but I think it would be really fun and interesting to involve patients in a QI project in this way.  I can think of many of my patients who, I predict, would find it interesting and validating too.

Dear Residents,

**This week’s *Fact*:  Concrete ideas to manage an improvement project**

I like the sound of an article that contains the phrase “concrete ideas”.  It rings of practicality.  So, I was drawn to a recent article on the Institute for Healthcare Improvement (IHI—a big QI site that I follow) website that was titled “15 concrete ideas to manage an improvement project.”  I’ll attach the article here, in case you are interested in reading it in its entirety.  For those of you less inclined to do so (in all your free time….), I’ll summarize a few of my favorites here.  And note that, while these tips would be particularly applicable to current PGY3s doing the QI rotation, or PGY4s doing QI electives, I think there is a kernel of helpfulness in many of these ideas that transcends QI work per se.  Here you go:

1)  Strategy:  Frontload the work.  The beginnings of QI projects are often more work and more challenging because no progress has been made yet.  However, it is critical not to shortcut the planning, including understanding the problem, gathering baseline data, figuring out how you’ll measure results, and organizing the team.

Ideas to try to accomplish this strategy:  Plan time early in a QI project to manage the upfront bolus of work.  For example, hold a 1/2 day process mapping session, or team mini retreat to kick-of the work and organize the project.  At the session, you can figure out who needs to be on your QI team (i.e., who your stakeholders are), finalize your aim statement (i.e., your formal goal for the project), deeply study the steps involved in the process, draft measures, and brainstorm changes to test.

2)  Strategy:  Make it easy.  Since QI work is often added to stakeholders’ regular full-time jobs, successful QI teams leverage existing structures to get work done.

Ideas to try to accomplish this strategy:  Use existing meetings, structures, and one-to-one check-ins to do QI work.  Look for opportunities to swap existing meetings or work for improvement team time and work, or to “kill two birds with one stone”.  An example involves collecting baseline or follow-up data for your QI project.  If your data are to be collected via survey of your fellow residents, then hand out surveys at the beginning of an all-class seminar.  If it’s a survey of attendings, then seek permission to hand out surveys at the beginning of a faculty meeting.

3)  Strategy:  Build a big team.  The most effective QI teams leverage various resources and organize the work to make the most of the human capital at UW.

Ideas to try to accomplish this strategy:  Our QI teams all have assigned QI faculty supervisors, who wonderfully volunteer their time and expertise for this purpose.  In addition to the assigned supervisors, many of our residency teams have found it helpful to identify “consultants” to provide regular guidance to the team.  For example, Dr. David Plante has been a regular consultant on sleep-related QI projects.  Dr. Michael Peterson has been a regular consultant on inpatient-related QI projects when he wasn’t already the assigned supervisor for the team.  Dr. Rick Hafer has been a regular consultant on WisPIC-related QI projects when he wasn’t already the assigned supervisor for the team.

Dear Residents,

**This week’s *Fact*:  Resident attitudes about QI and implications for an effective learning health care system**

In the most recent issue of Academic Medicine (a journal dedicated to medical education at both the med school and residency/fellowship levels, and that spans all specialties), I found an interesting [article](http://journals.lww.com/academicmedicine/Abstract/publishahead/_It_Feels_Like_a_Lot_of_Extra_Work____Resident.98350.aspx) (weblink optional reading) entitled *“It feels like a lot of extra work”:  resident attitudes about quality improvement and implications for an effective learning health care system*.

This article points out that it is all good and well if QI curricula within residency programs are effective in helping their residents learn QI facts and skills.  The bigger goal, though, should be that residents emerge from any QI curricula with a positive attitude toward the practice and an intention to engage in QI activities in the future.  Per the article, “these latter variables are signs of an important culture change—internalization of the principles of QI in terms of both value and practice.  A positive attitude towards QI is important if physicians are going to be leaders in activities to promote a learning health care system throughout their careers.”  Since I want you all to be well-positioned to be health care system leaders, I take the findings of this article seriously!  It is my responsibility to fashion your QI curriculum with these tenets in mind.

So what did the article report?  The research involved focus groups with residents from multiple specialties doing QI projects at the University of Utah (hello Katie Steingraeber—maybe she was a part of it).  What emerged from those focus groups were facilitative factors for culture change within residents taking QI curricula and barriers for the same.  The facilitative factors, i.e., the aspects of their curricula that imparted residents with a positive sentiment toward QI, were:

-QI projects that could double as academic research projects (I bet this is especially true in programs with both research requirements and QI requirements)

-immediate results from their QI interventions

-opportunities to direct what is going to happen to health care in the future

-having an obvious, easily achievable goal that felt useful and supported by all (can I have this characteristic everywhere in my life please?)

-basic literature search, or other aspects of the QI project, that felt truly educational

-groups being built around shared interests related to the QI project (always feels good to build community, I think!)

The barriers, i.e., the things that left residents feeling not so great about QI, were categorized into four themes as follows:

Theme 1:  Competing goals make understanding the vision, purpose, and philosophy of QI challenging (representative resident quote:  “I feel like patient ratings are really…the institution is making a push for patient satisfaction, which is important, but I think there have been studies that show that changing your actions to please patients’ expectations  may not be the best thing especially when it comes to pain and when it comes to things that are safety issues for patients as well, that maybe the physician is better qualified to do and that’s just something in general.”)

Theme 2:  Confusion (representative resident quote:  “I don’t even know what process to go about working on or implementing or who to talk to to begin a quality improvement initiative.”)

Theme 3:  We are not a valued/valuable part of the process (representative resident quote:  “When we’re asked to implement an intervention and study the results and we’re not allowed to [choose the] intervention.  That is frustrating.”)

Theme 4:  Prioritizing our QI work is difficult given other responsibilities (representative resident quote:  “Everybody is strapped for time as is…we don’t have any kind of set-aside time when we’re supposed to.”)

So, there you go.  I aspire to inculcate our QI curriculum with things that leave you feeling interested and inspired, but not overburdened or confused.  Up to date on regulatory requirements and prepared for the health care world of tomorrow, but not cynical.  Please do let me know if you have any thoughts on this balance within our own QI curriculum!  I value your input always.

Dear Residents,

**This week’s *Fact*:  Failure Modes and Effects Analysis (FMEA)**

At our PGY3 QI seminar two weeks ago, Dr. Peterson teamed with Jan Haedt (risk management expert at UWHC) to walk the PGY3s through a root cause analysis of an actual inpatient psychiatric case.  Every year I learn new things by sitting in on this experience, and one thing I learned this year was the term “failure modes and effects analysis” (FMEA).  Have you ever heard of this?  I love what it represents—a model of *proactive* intervention in the world of patient safety.  Sometimes quality improvement and patient safety can seem *reactive*.  That is, a bad outcome happens, so then you do a root cause analysis, M&M conference, QI project to fix the systemic flaw, etc.  There’s nothing wrong with the latter approach, but it’s nice to have a preventative approach to patient safety as well.

Anyways, so what it is is this:  a systematic, proactive method for evaluating a process to identify where and how it might fail and to assess the relative impact of different fails, in order to identify the parts of the process that are most in need of change.  This includes review of the following:

1)  steps in the process

2)  failure modes (what could go wrong?)

3)  failure causes (why would the failure happen?)

4)  failure effects (what would be the consequences of each failure?)

So, FMEA is particularly useful in evaluating a new process prior to implementation and in assessing the impact of a proposed change to an existing process.  A random example might involve development of a new protocol to try to prevent medication errors  on an inpatient unit.  The different ‘failure modes’ (i.e., ways that med errors could occur—step #2 above) might include:

1) wrong dose prescribed

2)  med prescribed for wrong patient

3)  order unsuccessfully sent to/processed by pharmacy

4)  missed drug-drug interaction

5)  missed patient allergy

6)  patient not appropriately identified prior to dispensing the med to him/her

Conveniently, you can find an FMEA tool online for your use [here](http://www.ihi.org/resources/pages/tools/failuremodesandeffectsanalysistool.aspx) (weblink optional reading).

Dear Residents,

**This week’s *Fact*:  The problem with “work arounds"**

Last week, I wrote about the concept of “failure modes and effects analysis”, a topic inspired by Dr. Peterson’s recent QI seminar with the PGY3s.  Well, his seminar was ripe with inspiration for other topics for *Facts* as well, as something he mentioned in that seminar also inspired today’s topic:  the problem with “work arounds”.

Work arounds are seemingly small work process problems in which people in a health care setting consciously or subconsciously decide to adapt their routines to accommodate or avoid a problematic issue with work flow.  Since I’ve become more QI-minded, I try diligently to avoid work arounds.  If I find myself doing one, I ask myself how I can try to help fix the underlying problem so that the process becomes more efficient, without a need for a work around.  That said, I’m still guilty of these things!  For example, there is some problem in my EMR in which patients directly referred to me (as opposed to going through the usual triage intake process in my clinic) have something wrong with the way in which their home address is entered in the EMR.  This prevents me from being able to e-prescribe for them.  I should fix the system flaw, and instead, thus far, I have just been doing the work around of calling their scripts in via phone.  I’m embarrassed to say that.  It seems that in any spare moment in between patients or meetings, I don’t have the 5 minutes it would take to send an email to get to the root of the problem.  So, instead, I spend 1 minute calling in a script for each of these patients.  I cringe at the amount of time I would have saved overall if I’d have just fixed the problem when it first occurred.  Remind me to fix it when I’m done writing this email!

Another issue that can keep  people from directly addressing work arounds is that they don’t want to be the “squeaky wheel” that complains about things.  Well, I recently read an [article](http://www.quickbase.com/blog/steve-spear-on-process-improvement-avoid-zombie-like-thinking) (weblink optional reading) that referred to organizations full of people who don’t want to be squeaky wheels as “zombie organizations”.  In these organizations, information goes missing, instructions aren’t clear, resources aren’t allocated efficiently, and communication is poor, but leaders and teams just keep going—like zombies.  Now don’t get me wrong—UW and our Department definitely are far from zombie organizations!  That said, I think we all can easily slip into that zombie mindset, and I challenge you to observe when that happens and take action to try to get to the root of any problems in work flow that you see happening around you!

Dear Residents,

**This week’s *Fact*:  4 strategies for sustaining improvement in health care**

During the early days of our PGY3 curriculum (2009), we had some great QI projects developed by residents, but not all of them were 100% sustained after the residents completed their QI rotation.  I recall Dean Krahn, MD, MS (esteemed prior QI project supervisor at the VA) saying to me (very kindly and supportively) at that point “Claudia, maybe the next step for your curriculum is teaching the residents to make their QI projects sustainable, so the efforts don’t just die when the residents move on.”  Wise words for sure.  So, since then, we specifically ask that our PGY3 QI projects have some element(s) to help ensure sustainability.  It was with interest, then, that I just read an [article](https://hbr.org/2016/11/4-steps-to-sustaining-improvement-in-health-care) (weblink optional reading) in the Harvard Business Review with 4 strategies for how to sustain QI efforts in healthcare.  They note the interesting finding that efforts to sustain improvement often have one thing in common:  they engage and standardize the work of frontline managers.  Here’s a synopsis of their strategies:

1)  Choose a pilot unit within the organization.  A pilot unit should have stability (low staff turnover), alignment around goals (managers should understand why standardizing work is important to sustaining quality), management “hygiene” (stable staff schedule, not a sense of chaos in the environment), and engagement (the unit should have a respected local champion who can build excitement for change and encourage participation).

2)  Start with the immediate supervisor at the point of care.  For example, this might be the manager of the front desk office staff at WisPIC, or the charge nurse on an inpatient unit.

3)  Use early wins to build momentum.  Thus, especially in the early stages, be cautious about asking staff to achieve complex, hard-to-achieve goals that require significant interdepartmental teamwork.  Doing so may demoralize the team and set them up for failure if things don’t work right off the bat.

4)  Motivate frontline clinical managers by tackling what irks them.  This is probably self explanatory, but it basically ensures buy-in.  These types of problems have been called “rocks in your shoes”—the daily irritants that frontline managers want eliminated.

There you go.  As the article states, "No one wants to toil away at a health care improvement effort only to see that progress disappear as systems and processes revert to the old way of doing things…  Improving care isn’t enough; having a systematic approach to sustaining improvement is equally important."

Dear Residents,

**This week’s *Fact*:  6 ways to tailor your QI work to reduce disparities**

I recently ran across an [article](http://www.ihi.org/communities/blogs/_layouts/ihi/community/blog/itemview.aspx?List=7d1126ec-8f63-4a3b-9926-c44ea3036813&ID=330) (weblink optional reading) on the topic of QI projects that address health care disparities, and it made some important points.  It notes that "If health care hopes to achieve equitable care for all patients, providers have to understand that quality improvement can actually *worsen* health disparities.  When quality improves for one racial group (often the group that’s already doing better) at a faster rate than for others (often the groups already doing worse), quality for the whole population improves, but the gap between subpopulations widens. Focusing on the whole population rate obscures the fact that the disparities for some subpopulations are actually worse than before.”

Well, that’s definitely a compelling point!  The article goes on to describe 6 key activities that folks working on QI projects can undertake in order to reduce, rather than exacerbate, disparities:

1)  Collect and analyze data by patient race, ethnicity, and language

2)  Center at the margins: begin improvement work by considering the needs of the disadvantaged

3)  Test change ideas that address patients’ social conditions and cultural influences

4)  Establish trust between providers and patients (e.g., systems can do this by conducting focus groups with patients to understand their preferences and attitudes about care, and designing comfortable, safe experiences for them, where providers take pains to treat them with dignity and respect)

5)  Provide accessible primary care tailored to the needs of the marginalized people (this one probably not as relevant to our sphere of work)

6)  Use the required Community Health Needs Assessment as an opportunity (under the Affordable Care Act, at least as of now, the IRS requires not-for-profit hospitals to conduct a Community Health Needs Assessment at least once every three years; hospitals then are to develop and execute an implementation strategy along with a set of performance metrics to meet the needs identified in the Assessment)

Dear Residents,

**This week’s *Fact*:  Resident workflow and psychiatric ER consultation**

I’ve said it time and time again:  residents are well-positioned to do QI projects, because they are so often at the front lines of clinical work and thus very acquainted with any inefficiencies and “rocks in the shoes” of clinical practice.  Because residents do psychiatric ER consults around the clock, 364 days per year, it is perhaps no surprise that this general area has been the topic of many QI projects within our residency.  And our program is not alone.  *Academic Psychiatry* just published an article from the UCLA psychiatry residency program entitled “Resident workflow and psychiatric emergency consultation: identifying factors for quality improvement in a training environment.”  I’ve attached the article for your perusal, if you are so inclined.  Here are the highlights of the article:

1)  The residents conducting the study used (Survey Monkey) survey methodology not unlike what many of our resident QI projects use.  Their survey included elements hypothesized to enhance or impede workflow, and measures pertaining to self-rated efficiency and stress, when it comes to doing ER consults.  They also conducted a focus group with residents.

2)  The study identified several factors subjectively associated with enhanced workflow when doing psychiatric ER consultations.  Interestingly, one of the approaches that they reported that some residents used and found helpful was giving capable patients self-reported history forms to complete while they were waiting for a consultation; that precisely was [the QI project](https://www.ncbi.nlm.nih.gov/pubmed/24883148) (weblink optional reading) of our esteemed QI faculty supervisor, Dr. Fred Langheim, not so many years ago.  Other approaches that some residents found helpful for optimizing efficiency included using written templates to guide interviews, dictating notes immediately after interviews, planning carefully to walk between work locations (e.g., the patient’s bedside and the nursing station) as little as possible, and “not feeling compelled to document more exhaustively than is needed for patient care".

3)  The study identified several factors subjectively associated with impeded workflow when doing psychiatric ER consultations, especially difficulty with documentation (completing the consult note was identified as the #1 impediment to efficiency identified), struggles to communicate with patients’ families, and a small number of “repeat offender” (their words) faculty members who didn’t respond promptly to pages when on call.

4)  Some of the things the program is working on as a result of the findings of this report include developing a brief curriculum for communicating efficiently and effectively with families of patients in the ER, and promoting techniques such as use of templates early in training so they become habit.

5)  As the authors point out, workflow optimization (and much of QI really) is inherently local, and so we cannot necessarily draw specific interventions from this study.  However, the general approach to a QI study such as this, and the general topic areas identified, may be of interest for folks considering future QI projects.

Bottom line is that much of this study addressed the basic tension between efficiency and being comprehensive.  I face the pain of that basic tension every day at work, and heck, in every aspect of life really.  I know you do too.  QI is probably a tool that can help us with finding a reasonable balance between those things.

Dear Residents,

**This week’s *Fact*:  An expert’s take on root cause analysis**

Last Friday, I had the pleasure of participating in the GME office-sponsored PGY1 Patient Safety Orientation.  I specifically worked with a group of most of our PGY1 psychiatry residents, together with some residents from other specialties, in working through a root cause analysis (RCA).  It was great fun, and our PGY1s were awesome and made me proud.  It was particularly timely, then, that as I was going through some QI-related articles right after that activity that I had saved up to read whenever the opportunity arose, I came across an [article](https://psnet.ahrq.gov/perspectives/perspective/211) (weblink optional reading) on RCAs.  Specifically, the article involved an interview with the founding director of the VA National Center for Patient Safety.  He was actually a former astronaut (impressive) and wrote a big white paper on how RCAs could be improved.  Here are some highlights of what he had to say about RCAs:

1)  He hates the term “root cause analysis”.  I’ve heard Dr. Peterson mention this as well:  RCA is a misnomer, because rarely is there just a SINGLE root cause that contributed to an adverse event.  Moreover, the process is not JUST the ANALYSIS of the event.  Rather, it also includes implementing systems changes to keep it from happening again.

2)  Few institutions look at “near misses” (close calls that do not result in actual patient harm, but that easily could have) when doing RCAs.  The article points out that they should, as it’s a missed opportunity to prevent bad outcomes before they happen.  (CR note:  UW Health does sometimes look at near misses when doing RCAs.  In a nutshell, RCAs at UWHC come about when a hospital committee reviews all of the Patient Safety Net (PSN) reports that they receive, as well as all the situations that mandate an RCA based on JCAHO and other requirements.  If a PSN report is assigned a “harm score” of 6 or greater, or if a near miss was associated with a particularly terrible possible outcome, it gets an RCA).

3)  Most cases examined via RCA are not blame-worthy, i.e., they aren’t the result of a criminal act, a caregiver under the influence of alcohol or illicit drugs, or intentionally unsafe practice.  Thus, there should be no punitive outcome from them.  Folks asked to participate in an RCA should be informed of this.  Blame-worthy acts are investigated via a different mechanism.

4)  You cannot just cherry-pick the contributing factor that happens to appeal to you or the one that’s easy.  If you don’t address all of the contributing factors, the chance of having *sustainable* good outcomes is minimal.  As the astronaut points out, “This is real work.  You have to allow time to do it.  And you cannot do it *sort of*like this."

Dear Residents,

**This week’s *Fact*:  Psychiatry M&Ms**

Many of you probably know that Dr. Garlow has recently taken over from Dr. Moore as faculty lead on our M&M conferences.  Just as Dr. Moore was, Dr. Garlow is eager and excited to involve the psychiatry residents in the M&M conferences.  M&Ms will continue to be a part of the PGY3 QI rotation, in which each PGY3 presents the patient case at 1 M&M over the course of their PGY3 year (this is not a case in which they were personally involved).  Along these lines, I came across an article (attached) in the most recent *Academic Psychiatry* issue on the topic of M&Ms as part of psychiatry residency programs.  The article, authored by the Yale psychiatry residency program, noted that the goals of their psychiatry M&Ms are that participants will have an increased:

1)  comfort discussing their own errors as well as communicating with colleagues without fear of blame or disrespect

2)  recognition of systemic influences on adverse outcomes

3)  awareness of the needs, strengths, and vulnerabilities of other disciplines/specialties, and

4)  sense of agency in improving systemic coordination of care

While our own M&Ms haven’t had an explicit set of goals per se, those sound spot on to me.  I hope our M&Ms capture these themes.  Psychiatry M&M is a rare thing among psychiatry departments/residencies/clinics/institutions.  Wherever you end up practicing, you could certainly take this model of a quality improvement and patient safety initiative with you.  You would have a lot to offer, I think.

Dear Residents,

**This week’s *Fact*:  6 patient safety “resolutions” for 2017**

I get much of my information on the latest and greatest in the world of QI from the Institute for Healthcare Improvement.  Their President and CEO, Derek Feeley, just published his proposed [six patient safety “resolutions” for 2017](http://www.ihi.org/communities/blogs/_layouts/15/ihi/community/blog/itemview.aspx?List=7d1126ec-8f63-4a3b-9926-c44ea3036813&ID=365&utm_campaign=tw&utm_source=hs_email&utm_medium=email&utm_content=43069180&_hsenc=p2ANqtz-_189fsuO8JPSuNGHgNE65Dz53Ukbo0nn_xSCf1VEMNoddDJ6P-4cNiYlcP0v3sMR2FXYmLmhcW3HqI-KyPitMHg_PeTQ&_hsmi=43069180) (weblink optional reading).  That is, these are the 6 things he feels that today’s health care context requires when it comes to QI and patient safety initiatives.  Here they are:

1)  *Focus on what goes right as well as learning from what goes wrong*

2)  *Move to greater proactivity* (this focus on the positive is a theme already here, eh?)

3)  *Create systems for learning from learning* (this means that we need a comprehensive, systems-focused view of safety in order to sustain and further the improvements made through *individual*QI projects….so true, but what a daunting task!)

4)  *Be humble—build trust and transparency* (that is, we can’t improve safety if we can’t be completely open and transparent with ourselves, our colleagues, and our patients about safety issues)

5)  *Co-produce safety with patients and families* (well, this has surely been a theme of some of our weekly *Facts*, yes?)

6)  *Recognize that safety is more than the absence of physical harm; it is also the pursuit of dignity and equity*(yes yes yes…the kind of care we would want for our own family members, and for every single member of our communities)

Dear Residents,

**This week’s *Fact*:  How research and QI can work together**

Many people ask how QI is different from research.  There are important differences, but also many similarities.  I cover some of this in the PGY3 QI seminar series.  To highlight some factors:

1)  **Both generate new knowledge, but in different ways.**  Research helps us understand what works, often by comparing an intervention with a control.  QI helps us test interventions (sometimes already evidence-based per existing research) in our own *real-world* settings, making adaptations  to those interventions if necessary.

2)  **Both methods are grounded in the basic scientific method**.  That is, both involve starting with a hypothesis, designing an experiment to test it, and collecting data to determine results.  In QI, those steps usually occur in the form of multiple small tests of change called PDSA cycles. In research, there is usually one big test.  Research controls for confounding factors so we can be more definitive about cause and effect linkages.  QI is “applied science” with biases and confounds often abundant.  In QI, we modify our hypothesis as we gain more data/complete more PDSA cycles.  In research, that would be a no-no.

3)  **Both rely on data to guide practice**.  Research helps us demonstrate which interventions have evidence to justify attempting their use in a general type of setting.  QI helps us demonstrate which interventions are going to work in *our* particular setting.

How, then, can research and QI work together?

1)  A QI project should bring together knowledge from within a site’s own team (based on your own experience working within that particular setting), from outside the team (e.g., from other clinics in the community that have tackled the same problem), and from the research base (where interventions have proven effective via a pure science approach).

2)  Research findings can help us find opportunities for QI work.

3)  The sequential testing of ideas through QI at a given site may ultimately build an increasing degree of belief that a change can lead to improvement in multiple settings and contexts.  That belief may lead to your publishing or presenting your QI project, or designing a formal research study.

Dear Residents,

**This week’s *Fact*:  “What matters to you?"**

In 2012, a NEJM [article](http://www.nejm.org/doi/full/10.1056/NEJMp1109283) (weblink optional reading) introduced the concept of asking “What matters to you?” in addition to asking patients “What’s the matter?”  From what I’ve read, this concept has gained unexpected traction around the globe as something of a quality initiative.  The [Institute for Healthcare Improvement](http://www.ihi.org/communities/blogs/_layouts/15/ihi/community/blog/itemview.aspx?List=7d1126ec-8f63-4a3b-9926-c44ea3036813&ID=368&utm_campaign=tw&utm_source=hs_email&utm_medium=email&utm_content=43452535&_hsenc=p2ANqtz-_SvQltv4UGH38KRoK72OVAvkEbftsB86W-GhOws2geVXsg7hrMxn0D92cPO7Lnj_RWevnFAaERn8i2QoZfUSIgd77L4w&_hsmi=43452535) (weblink optional reading) recently delineated reasons why they feel this idea has translated so well into different cultures, languages, and care settings:

1)  When you ask someone what matters to them, you are compelled to see them as a whole person, and not a disease (e.g., schizophrenia) or an organ or a lab result.  You appreciate them as a unique individual who deserves respect.  (Dr. Diamond often reminds us of this important concept, I think).

2)  Asking this question can restore joy and meaning to the lives of health care providers.  When you pause and have this real conversation, it can remind you of why you wanted a career in health care in the first place.  When we are so busy, stressed, tired, etc, it can be hard to remember such things.

3)  This question started as a way to enhance the care experience for patients, but turns out it can also address the cost reduction and population health aspects of the [Triple Aim](http://www.ihi.org/resources/Pages/Publications/TripleAimCareHealthandCost.aspx) (weblink optional reading) of health care. For example, you may be assuming a patient wants some particular treatment (e.g., expensive new brand-name medication, a controlled substance, etc) or seemingly unnecessary diagnostic test, but turns out they just wanted to be heard and understand, or something else less controversial or resource-intensive than you imagined what they wanted would be.

It seems this “What matters to you?” thing would be a concept particularly amenable to use in psychiatry.  In fact, in some ways it reminds me of the [Cultural Formulation Interview](http://praxis.psychiatry.wisc.edu/index.php?id=976) (weblink optional reading) out of the DSM-5.

Dear Residents,

**This week’s *Fact*:  The Eight Wastes**

I’ve mentioned here before my love of the “Lean” mindset and approach when it comes to quality improvement.  This is an approach to QI projects that looks to minimize wasted time/effort/resources, and thus results in more time being available for things that really matter (i.e., quality face-to-face time with patients).  I was thus drawn to a talk on this topic from Mass General that I recently encountered.  Specifically, I wanted to draw your attention to their apt description of the “eight wastes” of healthcare, i.e., wastes that can and should be minimized when you take a Lean approach to improving a system:

1)  Defects (not meeting specified requirements or not correcting defects):  Examples include med errors, wrong patient, missing or incomplete information, blood redraws, and misdirected results.

2)  Overproduction and production of unwanted products (ties up more resources than necessary):  Examples include extra lab tests (maybe labs were already drawn at a patient’s outpatient clinic before inpatient admission and needn’t be redrawn), unnecessary imaging, and unnecessary antibiotics or other meds.  The premise behind this category of waste reminds me of the [Choosing Wisely campaign](http://www.choosingwisely.org/societies/american-psychiatric-association/) (weblink optional reading).

3)  Waiting (things that increase wait time or delay response time to the patient):  Examples include waiting for test results, records, information, transport, patients, staff, or discharge.

4)  Not utilizing employees:  This includes not considering for implementation any ideas for improvement as seen through the eyes of any members of the health care team.

5)  Transport (movement of materials or people):  Examples include unnecessary movement of patients/meds/specimens/samples/equipment, including transport between different clinics or hospitals.

6)  Inventory (ties up capital and invites risk of obsolescence and damage):  Examples include any drugs, supplies, equipment, phone or in basket messages, or junk mail just sitting around not being used or addressed (it all takes up physical and/or mental space in our lives!).

7)  Motion (movement by workers):  This includes time wasted searching for patients, meds, charts, supplies, or paperwork or navigating long clinic halls (i.e., is your patients’ waiting room the one closest to your clinic office?).

8)  Extra processing (creates delays without adding any benefit and invites more defects in the process):  Examples include unnecessary bed moves, retesting of lab specimens, repeat paperwork, repeat registration, and readmission.

May you go forth and develop eyes for waste, so that you can eliminate it.  And too go forth and enjoy this last day of not being [required to check the PDMP](http://host.madison.com/wsj/news/local/health-med-fit/wisconsin-doctors-must-check-patient-history-before-prescribing-opioids-other/article_44e23856-2fc1-5bf2-ba97-47b8b6fc7b4e.html) (weblink optional reading) before most prescriptions of controlled substances.

Dear Residents,

**This week’s *Fact*:  Engaging learners in health system quality improvement efforts**

It was lovely seeing most of you on Wednesday as we heard updates from the PGY3 QI teams on their projects to date.  As you heard, they have been doing outstanding work on important issues impacting quality of care in our own health systems.  We can look forward to hearing their final QI results during June Grand Rounds!  Now, on to the issue at hand….

I hope the title of this week’s *Fact*didn’t make your eyes glaze over. I know it sounds trite.  But bear with me, as I welcome your feedback on this issue.  I came across a recent [article](http://journals.lww.com/academicmedicine/Abstract/publishahead/Engaging_Learners_in_Health_System_Quality.98288.aspx) (weblink optional reading) in *Academic Medicine* by the same title.  It caught my eye because hey, you are my learners!  And I’m trying to engage you in QI efforts!

So what about this article?  The authors provide their perspective on how good of a job med schools and residencies are doing at meaningfully engaging their learners in QI work that results in sustainably improved care.  Their conclusion?  Med schools and residencies are not doing a good job.  The authors suggest that what is needed to improve the situation is ***bidirectional alignment*** (okay…now I know your eyes are glazing over….) within the organizational culture.  This means that an organization’s QI activities should originate from the vantage point of learners *as well as* from leaders.  This approach is advantageous because:

1)  learners bring a fresh perspective to the workplace that differs from other members of the workforce

2)  seasoned veterans bring their experience

3)  learners have few preconceived notions about the health care milieu, making them open to innovative approaches

4)  learners are already in a learning mind-set and can share a learning approach with the experienced frontline staff

You are obviously engaged in QI efforts as part of our curriculum that predominantly occurs in the PGY3 year—so whether you are engaged or not is not an issue.  I try to strike a balance between having y’all choose a topic of great interest to you for your projects, while also prepping you for the post-residency world in which any QI efforts you undertake are going to be vastly more likely to get institutional support if they align with institutional QI priorities.  So, that balance at this point means that I ask you to align your projects with some existing QI priority (e.g., a priority at WisPIC, the VA, UWHC, APA’s Choosing Wisely campaign, etc).  As long as there is some tangential bearing on one of those priorities, you can run with your project however you, your partner, and your supervisor see fit.  Does that constrain residents too much?  Or does it err on the side of too much freedom (i.e., not enough prep for post-residency QI work)?  I’d love to hear your thoughts on this aspect of ***bidirectional alignment***.

Dear Residents,

**This week’s *Fact*:  Starting with the end in sight**

For those of you involved in the Clinical Educator Track, you know that it’s recommended that, when developing a new med ed curriculum, one of the first things you do is write learning objectives.  You do this BEFORE developing the actual curriculum, as it frames what should go into the curriculum.  It also tells your stakeholders (learners in this case) what you expect them to have accomplished by the end of the experience.

An analogous situation applies to QI projects.  You FIRST develop an aim statement for the QI project, and then you develop the project that will hopefully achieve the aim.  Thus, the aim guides the project.  Those who have done the PGY3 QI curriculum have heard me drill the meaning of an aim statement several times, but for the 1s and 2s among you, an aim statement is a goal statement for a QI project that answers the following questions:

*How good?* (What do you expect to happen?  Specify your goal and quantity that would suggest satisfactory QI project outcomes.)

*By when?*  (What’s your time frame?  The time frame should create some urgency, and also prevent the work from becoming a never-ending improvement project.)

*For whom?* (Indicate the population you plan to serve.)

So, for example, drawing from one of the current QI projects:  “By June 2017 (by when?), 80% (how good) of outpatient psychiatry residents and WisPIC front desk staff (for whom?) will be satisfied with communication between these two groups”.

Finally, it’s important that an aim inspires new thinking and learning, and not fear and demoralization.  An aim statement should not passive-aggressively imply that one group is doing a terrible job at something (that may be true, but be congenial—while also honest--about wording).  If you are setting ambitious numerical goals for your improvement efforts, you must ensure that the necessary resources are available to allow for a fundamental change.  I’ve often said to residents to err on the side of lower numerical goals, as systems are hard to change.  In the first year or two or three of our residency’s QI projects, everyone wrote aim statements that aimed for 100% improvement in everything (by the end of the academic year, 100% of providers will be happy, 100% of patients will have perfect care, etc).  It doesn’t necessarily feel good to write an aim statement that aims for less than 100% (doctors want perfection), but the reality that perfection is challenging to achieve, to say the least, is important to recognize.

May you all go forth and achieve your aims.

Dear Residents,

**This week’s *Fact*:  5 essential steps of shared decision making**

It’s definitely a buzz phrase in health care these days:  “shared decision making”.  It refers to docs not being paternalistic in saying ‘Here’s your one option—this is what we are going to do.”  Instead, the treatment approach is one of joining with the patient and helping her/him to understand the options and make a reasoned decision  Some of us may lament the focus on this type of approach, as it could seemingly take longer to come to a decision about what to do for a given patient.  Some may feel it doesn’t provide enough guidance for the patient.  On the other hand, if it allows a patient to leave our offices fully wedded to the treatment plan on which we decided, that could save some headaches (i.e., “non-compliance”) later.  AHRQ posits that there is a 5-step process for shared decision making that  goes like this (using the handy acronym “SHARE”):

**S**:  Seek your patient’s participation (some patients truly do just want you to tell them what to do)

**H**:  Help your patient explore and compare treatment options (you are the one with the medical training, after all)

**A**:  Assess your patient’s values and preferences (e.g., side effects, co-pays, etc that matter to one patient may be inconsequential to another)

**R**:  Reach a decision with your patient (and sometimes the patient’s decision may be to delegate the decision to someone else—realize that too can be an informed decision on the part of the patient)

**E**:  Evaluate your patient’s decision (this would occur in follow-up sessions, in which you monitor the extent to which the treatment decision was implemented, assist your patient with managing barriers to implementation of the plan, and revisit the decision in the first place)

See attached for more details and tips regarding this approach.

Dear Residents,

**This week’s *Fact*:  7 spreadly sins of QI projects**

In the world of QI at large, improvement teams often take some missteps when it comes to successfully sharing and spreading their QI projects within their organizations.  The [Institute for Healthcare Improvement](http://www.ihi.org/resources/Pages/Tools/IHISevenSpreadlySins.aspx) (weblink optional reading) (IHI—a go-to resource for me) developed a list of the “Seven Spreadly Sins” (cute, eh?) to give QI teams practical tips for overcoming challenges to successfully sharing/spreading their QI projects.  Here they are (and see attached for a nifty illustration of these 7 points—feel free to print out a nice color version for your own offices:)):

1)

**Sin**:  Expect huge improvements quickly, and then start spreading right away.

**Do this instead**:  Create a reliable process *before* you start to spread.

2)

**Sin**:  Don’t bother testing—just do a large pilot.

**Do this instead**:  Start with small, local tests and *several* PDSA cycles.

3)

**Sin**:  Give one person the responsibility to do it all.  Depend on “local heroes.”

**Do this instead**:  Make spread a team effort.

4)

**Sin**:  Rely solely on vigilance and hard work.

**Do this instead**:  Sustain gains with an infrastructure to support them.

5)

**Sin**:  Spread the success unchanged.  Don’t waste time “adapting” because, after all, it worked so well the first time.

**Do this instead**:  Allow some customization, as long as it is controlled and elements that are core to the improvements are clear.

6)

**Sin**:  Require the person and team who drove the initial improvements to be responsible for spread throughout a hospital or facility.

**Do this instead**:  Choose a spread team strategically and include the scope of the spread as part of your decision.

7)

**Sin**:  Check huge amounts of data infrequently.

**Do this instead**:  Check small samples frequently so you can decide how to adapt spread practices.

Dear Residents,

**This week’s *Fact*:  QI analogy from the PGY1s**

I have a few favorite QI-related quotes that I find very compelling.  Here is one of them:

“A bad system will beat a good person every time.”  (W. Edwards Deming)

I was reminded of this quote when I was giving an introductory QI seminar to our PGY1s two days ago.  One of the PGY1s astutely noted that the systems approach and philosophy in QI reminds him of sports teams.  That is, it doesn’t matter how talented individual athletes on a team are; if there is no team cohesiveness, or no systems in place to optimize team work or team-wide functioning, then the team will fail (fellow PGY1 Vuong Vu chimed in that this was a problem with the [2012 L.A. Lakers](http://www.silverscreenandroll.com/2013/5/9/4313942/what-went-wrong-with-the-2012-2013-los-angeles-lakers-coaches)) (weblink optional reading).  Likewise, QI is not about blaming individual docs/nurses/pharmacists/social workers/etc for not trying hard enough or being talented enough.  It is about optimizing the functioning of the whole health care team and system.  It doesn’t matter how smart your individual health care providers are; they must function well as a system.

May Deming's quote, and the inspiration given to us by our PGY1 colleagues, set the tone for a QI-positive Friday.

Dear Residents,

**This week’s *Fact*:  New dementia quality measures**

For those of you who receive daily APA Psych News alerts in your inboxes, you may have already seen this recently.  Updated [quality measures](http://ajp.psychiatryonline.org/doi/full/10.1176/appi.ajp.2017.17401) (weblink optional reading) for the care of patients with dementia were just published on May 1 by a joint work group of the APA and the American Academy of Neurology.  It covers 9 areas of assessment, diagnosis, and treatment of dementia.  Coverage of the release of this document highlights the important and potentially controversial addition to the quality measures that looks at the percentage of patients who are informed that they have been diagnosed with dementia.

The Co-Chair of the work group noted that “Because we do not currently have treatments that can definitively alter the course of dementia, clinicians are sometimes apprehensive about revealing a diagnosis of dementia because of the concern that patients will be very demoralized.  In fact, the data show that people want to know the diagnosis and want to be able to make plans.”  That makes sense to me.  I would have some worry in these circumstances for sure about demoralizing patients, but at the same time, I personally would want to know, if I were the patient.  So, this is another example of a QI initiative that hopefully aims to increase the degree to which we are treating patients as we would ourselves or our own closest loved ones.  Here’s what our own geriatric psychiatrist Dr. Walaszek had to say about these new measures, when I asked him about them:  "These quality measures highlight that, though we do not have treatments that significantly affect the course of the illness, there are a number of ways in which we can improve the lives of people with dementia, and their families and caregivers."

Dear Residents,

**This week’s *Fact*:  The weekend effect**

Have you heard of the “weekend effect”?  This is the idea that patients have worse outcomes if they are admitted to hospitals over the weekend as opposed to during the regular work week.  You can imagine all sorts of possible explanations for this, but one that has received much attention is the idea that hospitals are staffed differently (i.e., with fewer staff in general, fewer attendings, etc.) on weekends.  Indeed, the hospital can feel like a different place on weekends, so that sort of hypothesis might make intuitive sense.  An oft-cited NEJM [paper](http://www.nejm.org/doi/full/10.1056/NEJMsa003376) (weblink optional reading) reported that patients have a 30% higher mortality rate if admitted on weekends.  But that says nothing about reasons for the worse outcomes.  So, what does the research show about reasons?  If QI initiatives were/are to be undertaken to address this problem, root causes must be understood.  Well, it has finally been studied!  [Findings](https://psnet.ahrq.gov/resources/resource/30974) (weblink optional reading) show that a big part of the explanation for higher weekend admission mortality is that patients who are admitted on weekends are sicker to start with.  Of course—that makes sense.  Weekday admissions are more likely to be “planned”, whereas weekend admissions may be more likely to be unplanned emergencies that cannot wait for the patient to see their regular outpatient doc on Monday.

While the above studies weren’t on psychiatric patients per se, it is possible that similar patterns would be found in the ranks of our patients.  Planned psych admissions as coordinated by our patients' outpatient psychiatrists (i.e., during regular weekday hours) are likely to be less emergent than are unplanned ER visits by patients on the weekend.  Maybe that reflects what you’ve observed?

Dear Residents,

**This week’s *Fact*:  Patient safety and patient engagement**

“Patient safety” may be defined as that subset of quality improvement designed to prevent medical errors that harm a patient or near misses that could have harmed a patient.  I’ve intermittently  authored *Facts* about the topic of patient safety, often regarding how patients themselves have contributed to patient safety initiatives.  I just read a variation on that theme.  It’s an IHI article entitled “[Patient Engagement and Patient Safety:  One and the Same](http://www.ihi.org/communities/blogs/_layouts/15/ihi/community/blog/itemview.aspx?List=7d1126ec-8f63-4a3b-9926-c44ea3036813&ID=408) (weblink optional reading).”  Essentially it says that all the effort involved in making care safer and more reliable shoed be in the service of engaging patients and families. The authors note that in exceptionally safe and reliable organizations, patients and families are as much members of the care team as are clinicians and other health care staff. Their list of ways in which patients and families can be engaged in their care toward the end of optimizing patient safety is as follows:

1)  **Leadership and accountability**:  For each care episode (e.g., an outpatient visit, inpatient stay), patients and the rest of the health care team need to agree on a set of goals and clearly define roles and accountability for what it takes to achieve these goals.  Clinical team members advice on the clinical components, and patients give their perspectives until there is agreement on what constitutes a reasonable goal.  When people are in accord and feel accountable, there is a higher likelihood of success.

2)  **Psychological safety**:  Patients should feel psychologically safe to share their concerns with the clinical team.  We are psychiatrists so yes, we understand this need.

3)  **Negotiation**:  I’ve shared this point with you before….to help the care team determine if the patient has the will to make changes we are suggesting, this involves a shift from asking “What is the matter with you?” to asking “What matters to you?”.  From there, treatment goals can be negotiated based on what is salient to all parties.

4)  **Transparency**:  Transparency with patients and families is important because it removes the fallacy of clinical team infallibility.  So, for example, if a provider fails to appropriately inform a patient about a lab result, the authors attest that responding to the event in a timely and effective manner promotes trust, healing, and learning.  As patients thus understand that the health care organization is trying to improve processes to enhance safety and reliability, patients will recognize the need for their engagement in the system.  For example, they might feel more activated to take responsibility for directly obtaining their test results and to otherwise close gaps if they occur.

5)  **Improvement and measurement**:  Patients are valuable assets when it comes to improvement because they bring their unique perspectives, particularly about how they experience care delivered by an organization.  One way to involve patients and families in improving care is to ask for input on their experiences and ideas, and share data with them about ongoing improvement efforts.  Our PGY3 QI project teams have the open invitation to discuss their project ideas with our Department’s [PFAC](http://www.uwhealth.org/patient-family-advisor/patient-and-family-advisory-councils-frequently-asked-questions/41437) (weblink optional reading) (Patient and Family Advisory Council) in order to get this valuable patient perspective.

There you go.  In sum, without engaging patients and families in our QI work, we could fall short of our goals to improve our systems to provide the safest, most reliable of care.

Dear Residents,

**This week’s *Fact*:  The Triple Aim for Health Care**

I have a few different overarching goals in authoring these QI Facts of the Week:

1)  Infuse a humanistic perspective on QI and patient safety, to counter some/much of the information on these topics that you might encounter that might make it seem that QI is all about checking boxes and satisfying payers (that said, I know that some of the *Facts* do touch on the reality of the need to check boxes at times)

2)  Provide a regular reminder about the importance of and role of QI/patient safety throughout the course of your residency, where possible tying it in to current events and other residency curricula.  In this way, I hope to keep your QI experience from feeling like a single bolus of education in your PGY3 year that doesn’t at all relate to the rest of your life/education/profession.

3)  Make sure you are familiar with all the relevant terms and concepts when it comes to QI, so that you are well-poised to contribute to QI initiatives in your future practices.  When someone touches on QI in your future job interviews, I want you, our residents, to be uniquely positioned to delve into a meaningful conversation on the topic.  And more importantly, when you then start those new jobs, I want you to be well-positioned to meaningfully improve health care in the systems in which you are working, since you will be hitting the ground running with a strong base of knowledge on the topic already in place.

That said, it’s high time for me to introduce (or remind some of you of) the phrase “[triple aim for health care](http://www.ihi.org/resources/Pages/Publications/PrimerDefiningTripleAim.aspx)” (weblink optional reading).  This helps fulfill goal #3 above, i.e., hopefully helps you to be familiar with the more prominent concepts out there when it comes to QI.

So what is the Triple Aim?  This is a framework developed by the Institute for Healthcare Improvement (big QI enterprise that I often reference) that focuses on simultaneous pursuit of the following 3 things by health care organizations for the populations they serve:

1)  improvement of the patient experience of care (this means care should be safe, effective, patient-centered, timely, efficient, and equitable)

2)  improvement of the health of populations (because improving *health* is a challenge that requires the engagement of partners across the community to address broader determinants of health; we are short-sighted if we think health is mostly determined by care that goes on in our offices…)

3)  reduction in the per capita cost of health care (see #2 above…spending lots of $$$ on clinical care per se is far from the whole answer to making people healthy…we need money available for use in other ways that promise to improve health)

Note that the Triple Aim is really a SINGLE aim, with 3 dimensions.  If you google Triple Aim, you’ll get millions of results, so you could read about this forever if you wanted.  In sum, though, it’s a framework developed over years, with hundreds of organizations and lots of coalitions and countries.  It is a lofty but noble goal for our organizations and communities.  As we do work in any one of those three dimensions, we should consider how the other dimensions are simultaneously going to be affected.

Dear Residents,

**This week’s *Fact*:  More on communication and patient safety**

Last week I spoke with the PGY3s for a couple of hours on the topic of patient safety.  So, in the spirit of patient safety, I bring to you a related item today.  I had attended an [educational session](https://wire.ama-assn.org/life-career/better-communication-patients-linked-less-burnout) (weblink optional reading) at my most recent AMA meeting earlier this summer on the topic of the relationship between better communication with patients, improved patient outcomes, and less burnout.  I’ve been meaning to highlight that session here since then, so here goes.  Here are some facts and takeaways from that talk:

1)  Fewer than 50% of hospitalized patients are able to identify their diagnosis or the names of their medications at discharge (how good are we at explicitly discussing actual diagnoses with patients?)

2)  1/3 of adults with chronic illness underuse their prescriptions due to cost (do we explicitly ask about this?)

3)  Patients and physicians disagree on the main problem 50% of the time

4)  Ineffective team communication has been demonstrated to be the root cause for 2/3 (!) of all medical errors

5)  As patients perceive physicians and staff as more caring, actual health outcomes improve

6)  When physicians improve communication with their patients, they (the physicians) experience less burnout

So, in sum, better patient-physician communication improves care, decreases risk of medical errors, and increases physicians’ joy at work.

Dear Residents,

**This week’s *Fact*:  Case study in how QI can save physicians lots of time**

Have I mentioned how much I appreciate the LEAN approach to improving the efficiency of any aspect of systems of care?  Yes, yes I believe I have.  I love it because it’s a way to eliminate non-value added time in your life/day.  We only have 24 hours in a day, eh?  One can certainly take this mindset to an extreme, but I for one could use 30 hours in a day, and thus, anything that optimizes efficiency is a win (assuming quality isn’t compromised).  So, I was drawn to a recent headline in the WMJ (formerly the “Wisconsin Medical Journal” but now a regional journal, such that WMJ no longer is actually an acronym) that reads “How physicians can save 56 hours per year”.  It’s a letter to the editor from a doc at Dean Clinic in town.  You can read the full thing [here](https://www.wisconsinmedicalsociety.org/_WMS/publications/wmj/pdf/116/2/52.pdf) (weblink optional reading).  The upshot of the article:  Docs (not necessarily just psychiatrists—in this case, primary care docs) may spend up to 75% of their time related to a patient visit doing non-face-to-face tasks.  Dean wanted to take a LEAN approach to minimize any unnecessary non-face-to-face time included in that 75%.  They discovered that, on average, their PCPs were spending LOTS of time logging into the EMR:

-81 log-ins per day

-7 to 12 seconds per log-in

-“misfires” (typing the log-in password information incorrectly) 24 times per day

Their LEAN approach involved changing to a system of using ID badges on readers next to computers in their department in order to log-in.  Providers now log in just 2 full times per day, as opposed to 125 per day as before.  This has resulted in a time savings for the average clinician of 17 minutes per day, which is 76.5 minutes per week, which is 56 hours per year (hence the title of the WMJ letter) PER PHYSICIAN (multiple that by the total number of physicians in the organization and you get a really, really big number).

So, don’t underestimate the time savings that can be had from LEAN approaches to improve seemingly minor inefficiencies in your day.

Dear Residents,

**This week’s *Fact*:  11 tenets of a safety culture**

In one of the recent UW Health Physician emails that I received, there was an article on Patient Safety Culture.  Naturally, I read it.  From it, I learned that UW Health just adopted the recently released “Joint Commission 11 Tenets of a Safety Culture”.  In sum, these tenets are recommended for organizations in order to develop a transparent, non-punitive culture of reporting and learning from adverse events, near misses, and unsafe conditions.  You can find the 11 tenets [here](https://www.jointcommission.org/assets/1/6/SEA_57_infographic_11_tenets_safety_culture.pdf) (weblink optional reading).  And by the way, isn’t it interesting that there are 11?  I can just imagine the meetings that were had, trying to pare it down to 10—that nice, round number—to no avail.  Sometimes you just have to give in and accept that a list of 11 items is needed, and apparently that was the case here.  To highlight 2 of the 11 that I particularly like:

1)  Use clear, just, and transparent risk-based processes for recognizing and distinguishing human errors and system errors from unsafe, blameworthy actions.  (CR:  Even with all our QI-based discussion of systems issues impacting care, of course unfortunately there still are blameworthy causes of poor care too, and we must distinguish these two.  For example, if someone tries to render patient care while drunk, that is a blameworthy situation.)

2)  Recognize care team members who report adverse events and close calls, who identify unsafe conditions, or who have good suggestions for safety improvements.  Share these “free lessons” with all team members (i.e., feedback loop).

Dear Residents,

**This week’s *Fact*:  Patient safety culture and physician wellness culture**

In past *Facts*, I’ve reflected on issues related to a culture of patient safety, i.e., a health care environment in which people feel comfortable drawing attention to medical errors and near misses, without fear of punishment, toward the end of improving health care systems.  There is an increased emphasis on this type of culture everywhere you look.  In very recent years, there is simultaneously an emphasis on a culture of physician wellness, i.e., a health care environment that promotes sustainability of physical practice and that fights against issues that contribute to burnout.  The last time I sent a message about patient safety culture, Art wrote back to me with a reflection on physician wellness culture, and if/how/when these two types of cultures might intersect with each other.  Do they have any relationship to each other?  Does an emphasis on one such culture make things more or less amenable to the other type of culture?  For example, if we put systems in place to make it more comfortable for people to discuss near misses, does that lead physicians to feel less stressed/burnt out because they feel they can safely discuss these things?  Or does it contribute to an overall sense that ‘Your practice is being monitored.  You better not make a near miss, because it’ll be reported by everyone around you the second it happens, and then you could be in big trouble.’  Great questions, Art!  I surely don’t have the answers.  That said, it is a point worthy of reflection.  Ideally, I would think, we’d have both a patient safety culture and a physician wellness culture in perfectly harmonious coexistence.  We’d want them to complement each other.  There can be no patient safety if all physicians were burning out, eh?  Along these lines, I just read [this article](https://wire.ama-assn.org/life-career/vivek-murthy-md-turns-focus-physician-well-being?utm_source=BulletinHealthCare&utm_medium=email&utm_term=093017&utm_content=physicians&utm_campaign=article_alert-morning_rounds_weekend) (weblink optional reading) featuring US Surgeon General Vivek Murthy, MD, and his reflections on physician wellness.  He notes the importance of, among other things, the following components to physician well-being:

1.  sleep

2.  physical activity

3.  social connection

4.  meditation

5.  gratitude

I know….in all your free time, right?  But still worth noting.  Dr. Murthy points out that if you are dealing with chronic stress, you can’t do your job well, safely, or sustainably.

Dear Residents,

**This Week’s Fact:  Patient safety discussions**

A recent [article](https://www.ncbi.nlm.nih.gov/pubmed/?myncbishare=uwisclib&otool=uwisclib&cmd=Search&term=%09Improving+Patient+Safety+Communication+in+Residency+Programs+by+Incorporating+Patient+Safety+Discussions+Into+Rounds&submit2=Go) (weblink optional reading) I just read describes an innovative way of increasing the extent to which patient safety discussions happen during the course of a residency program.  The study involved residents from 5 different specialty programs (including psychiatry) at a single institution.  Residents who were on inpatient rotations during a 2-month period participated in *patient safety discussions* during rounds.  Residents who were not on inpatient services served as controls.  Inpatient faculty attendings received weekly text reminders to ask 3 questions designed to engage residents in patient safety discussions during rounds:

1)  Did anything happen today that resulted in harm or could have resulted in harm to your patient?

2)  Was this a system error, a process error, or a human error?

3)  Is this a solvable challenge, and if so, what are the appropriate reporting mechanisms?

The results demonstrated that residents who participated in patient safety discussions during rounds were more likely than the control residents to:

1)  report patient safety events

2)  describe improved communication

So, for all of you on inpatient services, and really for all of us anywhere (inpatient and outpatient alike), consider thinking about these questions as you are rounding on your patients or seeing them in whatever setting you are seeing them.  Maybe such considerations could enhance the culture of patient safety.

Dear Residents,

**This Week’s Fact:  Americans’ experiences with medical errors and views on patient safety**

As you might have noticed, I heavily utilize the [Institute for Healthcare Improvement (IHI)](http://www.ihi.org/Pages/default.aspx) (weblink optional reading) and the [Agency for Healthcare Research and Quality (AHRQ)](https://www.ahrq.gov/) (weblink optional reading) to stay up-to-date on QI/patient safety related issues.  The most recent interesting [article](https://psnet.ahrq.gov/resources/resource/31487) (weblink optional reading) I read, commissioned by IHI and featured on the AHRQ website, was titled “Americans’ experiences with medical errors and views on patient safety.”  It presented the results of an update to a similar 1997 survey, and in doing so shared the results of input from 2536 American patients.  Here are some highlights:

1)  20% of patients reported personally experiencing a medical ERROR, most often in the outpatient setting.

2)  10% said they experienced HARM when receiving medical care (thus, the difference between the stat in item #1 and the one in item #2 here highlights the difference between error (which could include near misses in this survey) and actual harm).

3)  The most common type of error reported was a missed or delayed diagnosis.

4)  The second most common type of error reported was communication error.

5)  Patients with higher socioeconomic status and higher health literacy are more likely to say they have experienced a medical error.

6)  Those who reported experiencing medical errors identified an average of 7 factors that contributed to the error (i.e., this underscores the idea that many/most errors are not the result of single medical mistakes by single providers, but rather multiple SYSTEMIC problems).

7)  Most who experienced an error were not informed of the error (i.e., they figured it out on their own).

8)  In 1/3 of cases, errors went unreported by the patient or by someone acting on the patient’s behalf.

9)  When cases of medical errors are either reported by the patient or relayed to the patient, health care providers and facilities accept responsibility about half the time (note this is not a commentary on my part as to if this is good or bad—these reported errors are per patient perception, and we don’t know if in fact they actually all were errors per se).

10)  Most who reported the error did so in an effort to prevent it from happening to someone else (I note this is a definite theme in what I have read on this topic.  A take-home for me personally is that when I make medical errors, what patients really want to hear from me/us that we are taking steps to fix the system so it doesn’t happen again).

11)  And to end on a positive note:  Most Americans say patient safety has stayed the same or gotten better over the past five years, and few think they will experience a medical error when receiving care.

Dear Residents,

This Week’s Fact:  Presenteeism

What’s presenteeism?  It means coming to work even when you are significantly ill, and it has consequences for patient safety.  I bet we’ve all done it.  I’ve surely done it, and I’m embarrassed to think back on a couple of occasions particularly as a med student when I came to work on M3 rotations despite being quite ill.  At the time, I was thinking that that is what a dedicated, hardworking med student/physician would always do—suck it up for the good of the team.  But oh my gosh…was that really for the good of the team and patients?  To bring all my germs with me?  Especially since having my little ones, I think about how, if my own child were in the hospital, I would be quite angry if an obviously sick med student/physician came in the room to touch my kiddo or provide care in any way.  I would now be THE mother described as follows in [this article](https://psnet.ahrq.gov/perspectives/perspective/234) (weblink optional reading) I just read on presenteeism:

*"I remember very distinctly a mother, she was one of those mothers who might by some people be labeled as tough because she was demanding and protected her child. If a health care worker went into that child's room and had any kind of symptoms, she would kick them out. I don't blame her. She was absolutely right."*

The article goes on to describe the patient safety concerns that exist with coming to work when ill, including infectious risks and non-infectious risks (e.g., more likely to make errors of all kinds).  The interviewer in the article points out that:

*"Some who read or listen to this will say this is more evidence that medicine is going to hell in a hand basket. In the old days, doctors had this intense commitment to their patients and would slog through the snow to get in to do the work they needed to do and work when they're tired, work when they're sick. Now we're creating this shiftwork mentality. These readers may say "Sure, this is all fine if you're going to work in a box store, but medicine is different fundamentally.””*

The interviewee’s astute response is:

*"This may be one of the areas where this transition to shift work may actually improve performance. Those doctors who slogged through the snow and so forth were extraordinarily dedicated, and most often there was nobody else who could do it. So they felt "Well, even if I'm not at my best I'm better than nothing," which is often the alternative. In modern American health care, except maybe under extraordinary circumstances (very small towns, rural medicine), that should no longer be the alternative. Physicians today, young physicians, care just as much about their patients as any other generation did. People who say that are asking the wrong question. The right question is: given what we have and who we are, what is the very best we can do for our patients? Working when you're sick is absolutely not the* *very best that we can do for our patients, period."*

Dear Residents,

**This Week’s Fact:  VIP Syndrome**

Have y’all heard of VIP Syndrome?  This refers to the adverse influence that famous or wealthy patients can have on the medical judgment of otherwise excellent clinicians.  What does this have to do with QI/patient safety?  Well, it’s a topic that came up during a recent QI seminar, as we were discussing some of the factors that can lead to patient safety events.  Say, for example, you are treating another physician (say, another resident physician from a different specialty) in your WisPIC office.  How might VIP syndrome manifest?  There are countless examples, but here are some that come to mind immediately:

1)  The resident-patient has a very busy schedule, which you can certainly appreciate.  To make it easier on them, you offer to see them at 4:30 pm during clinic wrap up meeting, or at 5 pm “as long as they get there to check in before the front doors are locked”.

2)  The resident-patient has an eating disorder.  For the typical eating disorder patient, you’d order labs including electrolytes.  You’re “pretty sure” this patient’s labs will be normal, since, let’s face it, 90% of the time that is the case.  And you don’t want to inconvenience the busy resident.  So, you don’t order labs.

3)  The resident-patient presents with the CC of “I am pretty sure I have ADHD.”  You are pretty sure they do too (despite the obvious academic accomplishment of having graduated med school with apparently untreated ADHD).  They prefer that you don’t talk to their parents, because it’s an embarrassing issue to them, and they don’t want to bother their parents.  They admit to having smoked weed before starting residency (they are now a PGY1), but they attest to not having done so since then.  You start concerta at the first visit.

So, VIP syndrome can result in us treating VIP patients differently than we treat our other patients.  In this case, different often doesn’t mean better, even though it might feel like we are doing the patient a favor.  One of the principles of QI is looking at where there are variations in the care that we provide patients.  Variation can be a marker of a problem, and that certainly can be the case when it comes to VIP Syndrome.

Dear Residents,

**This Week’s Fact:  More on patient satisfaction**

Long-term readers of these *Facts* may recall my prior musings on the topic of patient satisfaction (as one purported measure of quality) and its relationship to all sorts of things, including whether more satisfied patients actually have better health outcomes (in many circumstances, [probably not](https://www.theatlantic.com/health/archive/2015/04/the-problem-with-satisfied-patients/390684/) (weblink optional reading), and in fact, in inpatient medical units, more satisfied patents may be more likely to die), and how patient satisfaction relates to whether or not we give them controlled substances if they ask for them.  A couple of years back, I referenced this by linking to Michael Miller, MD’s (he’s an addiction psychiatrist and one of our volunteer clinical faculty) *JAMA* [feature](https://jamanetwork.com/journals/jama/article-abstract/1148339) (weblink optional reading) on the topic, in which noted significant concerns with use of patient satisfaction as a measure of quality of care.

Well, along these lines, you might have noticed a [study](https://jamanetwork.com/journals/jamainternalmedicine/article-abstract/2664068) (weblink optional reading) published earlier this week in *JAMA Internal Medicine*that found that denial of several types of patient requests is associated with lower patient satisfaction ratings of the physician.  Specifically, denials of requests for the following were associated with significantly worse patient satisfaction:

1) referrals

2)  pain meds

3)  other new meds

4) laboratory tests

In contrast, denials of requests for antibiotics and imaging studies were not associated with worse patient satisfaction.  The authors note that this is not surprising, since doctors have increasingly been trained how to counsel patients about the dangers of unnecessary antibiotic use and unnecessary radiation in the form of imaging studies.  However, we’ve had less training in how to decline patient requests for those other categories.

I really appreciate the sentiment expressed from one of the authors, which goes beyond simply bemoaning these findings and whining that our health care system is going to hell in a handbasket, and rather, looks at something we can do DESPITE the momentum that patient satisfaction measures have:

“In an era of satisfaction score-driven compensation of clinicians, the findings suggest the need to explore the utility of training clinicians to better handle patient requests, potentially optimizing the patient experience while also enhancing clinician career satisfaction.”

The educator in me really appreciates that, as it means we can TEACH and LEARN how to deal with these requests.  I feel my own discussions with UW student-patients (in my clinic at University Health Services) who request stimulants, and for whom I feel they would be an inappropriate choice, have been an evolving work in progress.  I’m sure there would be a way to teach doctors how to do this more effectively and efficiently.  I wonder if this should be a part of our QI curriculum?  If anyone is looking for a Clinician Educator Track project, I’d love to talk with you about this topic (pending Art’s, Stu’s, and Jen's blessing, as they are more aware of how it would fit in with our entire residency curriculum, of course).

In summary, then, I bring you a gem of a quote from the Atlantic article to which I linked above:  “Patients can be highly satisfied and dead an hour later."

Dear Residents,

**This Week’s Fact:  QI projects to improve screening for metabolic side effects of antipsychotic medications**

Within our own UW Psychiatry residency program, the most common locus of resident-chosen QI projects has been the consult service.  I suspect this is because there are so many SYSTEMS issues that arise when you are dealing not only with your own specialty, but also with dozens of other specialties, floors, attendings, residents, and other professionals from other services, etc.  It’s interesting to compare that to the world of QI in psychiatry at large.  My read of the literature in this regard is that the most common focus of QI projects, among all psychiatry QI projects that people CHOOSE TO PUBLISH, is screening for metabolic side effects of antipsychotic medications.  It’s a neat and tidy metric in our specialty, I suppose.  The [article](https://www.ncbi.nlm.nih.gov/pubmed/?myncbishare=uwisclib&otool=uwisclib&cmd=Search&term=%09A+Randomized+Controlled+Trial+of+a+Patient-Centered+Approach+to+Improve+Screening+for+the+Metabolic+Side+Effects+of+Antipsychotic+Medications&submit2=Go) (weblink optional reading) I just saw published in the *Community Mental Health Journal* (the journal of the American Association of Community Psychiatrists) is no exception.  Here’s the low-down:

1)  Study setting/population/type:  RCT that tested whether a computerized, patient-centered intervention that educated Veterans with serious mental illness about metabolic side effects of antipsychotics and encouraged them to advocate for receipt of metabolic monitoring would increase rates of monitoring

2)  Results:  no difference between intervention group and comparison group, both of which had relatively high rates of metabolic monitoring.  And many individuals in both groups had persistently abnormal metabolic parameter values despite high rates of monitoring, contact with medical providers, and receipt of cardiometabolic medications.

3)  Reflections:  This isn’t necessarily the main point of the paper, but what I reflect on is that the world of QI in psychiatry often remains “stuck” at the level of “ process measures”.  In this case, the process measure is ‘did we check fasting lipids and glucose as per recommended frequencies in patients on antipsychotics’.  We don’t always/often get to the ultimately more meaningful measures that look at if patient outcomes actually improve.  In this case, did their cholesterol and blood sugars end up better controlled?  And even more meaningful:  did fewer patients die from metabolic sequelae?

Dear Residents,

**This Week’s Fact:  E-prescribing’s potential to reduce outpatient psychiatric med errors**

Dear Residents,

E-prescribing is held up as a large scale QI/patient safety initiative that has the potential to reduce medication errors across specialties.  Hence, e-prescribing is among the “meaningful use criteria” established by Medicare in order to receive full reimbursement for services provided to Medicare patients.  It does seem logical that replacement of paper-based (how is your handwriting these days?) and phone-based prescription systems with electronic ones would seem safer.  We are evidence-based folks (recent [news](http://thehill.com/news-by-subject/healthcare/365204-trump-admin-bans-cdc-from-using-evidence-based-and-science-based) (weblink optional reading) about the CDC notwithstanding), though, so what do actual data show about this?  Conveniently, a [study](https://www.ncbi.nlm.nih.gov/pubmed/?myncbishare=uwisclib&otool=uwisclib&cmd=Search&term=realizing+e-prescribing%27s+potential+to+reduce+outpatient+psychiatric+medication+errors&submit2=Go) (weblink optional reading) was just published looking at this very issue within our own specialty (the first such study to look at medication prescribing errors within the world of OUTPATIENT psychiatry, as opposed to those that have already been done in the inpatient psych world).

Alas, med prescribing errors still happen in the outpatient world, even with the advent of e-prescribing.  Some reasons probably include:

1)  growing complexity of med regimens

2)  alert fatigue from an abundance of on-screen automated alerts

3)  reduced opportunities to interact with our pharmacy colleagues (unlike in the inpatient setting, where that dialogue is still commonplace)

The just-published manuscript suggests steps for outpatient psychiatrists/systems to take in order to continue to reduce medication prescription errors, including the following:

1)  accelerate implementation of e-prescribing in outpatient psychiatric settings (from where we sit, it might seem like everyone uses e-prescribing.  Oh not true in the world of psychiatry!  Ours is a specialty that still has a large % of private practitioners, and private practitioners often don’t have the resources to implement these kinds of things.  While Wisconsin’s health care landscape is one of large health care systems and affiliated clinics versus private practices, that is not the case many places.)

2)  address design flaws in e-prescribing systems (e.g., possible solutions include use of clear graphical user interfaces, integrating required drop-down menus, autocompleting a med’s administered amount, and appropriate balancing of automated alerts with alert fatigue).  In sum, a thorough human factors engineering approach (see the most recent *Fact*!) is needed.

3)  improve interoperability among proprietary e-prescribing systems (the holy grail!)

4)  improve interoperability of e-prescribing systems and retail pharmacy electronic systems (a secondary component of the holy grail!; at a very minimum, the opportunity to e-discontinue meds at a patient’s pharmacy would be helpful, but this has proven hard for anyone to figure out!)

5)  advocacy and education:  of all specialties, across the nation and world, psychiatry is often the last to benefit from system improvements.  Advocacy is needed to ensure our specialty and our patients aren’t left behind if/as the above initiatives are implemented.  One could engage in a whole separate debate about some of these issues (e.g., would some of our patients argue against full interoperability, since they might feel information in our notes is more stigmatized than that in other specialties’ notes?  Rebecca Radue is a resident among us who hears first-hand the debate on both sides of some of these issues, through her simultaneous roles on the Wisconsin Medical Society Council on Legislation and the NAMI Wisconsin leadership team—ask her about it sometime!)

Dear Residents,

**This Week’s Fact:  EMR alerts to reduce opioid and benzo coprescribing**

Last Friday, I had the good fortune of leading a small group of PGY1s to run through a simulated root cause analysis (RCA) as part of the UWHC GME-sponsored Patient Safety Orientation.  My small group was almost entirely our own PGY1 psychiatry residents, with a handful of interns from other specialties mixed in.  Anyways, the patient situation for which we did an RCA involved an older patient who was admitted to the hospital with pneumonia and ended up falling and breaking her hip while hospitalized (note this was not an actual UW case and isn’t protected information).  As we talked through the RCA and potential contributing factors, one area on which we focused was the fact that she was coprescribed an opioid and a “sleeping medication” (which was implied to be a benzodiazepine), and it was while those were both in her system that she became confused and fell.

The final part of any RCA activity is developing some action steps to try to remedy some of the presumed contributing factors to the adverse outcome (or near miss).  Our group discussed a number of possible interventions for the polypharmacy-of-sedating-meds issue.  One such intervention discussed was some sort of EMR alert when a benzo and opioid are coprescribed.  Our astute PGY1s (who made me very proud of their QI prowess in this context) pointed out that alert fatigue could be a concern with such an intervention.  That is, we are all used to so many alarms and beeps and alerts as we go through our EMR inputs that we can end up disregarding those that really matter.

It was with great interest, then, that precisely 40 hour after discussing this very issue at the Patient Safety Orientation, I came across the first and only [article](https://www.ncbi.nlm.nih.gov/pubmed/?myncbishare=uwisclib&otool=uwisclib&cmd=Search&term=Electronic+Medical+Record+Alert+Associated+With+Reduced+Opioid+and+Benzodiazepine+Coprescribing+in+High-risk+Veteran+Patients&submit2=Go) (weblink optional reading) on the topic of EMR alerts to attempt to reduce coprescription of benzos and opioids.  It was just published a couple of weeks ago.  Admittedly, it has to do with the outpatient world, and it involves the VA setting, which has some EMR advantages that the rest of the EMR world doesn’t have.  That said, I found it instructive.  It described a QI project that evaluated the effectiveness of an EMR medication alert that was issued when a benzo and opioid were coprescribed.  The relevant nuance here is that the alert was only issued when the coprescription occurred with Veterans with known high-risk conditions (substance use, sleep apnea, suicide risk, >age 65).

The results demonstrated that coprescriptions of the two categories of meds significantly decreased at the intervention site, compared to a control site.  The authors speculated that their nuanced, targeted use of the alert only with high-risk populations helped minimize the alert fatigue factor.  Thus, it was hoped that those physicians receiving the alert had less of a ’this doesn’t apply to me’ automatic reaction to such alerts.

Dear Residents,

**This Week’s Fact:  The SQUIRE Guidelines for publishing QI projects**

Dear Residents,

Have you heard of the SQUIRE Guidelines for publishing QI projects?  SQUIRE stands for “Standards for Quality Improvement Reporting Excellence”.  These are guidelines that were first published in 2008 to provide guidance to authors (and reviewers) of QI projects, with the aim of published QI studies being as clear, precise, and complete as possible.  The SQUIRE Guidelines can be found [here](http://www.squire-statement.org/) (weblink optional reading) (click on “SQUIRE 2.0 Guidelines” in the top menu bar).  It may be unnecessary or inappropriate to include *every single* SQUIRE recommendation in any given published QI project.  However, here are but a few examples of what the guidelines suggest:

1)  Title:  indicate that the manuscript concerns an initiative to improve healthcare

2)  Specific aim(s):  include the purpose of the project and of your report (as I drill into the PGY3s as part of their QI seminar, any good aim statement should answer “how good?”, “by when?” and “for whom?”)

3)  Analysis:  describe methods for understanding variation within the data, including the effects of time as a variable (e.g., if you are doing a QI project that aims to improve some aspect of clinical care by a cohort of residents, and indeed clinical care measures improve, how can you tell it wasn’t simply the passage of time over an academic year, as residents learn more and more stuff and accumulate more experience, that contributed to the improvement?  there are actually pretty clever ways to address this…)

4)  Results:  describe details of any process measures (are we doing the right preliminary work to get to our actual desired outcome?) and outcome measures (are we getting to where we ultimately want to go?)

5)  Interpretation:  note impact of the project on people and systems (QI is all about systems, eh?)

6)  Conclusions:  describe sustainability and potential for spread to other contexts

I recently came across a journal [article](https://www.ncbi.nlm.nih.gov/pubmed/?myncbishare=uwisclib&otool=uwisclib&cmd=Search&term=%09Explanation+and+elaboration+of+the+SQUIRE+(Standards+for+Quality+Improvement+Reporting+Excellence)+Guidelines,+V.2.0:+examples+of+SQUIRE+elements+in+the+healthcare+improvement+literature&submit2=Go) (weblink optional reading) that provided specific examples from the published QI literature of QI research reports that did a great job of following the SQUIRE guidelines.  If you are looking to publish your (current or future) QI projects, this is a really helpful read!  I highly recommend it!

Dear Residents,

**This Week’s Fact:  APA’s new practice guidelines on alcohol use disorder**

For those of you who receive APA [Psych News alerts](http://alert.psychnews.org/2018/01/apa-releases-new-practice-guideline-on.html) (weblink optional reading) in your inboxes, you probably saw this recently.  The APA just published [new practice guidelines](https://psychiatryonline.org/doi/book/10.1176/appi.books.9781615371969) (weblink optional reading) for alcohol use disorder.  Practice guidelines can be a sort of QI initiative, as they aim to improve the standard/quality of care provided to a patient population with a given condition.  This one is primarily a psychopharm guideline.  Its highlights include:

-Naltrexone and acamprosate have the best available evidence related to their benefits, and both have relatively few side effects.  Thus, they should be considered first-line options for patients with moderate to severe alcohol use disorder wanting/needing to reduce drinking or achieve sobriety.

-Disulfiram, gabapentin, and topiramate are also options to be considered, typically after trying the above 2 agents, unless the patient strongly prefers one of these.  Disulfiram, of course, is only an option if complete sobriety is the goal.

One stat from the APA’s [press release](https://www.psychiatry.org/newsroom/news-releases/apa-releases-new-practice-guideline-on-treatment-of-alcohol-use-disorder) (weblink optional reading) that surprised me was this:  the estimated lifetime prevalence rate for alcohol use disorder in the U.S. is 29%.  But <10% of those receive treatment in any given year.  I shouldn’t be surprised by that whopping 29% number, given the staggering rates of alcohol problems I see at University Health Services, yet I am.  Indeed, a practice guideline for something as unfortunately common as this is in order.

Dear Residents,

**This Week’s Fact:  Follow up on physician wellness and QI/patient safety**

Last week, I updated you on the discussions we had with PsychSIG regarding wellness, and some tie-in with QI/patient safety.  Art commented to me later in follow up that AHRQ (Agency for Healthcare Research and Quality—big name in the QI world) has also [made explicit](https://www.ahrq.gov/professionals/clinicians-providers/ahrq-works/burnout/index.html) the link between wellness and quality of care.

AHRQ notes top causes of clinician burnout as follows (no particular order):

1) time pressure

2) chaotic environment

3) low control of pace

4) EHR (as we’ve discussed here before, it’s really become not an option for docs to not adopt EHRs, but it’s hard to implement an EHR without increasing stress/burnout; studies show that practices that implement EHRs see an increase in stress as EHR use “matures” and then a decrease, but never a return to baseline)

5) family responsibilities

AHRQ also lists initiatives, as reported in a variety of studies, found to decrease burnout (and I fully acknowledge that many of these simply aren’t immediately practical as US healthcare stands now):

1) reducing patient panel size

2) increasing flexibility for longer patient visits

3) reducing number of face-to-face visits per day

4) increasing care team staffing

5) creating standing order sets (is there more of a place for this in psychiatry?  many of these studies focus on primary care…)

6) providing responsive IT support

7) reducing required activities

8) providing time in the workday and workflow to complete required documentation tasks and enter data into the EHR

9) offering flexible or part-time work schedules

10) having leaders model and support work-home balance

11) hiring floating clinicians to cover unexpected leave (as with most of these items, easier said than done, but this one seems particularly challenging to implement...)

12) building workplace teams that address work flow and quality measures

13) ensuring values align between clinicians and leaders

So, just a few simple fixes, eh?

Dear Residents,

**This Week’s Fact:  Comprehensive care of pain as a QI initiative**

A few  weeks back, I shared with you an exercise that we (most of our PGY1s and I) did during the recent PGY1 Patient Safety Orientation.  We discussed the issue of co-prescription of opioids and benzos, and EHR initiatives per recent research studies to try to prevent that co-prescription.  Incidentally, if you want to discuss this issue further, talk to James Lehman, who followed up with me afterwards regarding some work he did related to this issue when he was completing his MPH.  This week, I came across [an article](https://www.ncbi.nlm.nih.gov/pubmed/?myncbishare=uwisclib&otool=uwisclib&cmd=Search&term=Comprehensive+care+of+pain:+Developing+systems+and+tools+to+improve+patient+care+and+resident+education&submit2=Go) (weblink optional reading) related to this issue that I wanted to share.  It’s titled “comprehensive care of pain: developing systems and tools to improve patient care and resident education.”  It’s from the primary care residency world, as so many of these articles are, but bears relevance for us.  (And side note:  keep publishing and presenting your PSYCHIATRY-based QI studies, because the literature is still sorely in need of QI studies published by psychiatrists!).

In a nutshell, the article describes a fam med residency program (University of North Dakota) that undertook a massive curriculum and clinical practice overhaul to do their part to stem the opioid crisis.  Their 4-part philosophy in doing so seemed really well-articulated:

1)  all patients deserve to be treated in a respectful and safe manner (side note: do you ever preface a first prescription for a controlled substance with a patient with something like “I’m sorry that it might seem that the system treats you as a criminal for having one of these prescriptions…”?)

2)  patient outcomes are important (i.e., using functional goals instead of acute pain relief makes sense)

3)  provider satisfaction is important (e.g.., a universal/systems-based approach can reduce stress for providers)

4)  clinic flow (phone calls, documentation, emergencies, etc) is important

So, their initiatives included (and I ask:  are any of these relevant to the world of psychiatry, say, if you were tasked with setting up a new approach to benzo/stimulant prescriptions in your future workplace?):

-all patients on opioid therapy for longer than 3 months must participate in a high-risk pain management program.  This includes:  utox is administered at intake, at any signs of aberrant behavior, and at least annually; patients must immediately notify their team if they take more medications than prescribed or receive opioids from another prescriber; and internet resources and a pain class are used to promote self-management

-didactic sessions were developed to include how to have difficult conversations, using communication scripts, and a session for residents to enroll in the North Dakota Prescription Drug Monitoring Program (PDMP) website

Here’s a quote from the article that stands out in my mind, as it is highly relevant for the controlled substances we prescribe as well:  “It is easier in the short run to ‘put out the fire,’ give patients what they request and bypass many of the opioid safety recommendations.  Unfortunately, while this may be ‘usual practice,’ it is not a safe or effective method for managing chronic non-cancer pain.”  Indeed.  And so very analogous  to our world of benzos/stimulants.  We rarely know the full story when patients come to us, from prior prescribers, on incredible amounts/doses of multiple controlled substances.  That said, I have to assume some (definitely not all) of this relates to individual providers, one after another for a given patient, ‘putting out the fire’ with a short-term perspective on things.  We wouldn’t want docs simply ‘putting out the fire’ with our owned loved ones, and so we need to do better than that.  I know each of you do, because you receive awesome training.  There are a lot of psychiatrists out there who didn’t have the benefit of great training.  Go forth and model your reasonable/safe approach to prescribing.

Dear Residents,

**This Week’s Fact:  Why is US healthcare so expensive?**

What constitutes “quality” health care?  It depends on who you ask, of course!  Some of the possible measures to consider in determining quality include:

1)  actual health outcomes (e.g., are the PHQ9 scores of our patients with depression decreasing as a result of their treatment?  and more importantly, are our patients with depression functioning better in life as a result of their treatment?)

2)  access to care (oh indeed, this is a huge problem in psychiatry)

3)  cost effectiveness (or just cost) of care

So yes, cost does matter.  If/as the cost of some services go up, sometimes access to other services gets cut, or maybe access to the newly-more-expensive services gets worse as insurance companies limit access more readily.  So, there are obvious reasons to care about cost that directly relate to quality.  Along the lines of cost, there recently was a lot of attention paid to a [JAMA article](https://jamanetwork.com/journals/jama/fullarticle/2674671) (weblink optional reading) examining the question of why health care spending in the United States is so much greater than in other high-income countries.  For example, in 2016, the U.S. spent nearly twice as much as 10 high-income countries on medical care and performed less well on many population health outcomes.  You’ve heard it before, right?  However, if you read this recent article, you know that the “usual suspects” were NOT found to be the causes of the high costs of our nation’s health care.  That is, compared to other wealthy countries with cheaper care, the U.S. is NOT guilty of the following:

1) more frequent hospitalizations

2) having more specialty physicians compared to primary care physicians

3) more frequent outpatient physician visits

4) spending differently on social services outside of health care, e.g., housing and education (as per prior *Facts*, there are obvious implications for health when considering these social determinants)

What then, is the difference?  It might be these two areas in which the U.S. really stands out:

1)  we pay substantially higher prices for medical services, including hospitalizations, doctor visits, and prescription drugs

2)  our complex payment system causes us to spend far more on administrative costs

Additionally, the U.S. does more imaging studies and more operations of some types (e.g., certain orthopedic surgeries, C-sections, etc.)

So, there you go.  Because I have been seeing this highlighted in a number of places recently, I wanted to make sure you had seen it too.

May we all go forth and provide high quality, cost-effective care, and may our nation provide us a health care system that facilitates that.

Dear Residents,

**This Week’s Fact:  Prior authorizations**

Prior authorizations (PAs) are annoying and time-consuming.  The bottom line intention behind them, it would seem, is to save money for the insurance companies.  And perhaps there is some big picture utility in them.  For example, given what we know about how expensive U.S. health care is (see last week’s *Fact*!), there might well be a place for making sure that Fetzima isn’t the first line choice of antidepressants for patients with a new, first episode of depression, and who have never tried a standard SSRI or other more reasonably priced antidepressants that might be just as good.  That said, PAs are often non-value-added time spent at the expense of more important direct patient care.  I would assume that most people feel that PAs do NOT contribute to quality care.  If anything, they may detract from it.  Either way, there are implications for quality of care.  Here are some (unfortunate) recent [factoids](https://www.ama-assn.org/sites/default/files/media-browser/public/arc/prior-auth-2017.pdf) (weblink optional reading) related to PAs, published by the AMA, that I came across:

1)  Medical practices (across all specialties) spend an average of two business days a week per physician to comply with insurance companies’ PA protocols

2)  1/3 of medical practicals employ staffers who spend every second of their working hours on PA requests and follow-ups (i.e., they are hired solely for the purpose of completing PAs—how fun would that job be?)

3)  How long do docs have to wait for PA decisions from insurance companies?  64% have to wait >1 business day, and 30% report waiting > 3 business days

4)  92% of docs report that the PA process delays access to necessary care

5)  92% of docs report that the PA process has a negative impact on patient clinical outcomes (i.e., this clearly is a QI issue, as discussed above)

6)  86% of docs report that PA burdens have increased over the past 5 years

7)  79% of docs are required to repeat PAs for prescription meds when a patient is stabilized on a treatment regimen for a chronic condition (particularly annoying, I think.  So, for example, you might have received approval for the patient’s aripiprazole at time 0, but then need to repeat that PA at time 1, time 2, and time 3, even though there were no changes in the prescription or patient circumstances)

8)  On average, a medical practice will complete 29.1 PA requests per physician per week

9)  For an increasing number of conditions, even when there are several treatment options, insurance plans are requiring PAs for every single option

So, recall what I said in last week’s *Fact* about about one of the biggest theories about why U.S. health care is so expensive:  administrative costs.  Maybe PAs (and all the staff time and insurance company review time) spent on them might be a part of that administrative burden, eh?  Here’s a [quote](https://wire.ama-assn.org/practice-management/prior-authorization-major-practice-burden-how-do-you-compare?utm_source=BulletinHealthCare&utm_medium=email&utm_term=041418&utm_content=physicians&utm_campaign=article_alert-morning_rounds_weekend) (weblink optional reading) that captures recent trends re: PAs:  “Physicians have, for many years, expected to face prior-authorization hurdles ***for a few new or unusually expensive*** (emphasis mine) medications or tests.  But, more recently, insurers have rapidly added PA requirements to more and more treatments."

Dear Residents,

**This Week’s Fact:  Quality and variability in prescription instructions**

I think many/most agree that electronic prescribing has yielded unequivocal improvement in outpatient medication safety.  However, it’s not without risks.  There’s that ol’ central QI premise that unexplained variation in processes of care are red flags for variable quality.  In other words, if there is variation, we can usually assume that means that some providers/systems are doing things the optimal way, and others aren’t.  So how does this apply to electronic prescribing?  Well, EMRs differ widely, and that was the underlying context in which a recent [study](https://www.jmcp.org/doi/10.18553/jmcp.2018.17404) (weblink optional reading) of 25,000 prescriptions sent to retail pharmacy chains was undertaken.  The study looked at variation in the Sig line (i.e., prescriber instructions for a how a patient should use a medication).  The 501 separate electronic prescribing systems generated 832 different ways to provide the simple instruction:  “Take 1 tablet by mouth daily”.  Why this matters:

1)  About 10% of prescriptions posed a potential safety hazard, based on instruction wording they used, in this study (and that was a SIMPLE patient Sig they studied).

2)  Explicit, standardized instructions improve adherence and patient understanding of how they should take a med.  For example, [research](https://www.ahrq.gov/professionals/quality-patient-safety/pharmhealthlit/prescriptionmed-instr.html) (weblink optional reading) has shown that more explicit prescription medication instructions are better understood than instructions that are vague or require a patient to calculate when to take a medication (e.g., “morning and bedtime” are better understood than “twice daily”).  (Incidentally, this makes me reflect on how I prescribe buspirone.  I usually write “twice daily” on the bottle, to give patients a bit more flexibility (i.e., if that second dose is easier to take at 2 pm, or 4 pm, or 6 pm, I want them to have the flexibility to take it at the time they are most likely to actually do so, but maybe in giving those vague instructions, I’m actually compromising adherence/clarity….).

3)  Pharmacists sometimes put labels/stickers on our patients’ bill bottles, unbeknownst to us, and patients don’t always understand these stickers (which may or may not conflict with instructions we would give).  How clear are [these](https://www.nap.edu/read/12077/chapter/3) (weblink optional reading) labels/stickers?:

“Take with food”

“Do not chew or crush, swallow whole” (as a stickler for proper grammar, what the heck is with the choice of a comma for punctuation there?)

“Medication should be taken with plenty of water”

“Do not drink alcoholic beverages when taking this medication”

“For external use only”

“You should avoid prolonged or excessive exposure to direct and/or artificial sunlight while taking this medication”

So, there you go.  Food for thought, and maybe the makings of future QI projects...

Dear Residents,

**This Week’s Fact:  follow up on quality and variability in prescription instructions**

Thank you for your interest and follow ups with me regarding last week’s *Fact*, regarding variability in how prescription instructions are worded/provided on pill bottles to our patients.  Art astutely asked what some tangible recommendations in this regard might be.  Many of the suggestions I’ve read relate to how pharmacists convey this information (e.g., what stickers they choose to put on pill bottles, what size font they choose to use, and how they ‘interpret’ our instructions when we write/dictate them in medical-speak).  However, there are some suggestions for us, the prescribers, as well.  Here you go:

1.  Use explicit text to describe interval between doses in instructions (I hinted at this one last week, i.e., specific times such as “morning and bedtime" would be preferable to “twice daily”).

2.  Simplify language as much as possible, avoiding unfamiliar words and medical jargon (pharmacists may or may not choose to appropriately simplify our language, so we should do it ourselves).

3.  When possible, include indication for use (this can take us back to the stigma issue, and if this would be embarrassing for patients, but ultimately, if it is a patient safety issue—and it is—then being clear on the prescription instructions should generally take priority over concerns about embarrassment).

4.  When possible, use numeric instead of alphabetic characters (so “2 tabs” instead of “two tabs”).

So, these are things we can do on an individual basis.  QI interventions, though, of course, would involve systems changes that make it all but impossible for us NOT to word our prescriptions in the clearest way possible.  And a complicating factor is that, understandably, prescribers want to retain some autonomy and flexibility in how we word things for our patients.  Just a simple QI project, eh?

Dear Residents,

**This Week’s Fact:  Best Practice guidelines as a QI tool**

Before we dive in to this week’s *Fact*, I wanted to remind you that today at the HSLC is the UW Health Resident Quality and Safety Council Symposium!  So, maybe I’ll see some of you in the HSLC Atrium this morning to check out the QI posters from trainees across the institution.  Then, the keynote presentation is 12-1:00, titled “Quality measurement in health care”, by Dr. John Scarborough.

Back to the *Fact* at hand:  As you know, our residents spend time during their PGY3 year working on QI projects.  When I meet with them at the beginning of their QI rotation to discuss strategies for their QI projects, residents often aren’t sure what they should do for a QI project.  Central tenets that I share with them regarding how to come up with good ideas for change include the following:

1) focus on a solution that deals with the *root cause* of the problem

2) eliminate waste (what doesn’t add value in a given clinical process?)

3) use technology (e.g., automating a process via changes in EMR templates so that ‘doing the right thing’ doesn’t rely on human memory, which is inherently fallible)

4) use benchmarking and best practices (e.g., see what others have done, call other clinics in town to find out their practices, go to the literature, or look at best practice guidelines)

Along the lines of #4, then, I always take note when I see that some new practice guideline/best practice has been published.  And indeed, the APA and American Telemedicine Association just released a best practice [document](https://www.psychiatry.org/File%20Library/Psychiatrists/Practice/Telepsychiatry/APA-ATA-Best-Practices-in-Videoconferencing-Based-Telemental-Health.pdf) (weblink optional reading) on interactive videoconferencing with patients.  It broadly includes 3 categories of considerations when it comes to videoconferencing-based telemental health services:

1)  administrative considerations (e.g., info on legal and regulatory issues including licensure and prescribing.  For example, did you know that typically you must be licensed in the state in which the patient is physically located in order to provide telepsychiatry to that patient?)

2)  technical considerations (e.g., HIPAA and state privacy requirements and appropriate room set-up for telemental health sessions.  For example, the guidelines states that the patient and provider cameras should be placed at the same elevation as the eyes with the face clearly visible to the other person.)

3)  clinical considerations (e.g., patient and setting selection, ethical issues, and factors to consider when treating specific populations such as children.  For example, the guidelines state that when working with younger children, the environment should facilitate the assessment by providing an adequate room size, furniture arrangement, toys, and activities that allow the youth to engage with the accompanying parent, presenter, and provider and demonstrate age-appropriate skills.)

So, this is food for thought as you potentially do telemental health rotations through the residency, or consider telemental health jobs post-graduation.

Dear Residents,

**This Week’s Fact:  Featured aspect of our QI curriculum**

If you are a PGY-3 or -4, you are pretty well acquainted with all aspects of our QI curriculum.  However, underclass residents have only been exposed to some parts of it.  Thus, I like to feature an interesting aspect of the curriculum from time to time so that everyone has a big picture understanding of the kinds of things our curriculum covers.  This week, I introduce y’all to the PIP modules that our PGY3s complete.  What is a PIP module, you ask?  PIP module=Performance in Practice module.  Some context:

It used to be that maintaining your Board certification in psychiatry (or any specialty) was relatively simple.  You take your Board exam shortly after finishing residency, and then take a certain number of CME credits (e.g., by going to Grand Rounds, conferences, etc), in whatever topics you please, every certain amount of time.  It’s not so simple anymore, and it involves QI, and that is why the PIP modules are part of the QI curriculum.  As part of the QI curriculum, we have residents do these PIP modules, which are essentially mini personal QI projects that exactly replicate what you’ll have to do in the post-residency world.  The idea is to have you do this in the supported confines of a residency program, so you know exactly what to do once you are out there.  I can tell you that many of my younger colleagues in my UHS clinic are quite scared of the QI requirement for board certification.  Our PIP requirement demystifies the requirement.

In sum, to do a PIP project, residents use one of the PIP modules that APA has produced.  They use the module to guide their review of 5 of their own patient charts, identify and implement an improvement plan based on those charts, and then re-assess another 5 charts to see if they improved some aspect of care.  The aspect of care might involve how well they assess safety, how well they assess measures of depression, or something else.  An alternative approach is to give feedback forms to 5 patients, peers, or 360 degree (e.g., RN) evaluators.  Then, they identify and implement an improvement plan based on that feedback, and finally, they re-asses via another 5 feedback forms.

Hopefully it doesn’t seem too scary, and maybe even a little fun.

Dear Residents,

**This Week’s Fact:  M&M**

Do you think of our Department’s M&M conferences as exercises in QI?  Indeed, they are.  They are designed to specifically focus on systems issues (NOT individual provider issues—there would be no point in assembling the whole Department just to air one person’s ‘mistakes’, and in fact, cases are specifically chosen that do NOT involve any perceived significant individual errors, and rather, focus on opportunities for systems improvements).  Since M&Ms are QI-related, our QI curriculum involves having each PGY3 present an M&M case (both in the actual Friday M&M, and at the pre-M&M meeting on the preceding Wednesday, wherein our Department’s M&M Committee plans for how to present the case with the whole Department assembled).  As many of you have seen, this has been a wonderful opportunity to involve our residents in these critical discussions, and to give them leadership and experience in helping to facilitate these types of activities.  In fact, it has become an ACGME Level 4 (“graduation ready”) Milestone (Systems Based Practice 1 Milestone 4.2/C—“Develops content for and facilitates a patient safety presentation or conference focusing on systems-based errors in patient care”) for psychiatry residents.  Many U.S. psychiatry departments do not actually offer psychiatry M&Ms, so we are uniquely able to have our residents meaningfully accomplish this Milestone.  And you have truly done a stand up job in doing so.

Along these lines, I just came across a recent review [article](https://www.ncbi.nlm.nih.gov/pubmed/?myncbishare=uwisclib&otool=uwisclib&cmd=Search&term=%09The+role+of+morbidity+and+mortality+rounds+in+medical+education:+a+scoping+review&submit2=Go) (weblink optional reading) on M&Ms in medical education.  Some interesting points highlighted in this article include:

- M&Ms have the potential to create opportunities for QI projects that can be led by residents.  Indeed, when our PGY3s start their QI rotation, I share with them a list of suggested QI projects, as provided by faculty members at each site who are involved in QI initiatives.  The list of WisPIC-based QI project suggestions includes ideas that stem directly from our M&Ms.
- QI initiatives are more likely to be realized/implemented as a result of M&M conferences if the following occur during M&M:  cases are analyzed in a systematic, QI-informed manner; members of an interprofessional team are invited to collaboratively discuss the case (i.e., it’s not just psychiatry residents/psychiatrists discussing the case, but rather, the discussions include psychologists, social workers, RNs, etc); and a trained moderator is present to lead an organized discussion.  Thanks to Dr. Garlow, our residents, and our M&M Committee, I think all those boxes are checked.  The trained moderator (Dr. Garlow) partnering with our resident case presenter has seemed, IMHO, a very nice way to do this.
- Only 2 studies have ever attempted to look at whether M&Ms ultimately lead to improved patient care.  Of course, that is the hope we have for M&Ms, but it has simply not been studied (food for thought in case any of you are ever interested in doing that sort of study, which I imagine would be quite challenging).  For what it’s worth, I personally feel that I change my practice in some way, big or small, after every single M&M.  I have found them truly transformative.

Do you have other thoughts on our M&Ms?  Feel free to drop me a line!

Dear Residents,

**This Week’s Fact:  More on QI initiatives to address the opioid epidemic**

I recently attended the 2018 Annual Meeting of the American Medical Association House of Delegates.  To say the least, the opioid epidemic was a widely discussed topic.  We all know of ePDMP initiatives to target the opioid epidemic, but a number of other QI/systems initiatives are being discussed too.  One of interest to me has been the issue of prescription of naloxone to patients who may be at risk of opioid overdose.  On the one hand, you might view this as as individual patient issue and not a QI/systems issue.  That is, physicians might decide on an individual patient-by-patient basis who should receive a naloxone prescription.  On the other hand, I submit that there is a place for system-wide initiatives and overarching clinical practice recommendations within the world of medicine.  For example, consider the following:

1)  Are there general guidelines that could be offered for psychiatrists specifically to determine for whom/when we might reasonably prescribe naloxone?  Would it be our place to do so if we are (with extreme caution, and on an exceptionally rare basis if ever) prescribing a benzodiazepine to someone who is also prescribed an opioid by one of their other physicians?  Would it be our place to do so if we are not prescribing benzos, but we have reason to be concerned that our patient’s mental illness puts them at greater risk of OD on the opioid prescribed by one of their other physicians?  Our patient population, compared to others, disproportionately takes opioids, as a fairly recent [study](https://www.ncbi.nlm.nih.gov/pubmed/28720623) (weblink optional reading) demonstrated that adults with mental disorders receive >50% of all opioid prescriptions distributed each year in the U.S. (!).

2)  One example of a systems-based approach to this issue is that of a physician writing a ‘[standing prescription](https://host.madison.com/ct/news/local/education/university/uw-campus-walgreens-to-dispense-heroin-antidote-naloxone/article_ae6cd465-075b-5896-9fc5-f65910dce026.html)’ (weblink optional reading) for a pharmacy for naloxone, such that anyone in the community can go to that pharmacy and request that the prescription be filled.  In fact, one of the psychiatrists at University Health Services on the UW-Madison campus has, under her name, a standing prescription for naloxone at the Walgreens right downstairs from our clinic.  The prescription can be filled by anyone (doesn’t need to be a UW student) via simple request without an individual prescription, as allowable per a 2015 Wisconsin state law that allows standing naloxone prescriptions in this manner.  It can be filled for possible personal use or for use in the event a family or friend overdoses, and the dispensing pharmacist provides instruction in how to administer the drug.

More info on the topic of naloxone prescription is [here](https://www.aafp.org/dam/AAFP/documents/patient_care/pain_management/co-branded-naloxone.pdf) (weblink optional reading).

Dear Residents,

**This Week’s Fact:  How to fix the EMR problem**

Physicians having to finish their clinical documentation well into their evening hours is a big problem.  Physician burnout is a big problem.  The former might be one issue that contributes to the latter.  So, systems fixes are needed.  I’ve highlighted the issue before. A new [report](https://wire.ama-assn.org/practice-management/simpler-logins-voice-recognition-ease-click-fatigue-yale?utm_source=BulletinHealthCare&utm_medium=email&utm_term=052618&utm_content=physicians&utm_campaign=article_alert-morning_rounds_weekend) (weblink optional reading) that I read, based on Yale’s experience, highlights 3 big potential solutions:

1)  Eliminate repetitive typing of the physician’s username and password.  Instead, physicians can use their badges to tap in and and out of the system throughout the day after a one-time login at the start of their shift.  This may save on 20-140 logins per physician per day.

2)  Use speech recognition software.  This may cut the time to complete/close encounters in half.

3)  Use virtual scribes.

Another recent [report](https://wire.ama-assn.org/practice-management/peer-led-ehr-training-saves-physicians-scarcest-resource-time?utm_source=BulletinHealthCare&utm_medium=email&utm_term=061618&utm_content=physicians&utm_campaign=article_alert-morning_rounds_weekend) (weblink optional reading) showed that peer-led EMR training can be high yield.  It allows for rapid sharing of best practices and efficiencies that our peers have developed, and that haven’t necessarily occurred to us.  Obviously any of these initiatives would be easier said than done, but from a QI perspective, I really appreciate looks at these things that optimize efficiency, remove non-value-added steps to provision of care, and ultimately may even improve care.

On a final note, one more strategy that could be considered is to start training in how to use an EMR at a very, very young age, so that by the time folks are practicing physicians, it is second nature.  See attached for my 2 year old working on this sort of QI initiative at a recent meeting that the little ones attended with me.

Dear Residents,

**This Week’s Fact:  Another possible pathway to prevention of and recovery from physician burnout**

Several times in this column I have addressed the issue of physician burnout, essentially as a QI issue.  We can’t provide quality care if we are burning out, eh?  So many people have ideas about how to fix the burnout problem, of course, but I thought one idea that I just read was noteworthy and interesting...

Specifically, an [article](https://jamanetwork.com/journals/jamapsychiatry/article-abstract/2680572) (weblink optional reading) in the July issue of JAMA Psychiatry discusses the issue of the gradual loss of physician autonomy, over past years, as a driver of burnout.  This has perhaps paralleled the move from many/most docs working in solo or small group private practices, to being employed in large health systems.  I for one have lots of positives to say about not being in private practice, as I have little desire to try to oversee billing practices in a private group, or to figure out how to advertise, or to figure out how to get on insurance panels (those are the kinds of things that might burn me out).  That said, the article points out the downside to physician employment being the loss of autonomy, specifically involving less control over how much time to spend with patients, what treatments can be offered, what tests can be performed, how encounters must be documented, and a whole host of other mandated regulations, all while having to meet certain performance expectations imposed by insurance companies, government agencies, etc.  The result might be a sense of overwhelm and powerlessness.

I do believe we know from our psychiatric knowledge base that the degree of control a human can exert over a stressor has a notable impact on the emotional, behavioral, neurobiological, and physiological effects of that stressor.  Remember the whole theory of learned helplessness as a cause of depression?

What might be the answer, then, for employed physicians who don’t wish to burn out?  The article suggests benefit from a leadership model in which physicians are encouraged to actively participate in governing and improving the systems in which they work.  Perhaps the most overused phrase I hear at various policy-making meetings (e.g., AMA) that I attend is “If you’re not at the table, you’re on the menu.”  Maybe that applies here?  For some of us, involvement in not only local institutional administrative or leadership roles, but also professional medical societies, provides some sense of being able to influence change over otherwise seemingly insurmountable barriers that exist in health care systems.  Criticize AMA and APA if you will (certainly neither is beyond criticism), but I do leave policy-making meetings of those orgs with a renewed sense that indeed we might just be able to take steps to fix the  health care system.

Just something to consider as you look at future job opportunities (be it in private practice, employed large groups, or otherwise) and as you consider future professional involvement...

Dear Residents,

**This week’s *Fact*:  5 reasons physicians are less likely to seek support for burnout**

This topic of burnout continues to prove ripe for QI *Facts*.  An [article](https://wire.ama-assn.org/life-career/5-reasons-physicians-are-less-likely-seek-support?utm_source=BulletinHealthCare&utm_medium=email&utm_term=080418&utm_content=physicians&utm_campaign=article_alert-morning_rounds_weekend) (weblink optional reading) I just read seemed particularly appropriate for a *Fact* feature, given the infusion of some other QI-related tidbits that I’ve been wanting to pass along at some point anyways.

The article points out that physician well-being is increasingly recognized as the FOURTH goal that joins the TRIPLE AIM of health care.  I don’t believe I've mentioned the triple aim in a couple of years in this column, so it’s overdue.  The triple aim of health care is an important QI concept and consists of:

1) improving health care quality

2) improving patient experience

3) lowering health care costs

In general, QI initiatives do well to consider how their project might impact all 3 of those aims.  Additionally, it is, I would argue, paramount to consider the 4th goal of how any such project might impact physician well-being. For example, it might be all good and well to try to improve health care by asking docs to consider 18 new factors in each of their new patient intakes, but if they all burn out and leave the profession, we have lost the forest for the trees.

Anyways, that was the introductory part of the article.  The meat of it, really, was its description of 5 reasons physicians are less likely to seek treatment for burnout (and hence, why attention to that 4th aim of physician well-being is critical).  These 5 reasons are:

1)  **Fear of licensure problems**.  We as physicians encourage our patients to share their concerns about depression, anxiety, etc, yet are less likely to seek help for ourselves for these problems.  Part of the reason historically relates to a history of mental illness potentially making it harder for docs to obtain licensure.  Rest assured that in recent years, Wisconsin has taken admirable steps so that our licensure application wording is imminently more reasonable than it used to be (i.e., now it asks if you have a medical, including psychiatric, condition that is currently impairing your ability to do your work).  That said, some states still live in the stone age and ask all applicants to indicate if they’ve ever had a mental illness, and if they check yes, they may have to go before board authorities for questioning.  Seriously.  The highlight of my most recent AMA House of Delegates meeting was being able to testify on behalf of the American Psychiatric Association in support of the AMA “encouraging state licensing boards to require disclosure of physical or mental health conditions only when a physician is suffering from any condition that currently impairs his or her judgment or that would otherwise adversely affect his or her ability to practice medicine in a competent, ethical and professional manner, or when the physician presents a public health danger.”  Thankfully that did pass as official AMA  policy, though I’m embarrassed to report that there was some opposition to it, with the sentiment that it “goes too far” and “doesn’t do enough to protect the public from mentally ill doctors”.  I seriously almost choked on my coffee when I heard that bit of stigma-laden testimony.

2)  **The “physician personality”**.  The typical doc who is thorough, committed, and ‘leaves no stone unturned’ is not only potentially more prone to burnout, but may be less apt to admit there is a burnout problem at hand.

3)  **Programmed to cope alone**.  In recent times, docs have busier schedules, higher productivity expectations, and more documentation time, leaving less time for interaction with colleagues.  Consequently, physicians are more likely to handle stress alone.

4)  **A survival mentality**.  Throughout med school and residency, there is a survival mentality.  “I’ve just got to make it through.  Things will get better when I’m done with training.”  Except that physicians perpetuate that same thought process throughout their entire careers.  37% of docs report looking forward to retirement as an effective strategy for well-being.

5)  **Self-doubt/imposter syndrome**.  There is a level of self-doubt that permeates many physicians.  Many are waiting to be discovered as the fraud they think they are, so they stay quiet about their insecurities.

Dear Residents,

**This week’s *Fact*:  Post-hospital syndrome**

This week’s *Fact*isn’t specifically just about psychiatry, but I find it interesting and compelling.  Have you heard the phrase “post-hospital syndrome”?  It was [coined](https://www.nejm.org/doi/full/10.1056/NEJMp1212324) (weblink optional reading) in 2013, and refers to a period of generalized risk for a range of adverse health events approximately one month after a hospitalization.  The risk reportedly not only stems from ongoing impact of the original medical condition for which patients were hospitalized, but also from impacts of the stress that patients experience in the hospital.  Medicine has steadily improved its care of acute medical conditions per se, but sometimes, perhaps, the forest may be lost for the trees.  It is compelling that the severity of an original acute illness poorly predicts which patients will require readmission.  Hospital-related stressors may include:  sleep deprivation (all those middle-of-the-night blood draws, beeping machines, etc); disruption of normal circadian rhythms; poor nourishment (e.g., via frequent NPO orders, with cancelled and rescheduled tests prolonging these NPO periods); pain and discomfort; “confronting a baffling array of mentally challenging situations”; receiving meds that can alter cognition and physical function (apropos of this, Dr. Langheim gave a terrific PGY3 QI seminar 2 days ago, and in that context mentioned his work on improving hospital-based protocols in a certain non-UW health system, and in that capacity his dismay to find that qhs benadryl was part of the standard order set for older patients admitted with hip fractures); and deconditioning owing to bed rest/inactivity.

This syndrome is the kind of thing that makes me fear for my own loved ones needing to be hospitalized, and that fuels my interest in QI.  Clearly this needs system-level intervention.  What are possible QI interventions, then?  These are some proposals (just simple options for a PGY3 QI project, eh?) that people have floated:

-screen for functional disabilities, including both cognitive and physical ones, at the time of discharge

-implement risk-mitigation strategies to protect against falls at home

-minimize disruptions in sleep

-promote good hospital nutrition and address nutritional deficiencies

-ensure sedative use is appropriate

-promote practices that reduce the risk of delirium

-emphasize physical activity and strength maintenance or improvement

A recent [article](https://www.nytimes.com/2018/08/03/health/post-hospital-syndrome-elderly.html) (weblink optional reading) in the New York Times highlights one family’s personal experience with post-hospital syndrome.  Though obviously just Ns of 1, these personal stories are often highly compelling when it comes to concerns about patient safety.

Dear Residents,

**This week’s *Fact*:  Nudges**

Have you heard the word “nudges” in reference to QI?  I’ve been coming across the term more and more.  A nudge is a concept from behavioral sciences, describing an intervention that alters people’s behavior in a predictable way without forbidding any options or significantly changing their economic incentives.

A recent [article](https://jamanetwork.com/journals/jamasurgery/article-abstract/2688235) (weblink optional reading) in JAMA Surgery provides a great example of an effective nudge.  An intervention was designed to try to decrease the number of opioids prescribed per surgical patient, and thereby ultimately have a favorable impact on the opioid epidemic.  The study describes how Yale lowered the default number of opioid pills on EMR prescriptions from 30 to 12 for patients undergoing the 10 most common outpatient operations.  The end result was that the median number of opioid pills prescribed per operation decreased from 30 to 20 (i.e., physicians could still manually go in and change the new default number of 12, and they did, but not typically back up to the prior default number of 30).  And importantly, patients were no more likely to call in for refills after this change.

The beauty here is in the simplicity of the intervention, and the automation of the intervention (i.e., the physicians did not need to remember to do anything different), and the resulting rapid change in practice.  Another non-psychiatric issue where I’ve seen nudges oft discussed as of late is the issue of organ donation, and how a nudge of creating a national opt-out rather than opt-in policy would lead to significantly highly rates of organ donation.  Are there places for nudges in our own psychiatric practices, either at individual or systems levels of care?

Dear Residents,

**This week’s *Fact*:  Patient safety on inpatient psychiatry units**

You may have noticed that Psychiatric Services (APA’s other journal) just published an interesting QI-related [study](https://ps.psychiatryonline.org/doi/10.1176/appi.ps.201800110) (weblink optional reading) looking at factors linked to adverse events and errors during psychiatric hospitalization.  In my mind, this is a big deal, as, while general medical specialties have oft been publishing on patient safety/QI issues, psychiatry has, shall we say, been lagging behind a bit.  So, what did they find?

First, some definitions/examples based on how the authors described things:

Adverse events:  included self-harm/injury, adverse drug events, assaults, sexual contact, and patient falls (all while on inpatient psych units)

Medical errors:  included any mention in inpatients’ charts of medication errors, elopement, possession of contraband, and “other non-medication errors”

The findings?

Factors that PROTECTED AGAINST adverse events and medical errors were:  private insurance, being treated at HIGH volume hospitals, and being ages 31-42

Factors that conferred HIGHER RISK of adverse events and medical errors were:  age 54 or older (more than 2x as likely to experience an adverse event compared with patients ages 18-30), admitted during the weekend, admitted to a rural hospital, longer length of stay, and treated at a VERY high volume hospital

Take-homes they offered included:

1.  Perhaps there is some ideal balance between hospitals having high enough volumes that they are adequately resourced, but not so high volume that they operate at overcapacity or with higher patient-to-nurse ratios.

2.  Mental health would do well to adopt the "5-step framework" adopted in general medicine to lower rates of preventable harm, as follows:

a.  measure actual rates of errors

b.  develop evidence-based care practices

c.  invest in implementation of patient safety plans

d.  incorporate local hospital ownership and peer learning

e.  align efforts around common goals and measures

My personal take homes:

1.  The 5 steps described above are exactly what our PGY3s do in their QI projects (and so it is interesting that it’s kind of presented as a rocket science sort of idea by the authors, but then again, our PGY3s are on par with rocket scientists..).

2.  Maybe any of our residency’s QI projects that focus on inpatient psych unit patient safety issues might consider the apparently particularly vulnerable populations identified in this study (older patients, those without private insurance, those with longer inpatient stays).

Dear Residents,

**This week’s *Fact:*  Training our primary care colleagues to deliver outstanding depression care**

As part of this residency program, you get the opportunity to participate in an important model of mental health care delivery called integrated care.  Variations of this model of care are an integral part of the Access Community Health Center and VA systems.  UW Health is expanding this model too.  Given the dire shortage of psychiatrists, this is seemingly an important model of care in that it supports our primary care colleagues to be able to provide high quality mental health care, even when there aren’t enough of us to see individual patients on frequent visits within psychiatry clinics per se.

Related to this idea of optimizing the quality of mental health care that our primary care colleagues can provide, a review [article](https://www.ncbi.nlm.nih.gov/pubmed/?myncbishare=uwisclib&otool=uwisclib&cmd=Search&term=%09Elusive+search+for+effective+provider+interventions:+a+systematic+review+of+provider+interventions+to+increase+adherence+to+evidence-based+treatment+for+depression&submit2=Go) (weblink optional reading) was just published titled “Elusive search for effective provider interventions: a systematic review of provider interventions to increase adherence to evidence-based treatment for depression.”  The review intentionally excluded articles that looked at collaborative care as an intervention to train our colleagues in provision of high quality mental health care, as the hope was to find some evidence-based but less resource-intensive ways to train people (typically non-psychiatrist physicians) to provide quality, guideline-based care for depression.

The systematic review notes that a range of provider interventions have been described in a variety of research studies.  These interventions have included, for example:

1) distributing guidelines to providers

2) education/training such as “academic detailing”

3) combinations of education with other components, such as targeting implementation barriers that seem to exist in certain clinics or with certain individual providers

So what did the article find?  What works and what doesn’t?  Alas, the “elusive search” part of the article title doesn’t bode well, eh?  There wasn’t anything hugely compelling in terms of any single type of intervention that statistically significantly improved depression treatment guideline adherence, but the caveat is the huge variability in quality/type of studies, and thus great difficulty pooling results.  And on the bright side, some of the provider interventions were associated with overall more favorable outcomes.  And apart from guideline adherence per se, there was some evidence that educational interventions did improve medication prescribing (if not guideline adherence per se).  So, for the PGY3s who are charged with giving an educational talk on a psychiatric topic to the multidisciplinary team at Access as part of that rotation, take solace knowing that you very well might be helping to optimize quality of care delivered there (anecdotally, I know that to be true from talking to providers there).

Dear Residents,

**This week’s *Fact:*  A simple but lovely med student QI initiative**

QI initiatives do not need to be big, fancy, or expensive.  I love this recent [study](https://www.ncbi.nlm.nih.gov/pubmed/?myncbishare=uwisclib&otool=uwisclib&cmd=Search&term=%09Listening+Beyond+Auscultating:+A+Quality+Initiative+to+Improve+Communication+Scores+in+the+Hospital+Consumer+Assessment+of+Health+Care+Practitioners+and+Systems+Survey&submit2=Go) (weblink optional reading) that describes a med student QI project (they are residents by the time the study was published).  It was basically a group of med students (probably assigned to do a QI project) who were looking to improve patients’ perception of listening by physicians.  They did a lit review to find evidence-based interventions that might improve patients’ perception that their docs are actually listening.  Of the 42 possible interventions they found described in the literature, they determined that 24 might be feasible in their site (inpatient medicine service).  They did small-scale testing (PDCA cycles!), and in so doing determined the 4 most promising interventions.  Additional PDCA cycles via little pilot studies and feedback from patients and docs led them to refinement of the interventions, and choice of a single final intervention.  So what was the final intervention chosen?…..

***Use of a structured reminder embedded in the EHR to direct physicians to begin interventions by eliciting patient concerns.***

As I recall, that is the first thing taught in Med-1 patient interviewing courses, right?  “What brings you in today?”.  But how soon we forget.  We have agendas.  We know what we need to plug and chug in our EHRs.  We have limited amounts of time.  But lo and behold, if we remember that one important question (tailored slightly of course if you are in inpatient versus outpatient settings), patients feel more heard.

And this article provides a great opportunity to remind y’all of differences between QI and research per se.  There certainly is overlap.  But notice how the med students in the above study looked to already-existing research to find evidence-based interventions, but those evidence-based interventions had to be finessed and uniquely tailored to their local site.  The point of QI is typically taking interventions that (hopefully) have some degree of evidence base (or best practice recs) to support them, but then appropriately applying them to our unique local setting.

Dear Residents,

**This week’s *Fact:*Patient respect in health care**

Perceived disrespect within the health care system, on the part of patients, families, and workers themselves, has been associated with a worse patient experience, lower likelihood of perceiving care as high quality, and lower likelihood of seeking care again in the same facility. In addition, there is an association between disrespect and risk of physical harm.  I would say this all counts as a patient safety/QI issue, then.

A  new [report](https://www.jointcommissionjournal.com/article/S1553-7250(17)30554-8/fulltext) (weblink optional reading) out of The Joint Commission Journal on Quality and Patient Safety highlights this problem.  Importantly, they have some recommendations for improving upon the issue.  I’ll list some here:

1. Support health care professionals who experience disrespect from other health care professionals, patients, and/or family members.
2. Engage health care professionals in understanding the connections among respect, dignity, safety, quality, outcomes, and the experience of care.
3. Organizational leaders should partner with patients and families to develop a shared vision of the practice of respect.
4. Since what constitutes respect may vary among patients and families, health care professionals at the point of care must partner with them to learn how best to honor their goals, values, and preferences.
5. As part of the practice of respect, promote health equity by engaging and partnering with individuals and communities that experience disparities.
6. Recognize, celebrate, and learn from respectful behavior and positive experiences (CR:  I really love this one!  What if there were an M&M—though we’d have to call it something else since the name wouldn’t fit--to look at really positive interactions/outcomes, and we essentially did a root cause analysis to see what salient factors led to the positive result?).
7. Embed organizational systems for learning about and improving the practice of respect in operational structures to ensure their success and sustainability (CR: as you’ll recall, the need for sustainability is one of those central tenets I try to emphasize throughout our QI curriculum!).
8. Identify, compile, and share successful strategies at all sites.

Dear Residents,

**This week’s *Fact:* Overuse as a patient safety issue**

A recent NYT [article](https://www.nytimes.com/2018/09/10/upshot/its-hard-for-doctors-to-unlearn-things-thats-costly-for-all-of-us.html) (weblink optional reading) titled “It’s hard for doctors to unlearn things.  That’s costly for all of us.” caught my eye.  It was largely based on a systematic [review](https://jamanetwork.com/journals/jamapediatrics/article-abstract/2676071) (weblink optional reading) from May in JAMA Pediatrics that looked at the literature related to overuse in pediatric care.  The NYT author observed that it can be hard to persuade docs to do some things that have proven benefits (e.g., in our world, perhaps monitoring of metabolic parameters in patients on atypical antipsychotics), but it might be even harder to get them to STOP doing some things.  Examples given from the JAMA Peds article note that in 2016 alone, studies were published that showed that there are still recs given involving kids that appear to represent overuse/overintervention:

-we give antidepressants to kids too often (I’m not child psych, but coming out of a non-psychiatry journal that immediately makes me feel a bit defensive...)

-we recommend that kids consume commercial rehydration drinks (e.g., Pedialyte) when their "drink of choice" would do

-deliveries are induced too early

-ankles are x-rayed often, looking for injuries we almost never find

-we strive for very tight glycemic control in ICU patients based on dated research, despite a landmark 2009 study demonstrating higher mortality with such tight control

Why are docs so, shall we say, SLOW to remove outdated tendencies from their clinical practices?  The above-linked articles make the following speculations:

-even if docs accept new contradictory evidence, they oft work within healthcare systems that don’t adapt well/quickly/at all to changing evidence (this makes me think of how, with our QI curriculum, I really emphasize to residents the need to find some way to ‘automate’ their new initiatives, so that providers really have no choice but to do the right thing.  well…I imagine that same automation would make it hard to reverse initiatives if/when found to be outdated….)

-we need to take a more cautious approach to technology adoption and learn from mistakes of early adoption of health care technologies based on little or low-quality clinical evidence

Have y’all heard of the [Choosing Wisely](http://www.choosingwisely.org/our-mission/) (weblink optional reading) initiative?  This *Fact*reminds me of it.  It’s been a while since I’ve mentioned it in this venue, but in short, Choosing Wisely is an initiative that has its roots in Internal Medicine. It has spread to almost all specialties and is focused on identification of care that physicians recommend but shouldn’t (i.e., it looks at areas of ‘overuse’).  The problem with these areas of overuse is that, on average, they:

1) don’t provide a benefit

2) can lead to harm

3) can cost a lot of $$

To relate this to our own QI curriculum, when the PGY3s are developing their QI projects, we ask that they tie in their projects at least in some small way to a quality initiative of a known/respected entity, and that could include the Choosing Wisely [items for psychiatry](http://www.choosingwisely.org/clinician-lists/#parentSociety=American_Psychiatric_Association) (weblink optional reading).

Back to the NYT article, I think the article really drives home the point that changing physician behavior, not only if you are asking them to do some NEW bit of work, but also (and perhaps especially) if you are asking them NOT to do something they ARE used to doing, can be challenging.  This is why stakeholder engagement and buy-in for our QI projects is key.  Not easy, but key.  Can you think of other areas within psychiatry where overuse is an issue?  And specifically, where reversing on the overuse trend might be a challenge?  That might be material for future QI intervention.

Dear Residents,

**This week’s *Fact:*  “A new paradigm for mental health quality and safety"**

The Australian health care system is presumably rather different than ours.  However, a recent [article](https://www.ncbi.nlm.nih.gov/pubmed/?myncbishare=uwisclib&otool=uwisclib&cmd=Search&term=A+new+paradigm+for+mental-health+quality+and+safety:+are+we+ready?&submit2=Go) (weblink optional reading) from Australia, which looked to develop a framework for quality improvement activities in that geographic area’s psychiatric units, did catch my eye.  There’s more and more published on QI these days, and still vastly little within psychiatry per se, so you have to take what you can get (but this is actually a rather good article).  The researchers came up with important components of QI initiatives within psychiatry (inpatient especially) via review of the literature, local and regional meetings with stakeholders, including patients, families/caregivers, providers, leadership, and data experts, and study of the “current patient safety landscape both locally and internationally” (so there you go—it could well be relevant to psychiatry in Wisconsin!).

These are among the factors that emerged as imperative for QI initiatives in psychiatry:

1)  executive support

2)  staff engagement (recall from a prior *Fact*:  engagement=opposite of burnout in many ways)

3)  starting on small projects and achieving quick wins (what I always say to our PGY3s:  “It’s impossible to pick a QI project that is ‘too small’—it’ll be more challenging than you think, no matter how small!")

4)  celebrating innovation

Aspects of care that emerged as top priorities in terms of areas in which quality of care could be improved from the perspectives of patients and their families/caregivers included:

1)  reducing restraint and seclusion

2)  management of medical comorbidities

3)  management and monitoring of medication side effects

4)  transparent and timely communication at admission, discharge, and following unexpected incidents

5) structured and meaningful activity and/or therapeutic programming

6)  cleanliness of facilities

7)  improved hospital food (I admit we’ve never had a PGY3 QI project focused on hospital food)

8)  better nicotine dependence management (we’ve covered this in several prior PGY3 QI projects)

9)  making patients and carers feel welcomed at inpatient units

10)  involving families/caregivers more (see Rebecca Radue’s QI project on optimization of resident involvement in family meetings from last year)

11)  increasing access to psychological services

12) stigma reduction

Many of those seem to extrapolate to our health care system here in Wisconsin!
