# Supplementary material for: A Quality Improvement Curriculum for Psychiatry Residents
Source: MedEdPORTAL. 2020 Jan 24;16:10870. doi: 10.15766/mep_2374-8265.10870 (PMC7012317; doi:10.15766/mep_2374-8265.10870)
Supplement: Supplementary file 1 — A. QI Didactic Seminars.doc B. Introduction to the QI Rotation Slides.ppt C. Essential QI Toolbag Slides.ppt D. Patient Safety Slides.ppt E. Principles of Survey Design Slides.pptx F. CBC and PIP Modules Slides.pptx G. Involving Stakeholders Slides.ppt H. QIKAT for Psychiatry.doc I. QI Workbook.doc J. QI Final Presentation Guidelines.doc K. A3 QI Poster Template 11x17.pptx L. QI Supervisor Evaluation of Resident.docx M. QI Director Evaluation of Resident.pdf N. QI Facts of the Week Sample.docx [file mep-16-10870-s001.zip › A. QI Didactic Seminars.doc]

**PGY-3 Quality Improvement Didactic Curriculum**

| **Date** | **Topic** | **Readings/homework to assign before seminar** | **Instructions for seminar presenter** |
| --- | --- | --- | --- |
| Week 1 (1 hour) | Introduction to the QI rotation (presented by QI Curriculum Director) | n/a | -Residents may complete QIKAT pre-test at beginning of seminar.  -Hand out QI workbooks.  -See slide set in Appendix C. |
| Week 2 (1.5 hours) | The essential QI toolbag: FOCUS-PDCAs and A3s (presented by QI Curriculum Director) | n/a | -Residents should bring QI workbooks to this seminar.  -See slide set in Appendix D. |
| Week 3 (1.5 hours) | Patient safety (presented by QI Curriculum Director) | n/a | -See slide set in Appendix E. |
| Week 4 (1 hour) | Principles of survey design for QI projects (presented by one of the directors of the university’s survey center) | AM last page: avoiding five common pitfalls of survey design. Artino AR, Gehlbach H, Durning SJ. Acad Med 2011;86(10):1327 | -See slide set in Appendix F. |
| Week 5 (1 hour) | QI in action: A successful case of improving psychiatric patient care as a resident (presented by a residency alum who conducted a model QI project when he completed this rotation, published the results of it, and is now a QI faculty supervisor within this curriculum) | Optional reading: SQUIRE guidelines for publishing QI studies. Available at: <http://www.squire-statement.org/> | -The seminar presenter presents the QI project he did for this rotation when he was a resident here, and describes the process he underwent to get his project published (including discussion of the Squire guidelines and of getting IRB exemption). |
| Week 6 (1 hour) | Root cause analysis (presented by the Psychiatry Department’s Hospital Peer Review Committee member) | Optional computer module:  Institute for Healthcare Improvement Module PS 201: Root Cause and Systems Analysis.  Available at: <http://app.ihi.org/lms/onlinelearning.aspx>  As residents complete the above module on the IHI Open School prior to seminar, they should think about a patient error they have made or have seen/heard about being made and how a root cause analysis could be used in that situation. | -The seminar presenter walks the residents through a root cause analysis based on a recent adverse event (suicide attempt) on the inpatient psychiatry unit. Materials are confidential and cannot be shared outside of the institution. |
| Week 7 (1 hour) | Maintenance of certification and performance in practice modules (presented by QI Curriculum Director and Residency Program Director) | Sample American Board of Psychiatry and Neurology Peer Feedback Form, Patient Feedback Form, 360 Feedback Form, and Clinical Chart Review Modules are made available to residents for use in completion of their homework assignment. Peer, Patient, and 360 Feedback forms are available as follows: Patient or Peer Feedback Module. American Board of Psychiatry and Neurology. <https://www.abpn.com/maintain-certification/moc-activity-requirements/improvement-in-medical-practice-pip/patient-or-peer-feedback-module/>. Updated 2018. Accessed December 3, 2018. Clinical Chart Review Modules (optional) are available as follows: Clinical Chart Review Module. American Board of Psychiatry and Neurology. <https://www.abpn.com/maintain-certification/moc-activity-requirements/improvement-in-medical-practice-pip/clinical-chart-review-module/>. Updated 2018. Accessed December 3, 2018. | -See slide set in Appendix G. |
| Week 8 (1 hour) | Involving stakeholders: how to run a successful meeting (presented by QI Curriculum Director) | Residents should think ahead of time about meetings they have attended or led, and one thing that makes them go well, and one thing that makes them go poorly. | -See slide set in Appendix H. |
| Week 9 (1 hour) | QI Journal Club (presented by QI Curriculum Director) | Wiechers IR, Viron M, Stoklosa J, Freudenreich O, Henderson DC, Weiss A. Impact of a metabolic screening bundle on rates of screening for metabolic syndrome in a psychiatry resident outpatient clinic. Acad Psychiatry. 2012;36:118-121.  Fan E, Laupacis A, Pronovost PJ, Guyatt GH, Needham DM. Users’ guides to the medical literature: how to use an article about quality improvement. JAMA. 2010;304(20):2279-2287. | -Residents should think ahead of time about how the assigned reading (Wiechers IR, et al.) does or does not meet the standards discussed in the JAMA Users’ Guide (Fan E, et al.). The seminar presenter leads a discussion of this. |
| Week 25 (1 hour) | QI midpoint project review (Supervised by QI Curriculum Director) | n/a | -Each resident team come prepared to give a 10-minute update on QI projects. Feedback is provided by classmates. |
| Week 50 (1.5 hours) | Performance in practice module presentations (Supervised by QI Curriculum Director and Residency Program Director) | n/a | -Residents may complete QIKAT post-test at beginning of seminar.  -Each resident come prepared to give a 10-minute overview of their experiences/results with the PIP module they completed (i.e., from the homework assignment given during week 7). |
