# Supplementary material for: A Quality Improvement Curriculum for Psychiatry Residents
Source: MedEdPORTAL. 2020 Jan 24;16:10870. doi: 10.15766/mep_2374-8265.10870 (PMC7012317; doi:10.15766/mep_2374-8265.10870)
Supplement: Supplementary file 1 — A. QI Didactic Seminars.doc B. Introduction to the QI Rotation Slides.ppt C. Essential QI Toolbag Slides.ppt D. Patient Safety Slides.ppt E. Principles of Survey Design Slides.pptx F. CBC and PIP Modules Slides.pptx G. Involving Stakeholders Slides.ppt H. QIKAT for Psychiatry.doc I. QI Workbook.doc J. QI Final Presentation Guidelines.doc K. A3 QI Poster Template 11x17.pptx L. QI Supervisor Evaluation of Resident.docx M. QI Director Evaluation of Resident.pdf N. QI Facts of the Week Sample.docx [file mep-16-10870-s001.zip › H. QIKAT for Psychiatry.doc]

**QIKAT: Quality Improvement Knowledge Application Tool**

Thank you for taking the time to answer these questions. We are assessing residents’ ability to read a scenario and then (a) identify systems-based errors, (b) propose an intervention, (c) clearly state an aim, and (d) indicate appropriate measures for assessing effectiveness.

This evaluation will be completed at the start of the Quality Improvement didactic seminar series and again at the end. Your responses to the scenarios will be graded according to a standard scoring form. Your responses are entirely confidential and anonymous. While the collective results of this tool may be published or presented in scientific venues, no individuals would be identified.

This evaluation is based on adaptation of the QIKAT questionnaire developed at Dartmouth and used for their Internal Medicine residents participating in a Quality Improvement rotation (Morrison LJ, Headrick LA, Ogrinc G, Foster T. The quality improvement knowledge application tool (QIKAT): an instrument to assess knowledge application in practice-based learning and improvement [abstract]. J Gen Intern Med. 2003;18(suppl):250.).

.

Instructions: Please read each of the following two clinical scenarios and answer the five questions that follow each one. We recognize that there may be many areas to improve in each scenario. We request that you provide a response to each question, even if you are unsure of the single best response.

Scenario #1

You are a general psychiatrist in a three-person practice and have just finished a busy morning clinic session. Your last patient was a 60 year-old man with bipolar disorder, hypercholesterolemia, and type 2 diabetes mellitus. His bipolar disorder is well-controlled with quetiapine 600 mg po qhs and lithium carbonate 300 mg po BID. However, you are frustrated because his hypercholesterolemia and diabetes remain sub-optimally controlled. He is not interested in exercise, and his diet is questionable. Quetiapine and lithium in combination has been the only medication regimen that has sufficiently controlled his mood disorder.

As you sit down to ponder his case, you open a letter from one of the insurance plans that covers many of your patients. Enclosed is a summary of their review of a random number of patients in your practice for whom you prescribe atypical antipsychotics. This was done as part of their annual review for National Committee on Quality Assurance certification of their plan. The data show that on several measures (frequency of fasting glucose and fasting lipid testing, frequency of weight and waist circumference measurement) more than 65% of your patients do not meet their target goals for patients on atypical antipsychotics. This further adds to your level of frustration and ruins your appetite for lunch.

Questions for Scenario #1

1) Clearly state the aim of a quality improvement intervention you could undertake with regard to a clinically relevant outcome.
2) What would you measure to assess the situation?
3) Identify one change that might be worth testing.

Scenario #2

You are a psychiatry intern on a busy inpatient unit and are starting your morning by administering electroconvulsive therapy to three of your patients. You are preparing to administer ECT treatment #1 to your patient Ms. A, who had previously shown a nice response to ECT treatment several years ago. All goes well with the treatment, as Ms. A has a 50-second seizure. However, you notice that she seems to have more oral secretions at the end of the procedure than you normally see in your ECT patients. You worry that she might not have received her pre-ECT glycopyrrolate, and to your horror, you discover that you failed to order it for her when you put in your ECT orders the prior evening. Worse yet, she develops an aspiration pneumonia that requires transfer to the ICU for several days. In talking with another of your intern colleagues, you discover that she too once forgot to order glycopyrrolate for one of her ECT patients, though fortunately that patient did not aspirate.

It has generally been standard practice for psychiatry residents at your hospital to enter into the computerized medical record system a set of individual orders for patients who are to receive ECT. This is usually done on the day prior to their first ECT treatment in a course of ECT. These orders usually include NPO directions, glycopyrrolate orders, and orders to hold benzodiazepines prior to each ECT treatment. It doesn’t seem to you that it has ever been too difficult to remember to place all of these orders for each patient about to start ECT, but sometimes, as happened here, things can become very busy on the unit so that you end up hastily putting in orders without the opportunity to double-check them. In thinking further about this, you start to wonder about all the other things you might have forgotten to order for patients this year without even realizing it.

Questions for Scenario #2

1) Clearly state the aim of a quality improvement intervention you could undertake with regard to a clinically relevant outcome.
2) What would you measure to assess the situation?
3) Identify one change that might be worth testing.

Instructions for scoring scenario responses

When scoring, please consider the following factors:

- Do the answers incorporate improvement fundamentals (customer focus, process knowledge, small tests of change/PDSA)?
- Do the three elements (aim, measure, change) bear some relationship to each other?

Use the guide below to assign scores:

0 = no response

1 = attempted, but way off the mark; highest possible score if only one element addressed

2 = needs substantial modification; elements unrelated; highest possible score if only two elements addressed

3 = good; needs modification; elements poorly related

4 = very good; needs minimal modification; elements related

5 = excellent; no modification needed; elements clearly related
