# Supplementary material for: A Quality Improvement Curriculum for Psychiatry Residents
Source: MedEdPORTAL. 2020 Jan 24;16:10870. doi: 10.15766/mep_2374-8265.10870 (PMC7012317; doi:10.15766/mep_2374-8265.10870)
Supplement: Supplementary file 1 — A. QI Didactic Seminars.doc B. Introduction to the QI Rotation Slides.ppt C. Essential QI Toolbag Slides.ppt D. Patient Safety Slides.ppt E. Principles of Survey Design Slides.pptx F. CBC and PIP Modules Slides.pptx G. Involving Stakeholders Slides.ppt H. QIKAT for Psychiatry.doc I. QI Workbook.doc J. QI Final Presentation Guidelines.doc K. A3 QI Poster Template 11x17.pptx L. QI Supervisor Evaluation of Resident.docx M. QI Director Evaluation of Resident.pdf N. QI Facts of the Week Sample.docx [file mep-16-10870-s001.zip › I. QI Workbook.doc]

**2018-19 Quality Improvement**

**Rotation and Seminar Series**

**Project Workbook**

**(fill in name of institution here)**

**Psychiatry Residency Program**

**(fill in resident’s name here)**

**QI Project Supervision Meeting Times and Location:**

**(fill in times/location here)**

**Other Important Dates (PLEASE PUT IN YOUR CALENDAR!):**

**M&M Pre-Meeting Presentation of Patient Case: (fill in date/time here)**

**M&M Presentation of Patient Case: (fill in date/time here)**

**Final Grand Rounds QI Project Presentation: (fill in date here)**

**Midpoint QI Project Presentations: (fill in date/time here)**

**Performance in Practice Module presentations: (fill in date/time here)**

# Worksheet A:

# Team Meeting Notes

| **Date** | **Main points of discussion** | **Next steps** | **Person Responsible** | **Due by:** |
| --- | --- | --- | --- | --- |
|  |  |  |  |  |
|  |  |  |  |  |
|  |  |  |  |  |

# Worksheet A:

# Team Meeting Notes

| **Date** | **Main points of discussion** | **Next steps** | **Person Responsible** | **Due by:** |
| --- | --- | --- | --- | --- |
|  |  |  |  |  |
|  |  |  |  |  |
|  |  |  |  |  |

# Worksheet A:

# Team Meeting Notes

| **Date** | **Main points of discussion** | **Next steps** | **Person Responsible** | **Due by:** |
| --- | --- | --- | --- | --- |
|  |  |  |  |  |
|  |  |  |  |  |
|  |  |  |  |  |

# Worksheet A:

# Team Meeting Notes

| **Date** | **Main points of discussion** | **Next steps** | **Person Responsible** | **Due by:** |
| --- | --- | --- | --- | --- |
|  |  |  |  |  |
|  |  |  |  |  |
|  |  |  |  |  |

# Worksheet A:

# Team Meeting Notes

| **Date** | **Main points of discussion** | **Next steps** | **Person Responsible** | **Due by:** |
| --- | --- | --- | --- | --- |
|  |  |  |  |  |
|  |  |  |  |  |
|  |  |  |  |  |

# Worksheet A:

# Team Meeting Notes

| **Date** | **Main points of discussion** | **Next steps** | **Person Responsible** | **Due by:** |
| --- | --- | --- | --- | --- |
|  |  |  |  |  |
|  |  |  |  |  |
|  |  |  |  |  |

# Worksheet A:

# Team Meeting Notes

| **Date** | **Main points of discussion** | **Next steps** | **Person Responsible** | **Due by:** |
| --- | --- | --- | --- | --- |
|  |  |  |  |  |
|  |  |  |  |  |
|  |  |  |  |  |

# Worksheet A:

# Team Meeting Notes

| **Date** | **Main points of discussion** | **Next steps** | **Person Responsible** | **Due by:** |
| --- | --- | --- | --- | --- |
|  |  |  |  |  |
|  |  |  |  |  |
|  |  |  |  |  |

# Worksheet A:

# Team Meeting Notes

| **Date** | **Main points of discussion** | **Next steps** | **Person Responsible** | **Due by:** |
| --- | --- | --- | --- | --- |
|  |  |  |  |  |
|  |  |  |  |  |
|  |  |  |  |  |

# Worksheet A:

# Team Meeting Notes

| **Date** | **Main points of discussion** | **Next steps** | **Person Responsible** | **Due by:** |
| --- | --- | --- | --- | --- |
|  |  |  |  |  |
|  |  |  |  |  |
|  |  |  |  |  |

# Worksheet A:

# Team Meeting Notes

| **Date** | **Main points of discussion** | **Next steps** | **Person Responsible** | **Due by:** |
| --- | --- | --- | --- | --- |
|  |  |  |  |  |
|  |  |  |  |  |
|  |  |  |  |  |

# Worksheet A:

# Team Meeting Notes

| **Date** | **Main points of discussion** | **Next steps** | **Person Responsible** | **Due by:** |
| --- | --- | --- | --- | --- |
|  |  |  |  |  |
|  |  |  |  |  |
|  |  |  |  |  |

# Worksheet A:

# Team Meeting Notes

| **Date** | **Main points of discussion** | **Next steps** | **Person Responsible** | **Due by:** |
| --- | --- | --- | --- | --- |
|  |  |  |  |  |
|  |  |  |  |  |
|  |  |  |  |  |

# Worksheet A:

# Team Meeting Notes

| **Date** | **Main points of discussion** | **Next steps** | **Person Responsible** | **Due by:** |
| --- | --- | --- | --- | --- |
|  |  |  |  |  |
|  |  |  |  |  |
|  |  |  |  |  |

# Worksheet B:

# Aim Setting

- An aim is a clear statement of the intended improvement and how it is to be measured.
- Use your aim statement to stay focused, to establish boundaries for what is and is not included in your team’s work, and to define success.
- Post your aim where it is visible at every team meeting.

**Write an aim for improvement:**

- Your aim should:
- Be measurable (Answers the question “How good?”)
- Be time-specific (Answers the question “By when?”)
- Describe the specific population of patients who would be measured (Answers the question “For whom?”)
- Does *not* include *how* you will achieve goal.
- May change with time as you gather more information.

Example: By December, 75% of patients at WisPIC who currently have a substance use disorder will have been referred for formal substance use disorder treatment.

# Worksheet C: Determining Measures

- Use of measures help you to determine when a change is an improvement.
- QUANTITATIVE measures often provide the best feedback.
- QI teams usually use 3 types of measures:
- Outcomes measures: Where are we ultimately trying to go? What is the ultimate result?
- Process measures: Are we doing the right things to get there?
- Balancing measures: Are the changes we are making to one part of the system causing problems in other parts of the system?

*Example*:

| Aim | Outcome Measures | Process Measures | Balancing Measures |
| --- | --- | --- | --- |
| By December, 75% of patients in our outpatient clinic who currently have a substance use disorder will have been referred for formal AODA treatment. | % of our outpatients with substance use disorder who are referred for formal AODA treatment (though of course, the ULTIMATE outcome measure is % of patients with substance use disorder whose condition remits) | % of our staff members who ask their patients about substance use/misuse | Is an increase in referrals to AODA treatment associated with less thorough safety evaluations?  Is an increase in referrals to AODA treatment associated with lower patient satisfaction? |

*Now write your own measures (definitely don’t need to include all of them if not relevant or your primary focus):*

| Aim | Outcome Measures | Process Measures | Balancing Measures |
| --- | --- | --- | --- |
|  |  |  |  |

# Worksheet D: Determining What Part of a Process to Change

- You will probably need to test several different changes (one at a time!), since it’s hard to know which will achieve your aim.
- Basic techniques to come up with good ideas for change:
- Current process analysis (see Worksheet E: Current Process Analysis)
- Benchmarking (compare your process to “best practices”, which can be ascertained from the literature, survey of other clinics in town, etc)
- Using technology (automation—think of changes in med records templates, etc, or new equipment)
- Take on the role of the patient (do a walk-through of the patient experience!)
- Ask other staff members if they have ideas for improvement
- Ask patients if they have ideas for improvement
- Consider if error proofing (checklists!) would help. Human memory isn’t that great, so we shouldn’t rely on it!

Now write some changes you could try to implement to see if they help you achieve your aim (again, of course you would only test one change at a time):

1.

2.

3.

4.

5.

# Worksheet E: Current Process Analysis

- A process is a series of activities or steps that is meant to achieve a particular result.
- When defining a process, think about staff roles in the process, the tools or materials staff use, and the flow of activities.
- Everything is a process, whether it is admitting a patient, serving meals, assessing suicidality, or running an inpatient group. The ultimate goal of defining a process is identifying problems in the current process.

Have the team identify and define every step in the current process that you have chosen to improve:

***Tips:***

# Take time to “brainstorm” and listen to every team member.

- The process must be understood and documented.

# Make each step in the process very specific.

- Lay out each step, move steps, and add and remove steps until the team agrees on final process.
- If the problem is that a process does not exist *(for example, there is no current process to use an objective rating scale to evaluate symptoms of depression on admission)*, then identify the related processes *(for example, the entire process for admission).*
- If the process is different for different shifts/providers, identify each individual process.

## Example: Process for making buttered toast

| Step | **Define** |
| --- | --- |
| 1 | Check to see if there is bread, butter, knife, and toaster. |
| 2 | If supplies are missing, go to the store and purchase them. |
| 3 | Check to see if the toaster is plugged in – if not, plug in the toaster. |
| 4 | Check setting on toaster – adjust to darker or lighter as preferred. |
| 5 | Put a slice of bread in toaster. |
| 6 | Turn toaster on. |
| 7 | Wait for bread to toast. |
| 8 | When toast is ready, remove from toaster and put on plate. |
| 9 | Use knife to cut pat of butter. |
| 10 | Use knife to spread butter on toast. |

**Current Process Analysis (cont.)**

**Now write the steps of your defined process below:**

| Step | **Define** |
| --- | --- |
| 1 |  |
| 2 |  |
| 3 |  |
| 4 |  |
| 5 |  |
| 6 |  |
| 7 |  |
| 8 |  |
| 9 |  |
| 10 |  |
| 11 |  |
| 12 |  |
| 13 |  |
| 14 |  |
| 15 |  |
| 16 |  |
| 17 |  |
| 18 |  |
| 19 |  |
| 20 |  |
| 21 |  |
| 22 |  |
| 23 |  |
| 24 |  |
| 25 |  |
| 26 |  |
| 27 |  |

**Current Process Analysis (cont.)**

**Evaluate your current process as you define it:**

What (official or unofficial) policies and procedures are in place for this process?

What forms do we use?

How does our physical environment support or hinder this process?

What staff are involved in this process?

What part of this process does not work?

Do we duplicate any work unnecessarily?

Are there any delays in the process? Why?

When you discover a problem in your current process, you may wish to continue to Worksheet F: Fishbone Diagram (and there will be a seminar on this as part of the QI rotation), to determine the most likely primary cause(s) of the problem.

# **Worksheet F:**

# **Fishbone Diagram**

##	**Environment**	**Equipment**	**People**

Main Problem in Process

**Methods/	Materials**

**Process**

# Worksheet G:

# PDCA Cycles (i.e., how to test the ideas for things to change that you brainstormed earlier)

- **Plan** (remember to start small to prove a change works before rolling it out to a larger setting or group of providers!)
- What will be the change we are testing?
- Who will be involved in the change? Consider attendings, other staff, residents, etc who may be affected by the change.
- When will the change be made (start date)?
- When will the change be evaluated?
- Where will the change take place?
- **Do**
- Carry out the plan
- Document problems and unexpected observations
- Begin analysis of the data (See Worksheet H: PDSA Data Collection)

# PDCA Cycles (cont.)

- **Check**
- Complete analysis of the data (See Worksheet H: PDSA Data Collection)
- Compare data to predictions
- What worked well?
- What did not work well?
- How do the involved parties (attendings, staff, residents, etc) feel about the process so far?
- **Act**
- What changes are to be made?
- Do we need to revise any of the materials we are using?
- Do we need to re-evaluate our initial aim?
- Prepare for the next PDSA cycle

# Worksheet G:

# PDCA Cycles

- **Plan** (remember to start small to prove a change works before rolling it out to a larger setting or group of providers!)
- What will be the change we are testing?
- Who will be involved in the change? Consider attendings, other staff, residents, etc who may be affected by the change.
- When will the change be made (start date)?
- When will the change be evaluated?
- Where will the change take place?
- **Do**
- Carry out the plan
- Document problems and unexpected observations
- Begin analysis of the data (See Worksheet H: PDSA Data Collection)

# PDSA Cycles (cont.)

- **Check**
- Complete analysis of the data (See Worksheet H: PDSA Data Collection)
- Compare data to predictions
- What worked well?
- What did not work well?
- How do the involved parties (attendings, staff, residents, etc) feel about the process so far?
- **Act**
- What changes are to be made?
- Do we need to revise any of the materials we are using?
- Do we need to re-evaluate our initial aim?
- Prepare for the next PDSA cycle

# Worksheet G:

# PDCA Cycles

- **Plan** (remember to start small to prove a change works before rolling it out to a larger setting or group of providers!)
- What will be the change we are testing?
- Who will be involved in the change? Consider attendings, other staff, residents, etc who may be affected by the change.
- When will the change be made (start date)?
- When will the change be evaluated?
- Where will the change take place?
- **Do**
- Carry out the plan
- Document problems and unexpected observations
- Begin analysis of the data (See Worksheet H: PDSA Data Collection)

# PDSA Cycles (cont.)

- **Check**
- Complete analysis of the data (See Worksheet H: PDSA Data Collection)
- Compare data to predictions
- What worked well?
- What did not work well?
- How do the involved parties (attendings, staff, residents, etc) feel about the process so far?
- **Act**
- What changes are to be made?
- Do we need to revise any of the materials we are using?
- Do we need to re-evaluate our initial aim?
- Prepare for the next PDSA cycle

# Worksheet G:

# PDCA Cycles

- **Plan** (remember to start small to prove a change works before rolling it out to a larger setting or group of providers!)
- What will be the change we are testing?
- Who will be involved in the change? Consider attendings, other staff, residents, etc who may be affected by the change.
- When will the change be made (start date)?
- When will the change be evaluated?
- Where will the change take place?
- **Do**
- Carry out the plan
- Document problems and unexpected observations
- Begin analysis of the data (See Worksheet H: PDSA Data Collection)

# PDSA Cycles (cont.)

- **Check**
- Complete analysis of the data (See Worksheet H: PDSA Data Collection)
- Compare data to predictions
- What worked well?
- What did not work well?
- How do the involved parties (attendings, staff, residents, etc) feel about the process so far?
- **Act**
- What changes are to be made?
- Do we need to revise any of the materials we are using?
- Do we need to re-evaluate our initial aim?
- Prepare for the next PDSA cycle

# Worksheet G:

# PDCA Cycles

- **Plan** (remember to start small to prove a change works before rolling it out to a larger setting or group of providers!)
- What will be the change we are testing?
- Who will be involved in the change? Consider attendings, other staff, residents, etc who may be affected by the change.
- When will the change be made (start date)?
- When will the change be evaluated?
- Where will the change take place?
- **Do**
- Carry out the plan
- Document problems and unexpected observations
- Begin analysis of the data (See Worksheet H: PDSA Data Collection)

# PDSA Cycles (cont.)

- **Check**
- Complete analysis of the data (See Worksheet H: PDSA Data Collection)
- Compare data to predictions
- What worked well?
- What did not work well?
- How do the involved parties (attendings, staff, residents, etc) feel about the process so far?
- **Act**
- What changes are to be made?
- Do we need to revise any of the materials we are using?
- Do we need to re-evaluate our initial aim?
- Prepare for the next PDSA cycle

# Worksheet H: PDCA Data Collection

**Has the change had an impact?**

*Example: 5 out of 5 new admissions have completed PHQ-9 forms within 24 hours*

| **Date** | **Chosen measure for evaluation (***i.e., PHQ-9 assessment for new admission is completed within 24 hours.)* | **# of cases reviewed**  **(A)** | **# of cases with positive results**  **(B)** | **B out of A**  **(B/A)** |
| --- | --- | --- | --- | --- |
|  |  |  |  |  |
|  |  |  |  |  |
|  |  |  |  |  |
|  |  |  |  |  |
|  |  |  |  |  |
|  |  |  |  |  |
|  |  |  |  |  |
|  |  |  |  |  |
|  |  |  |  |  |
|  |  |  |  |  |
|  |  |  |  |  |
|  |  |  |  |  |

**Conduct data collection during each PDSA cycle.**

# **Worksheet I: Ongoing Monitoring**

- After conducting several PDCA cycles and a satisfactory improvement has been observed, the goal becomes **permanent and sustainable** implementation of the change.
- Monitoring the implemented change allows your team to evaluate, on an ongoing basis, whether or not the implemented change has made an impact on overall care delivery.
- Decide who will perform tracking related to full (possibly facility-wide) implementation.
- Decide when this monitoring will be completed (i.e. monthly, bimonthly, quarterly).
- Decide how these data will be collected and evaluated.

**Date of facility-wide implementation (if applicable)**_______________

How will you know if you have achieved implementation?

Example: We will know when 10 out of 10 admissions this month show that a PHQ-9 assessment was completed upon admission.

**We will know when**____________________________________________________

**____________________________________________________________________**

***Record findings:***

| Date | # of cases reviewed (A) | # of cases with positive results (B) | B out of A  **(B/A)** |
| --- | --- | --- | --- |
|  |  |  |  |
|  |  |  |  |
|  |  |  |  |
|  |  |  |  |
|  |  |  |  |

**Review the following:**

1. Based on the data collected, check to see if the process has been implemented to a satisfactory extent. If it has, continue to monitor as long as the team feels necessary.

2. Based on the data collected, check to see if implementation of the new (improved) process has had an impact on the delivery of care. If it has not, you may wish to explore the following questions:

# **Ongoing Monitoring (cont.)**

Has the process been successful on some shifts or units, and not on others? With some providers and not others? If so, why?

Is further staff education needed? In what areas?

Does the process need to be revised if larger-scale (e.g., facility-wide) implementation is desired?
