# Supplementary material for: A Quality Improvement Curriculum for Psychiatry Residents
Source: MedEdPORTAL. 2020 Jan 24;16:10870. doi: 10.15766/mep_2374-8265.10870 (PMC7012317; doi:10.15766/mep_2374-8265.10870)
Supplement: Supplementary file 1 — A. QI Didactic Seminars.doc B. Introduction to the QI Rotation Slides.ppt C. Essential QI Toolbag Slides.ppt D. Patient Safety Slides.ppt E. Principles of Survey Design Slides.pptx F. CBC and PIP Modules Slides.pptx G. Involving Stakeholders Slides.ppt H. QIKAT for Psychiatry.doc I. QI Workbook.doc J. QI Final Presentation Guidelines.doc K. A3 QI Poster Template 11x17.pptx L. QI Supervisor Evaluation of Resident.docx M. QI Director Evaluation of Resident.pdf N. QI Facts of the Week Sample.docx [file mep-16-10870-s001.zip › J. QI Final Presentation Guidelines.doc]

**PGY-3 Quality Improvement Project Presentation Guidelines**

Congratulations—you are taking important steps to improve health care by undertaking a quality improvement (QI) project! After spending significant time and effort developing, implementing, and studying the results of your QI project, we want you to be able to share your results with the Department of Psychiatry, including all of the QI supervisors, the QI rotation director, and your classmates. Toward the end of the academic year (see your binder cover for exact date and time), each QI team will present their project for 30 minutes, including time for Q&A, during the usual Grand Rounds time. There will typically be 2 teams assigned per Grand Rounds date.

You should strive to include the following elements in your project presentation:

- Rationale for undertaking a project on the chosen topic, including discussion of quality gaps and problems with psychiatric care delivery
- Evidence that the project has some relationship to quality initiatives of WisPIC, UWHC, VA, APA, JCAHO, or other entity to which we are accountable
- Details of barriers to implementation or buy-in
- Evidence for stakeholder involvement in the process, and discussion of strategies for getting buy-in from stakeholders affected by the project
- Baseline data
- Specific aim statement (answers the questions: how good? by when? for whom?)
- Describes methods for implementation of project, including details of utilization of at least one QI tool (e.g., PDSA cycle, root cause analysis, fishbone diagram, flow/process mapping, etc)
- What specifically was measured to know if the intervention(s) resulted in an improvement, including comparison of baseline and final data. Data may be presented in table/graph format and could include outcome measures, process measures, and balancing measures.
- Sustainability of the intervention even after the resident team completes their QI rotation
- A3 poster (should be hung up on the white board with large clips provided in the Grand Rounds room).

Your presentation will be evaluated based on the above elements.
